# Rhodium(i) complexes derived from tris(isopropyl)-azaphosphatrane—controlling the metal–ligand interplay†

**DOI:** 10.1039/d1ra07126b

**Published:** 2021-11-22

**Authors:** Wei-Chieh Chang, Fritz Deufel, Thomas Weyhermüller, Christophe Farès, Christophe Werlé

**Affiliations:** Max Planck Institute for Chemical Energy Conversion Stiftstr. 34–36 45470 Mülheim an der Ruhr Germany christophe.werle@cec.mpg.de; Ruhr University Bochum Universitätsstr. 150 44801 Bochum Germany; Max-Planck-Institut für Kohlenforschung Kaiser-Wilhelm-Platz 1 D-45470 Mülheim an der Ruhr Germany

## Abstract

Proazaphosphatranes are intriguing ligand architectures comprising a bicyclic cage of flexible nature. They can undergo structural deformations due to transannulation while displaying modular electronic and steric properties. Herein, we report the synthesis and coordination chemistry of rhodium(i) complexes bearing a tris(isopropyl)-azaphosphatrane (T^i^PrAP) ligand. The molecular structure of the primary complex (1) revealed the insertion of the metal center into a P–N bond of the ligand. The addition of a Lewis acid, *i.e.*, lithium chloride, promoted the dynamic behavior of the complex in the solution, which was studied by state-of-the-art NMR spectroscopy. Substituting the cyclooctadiene ligand at the metal center with triphenylphosphine or 2-pyridyldiphenylphosphine unveiled the adaptive nature of the T^i^PrAP backbone capable of switching its axial nitrogen from interacting with the phosphorus atom to coordinate the rhodium center. This led the entire ligand edifice to change its binding to rhodium from a bidentate to tridentate coordination. Altogether, our study shows that introducing a T^i^PrAP ligand allows for unique molecular control of the immediate environment of the metal center, opening perspectives in controlled bond activation and catalysis.

## Abstract 2

The synthesis and coordination chemistry of Rh(i) complexes bearing a tris(isopropyl)-azaphosphatrane (T^i^PrAP) ligand are reported. The adaptive nature of T^i^PrAP ligands allows for molecular control of the immediate environment of the metal center.

## Graphical abstract

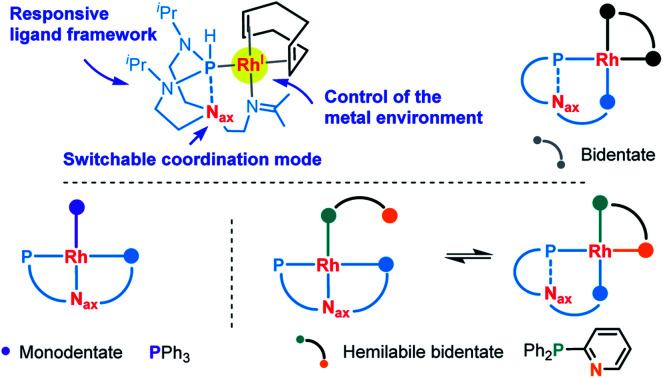

## Introduction

In catalysis, finding the right metal–ligand combination to regulate the properties of the resulting system and thus meet the requirements of a specific application is a continuous quest.^1,2^ For many years, the focus has been on the transition metal; the ligands regarded as spectators were supposed to remain unchanged throughout the lifetime of the catalyst. Research to enhance catalyst activity and selectivity has identified ligands as essential design elements.^3^ Not only do they exert a steric and electronic influence on the complex, but they are also capable of acting in cooperation with their metal.^4,5^ As an intrinsic part of the local environment of the metal center, ligands have a significant influence on bond activation processes and catalysis.^6^

**Fig. 1:**
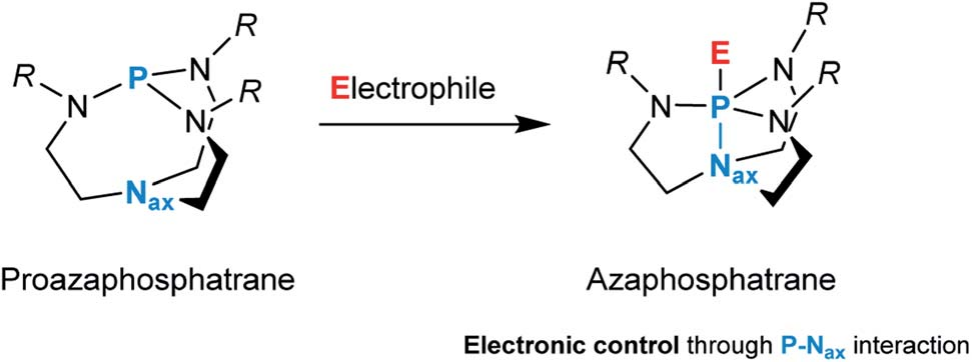
A reaction of a proazaphosphatrane with an electrophile.

In this context, the proazaphosphatrane unit composed of a conformationally flexible bicyclic cage, more commonly referred to as Verkade's superbase, represents an intriguing ligand design.^7^ This architecture is susceptible to structural deformation caused by transannulation, *i.e.*, an intramolecular interaction between the axial nitrogen (N_ax_) and phosphorus upon binding to an electrophile which results in the formation of an azaphosphatrane (Fig. 1).

Consequently, proazaphosphatranes have attracted considerable interest as stoichiometric or catalytic Lewis bases for various chemical transformations, among which: cyanosilylation of aldehydes and ketones^8^ or imines,^9^ selective monoalkylation of active-methylene compounds,^10^ dehydrohalogenation reactions,^11^ transesterifications, acylations and deacylations,^12^ the synthesis of β-hydroxy nitriles,^13^ or Henry reaction,^14^ to name a few.

Although the reactivity of proazaphosphatranes has been studied considerably, their coordination chemistry remains largely in its infancy. In this context, Yang and coworkers experimentally determined both Tolman electronic parameters (TEPs) and cone angles for a series of nickel tricarbonyl proazaphosphatrane complexes.^15^ They found that proazaphosphatranes present variable cone angles depending on the substituents on the equatorial nitrogen (N_eq_). Additionally, when compared to substituted tertiary phosphine, a higher donor strength was observed, which can be attributed primarily to the presence of amino substituents. In another study, the authors showed that the degree of transannular interaction increases when proazaphosphatrane binds to metal centers with a higher degree of electron deficiency.^16^ Complementarily, Martinez and coworkers showed by employing DFT calculations that the electron-donating ability of tris(methyl)-azaphosphatrane is better than usual phosphines and similar to N-heterocyclic carbene ligands (NHCs).^17^

In terms of supporting ligands, proazaphosphatranes and other relevant derivatives have been reported to support their respective metal effectively, as illustrated in cross-coupling reactions.^18^ Specifically, in their contribution, Johnson and coworkers demonstrated the catalytic relevance of palladium proazaphosphatrane complexes as putative intermediates in C–N cross-couplings.^19^ The proazaphosphatrane moiety was found to respond to changes in the metal's oxidation state and coordination sphere. The presence of variable transannulation in these complexes unveiled that proazaphosphatranes may accommodate conformational modifications to stabilize catalytic intermediates.^18*a*,20^ In a subsequent report, Johnson, Donald, and coworkers studied the factors that influence transannulation.^21^. They identified that the identity of the electrophile, the substitution at the N_eq_, and the oxidation state of phosphorus dictate the extent of transannulation. Additionally, they found that ethylene linkers are essential when targeting a strong interaction between the donor (N_ax_) and acceptor (P) within the molecule.

As part of our research program aimed at developing direct molecular control over bond activation processes and catalysis,^22^ we speculated that proazaphosphatrane ligands would be attractive molecular handles. The application of these ligands may provide an opportunity for deliberate regulation of the metal's immediate environment by varying the extent of transannulation throughout the catalytic process.

## Results and discussion

**Fig. 2:**
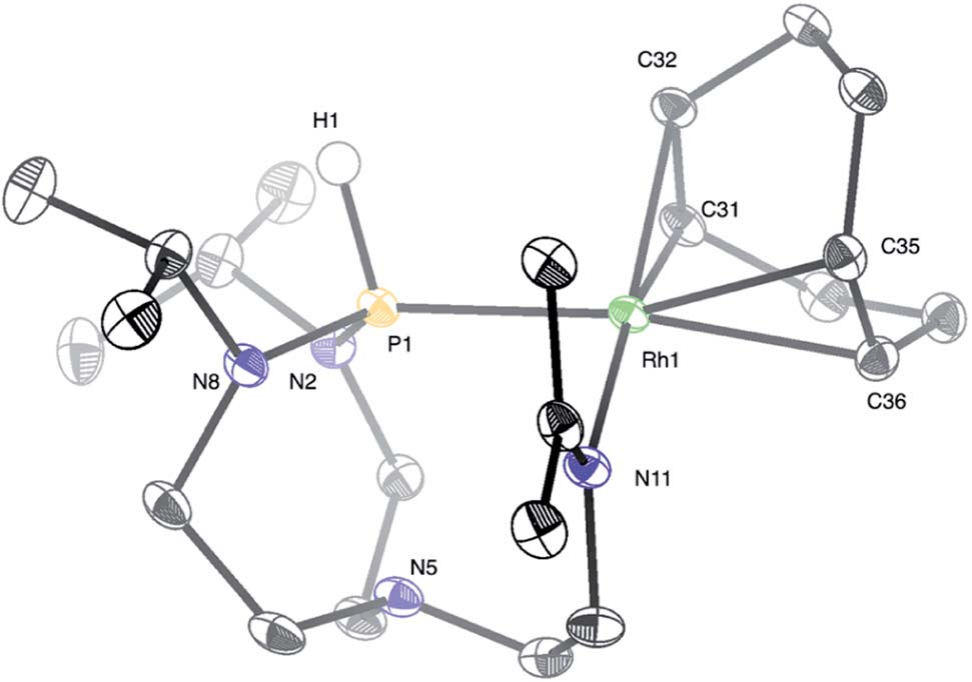
Molecular structure of complex 1 in the solid-state. The SbF_6_ anion and protons were removed for clarity, except for the proton on phosphorus. Selected interatomic distances (Å) and angles (°): Rh1–P1 2.3042(4), Rh1–N11 2.1091(12), P1–N5 2.608(1), Rh1–C31 2.1403(14), Rh1–C32 2.1441(13), Rh1–C35 2.2302(14), Rh1–C36 2.2577(14), N11–Rh1–P1 90.30(3), N8–P1–Rh1 122.14(5), N2–P1–Rh1 115.87(4), N2–P1–N8 112.26(6), Rh1–P1–N5 84.68(3), H1–P1–N5 168.4(8).

**Scheme 1:**
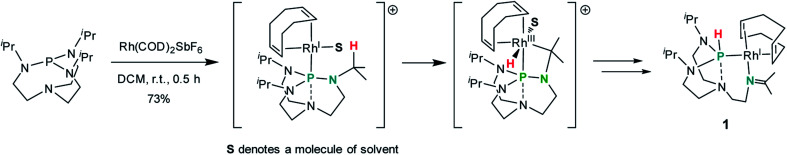
Efforts to rationalize the formation of the complex 1. The anion SbF_6_ is omitted for clarity.

Treatment of a dichloromethane solution of Rh(COD)_2_SbF_6_ (COD = 1,5-cyclooctadiene) with stoichiometric amounts of T^i^PrAP at room temperature led to the formation of an unprecedented complex (1) whose molecular identity could be accessed by NMR spectroscopy and X-ray diffraction analysis. The solid-state structure of 1 revealed a rhodium(i) center in a square planar geometry, bearing the T^i^PrAP skeleton and a COD moiety both coordinated in a bidentate manner to the metal center (Fig. 2). The unusual architecture of 1 may result from a tandem reaction under the premise of a phosphorus-directed C–H bond activation,^23^ followed by a hydride transfer reaction and structural rearrangement (Scheme 1).^24^ Similar breakdowns of the proazaphosphatrane core, resulting in the formation of a transannulated species lacking a third amido donor at the phosphorus atom, remain rare in the literature^25^ and have not been observed for a similar system involving [Rh(CO)Cl]_2_ as the precursor.^17^

Structural characterization of complex 1 by means of X-ray diffraction analysis shows a Rh1–P1 bond (2.3042(4) Å) falling into a single bond range, only slightly shorter (*ca.* 0.030–0.207 Å) than other representative Rh–phosphido bonds reported in the literature.^26^ The P1 atom nearly lies in the plane of Rh1–N2–N8. The sum of three angles around the phosphorus atom (Rh1–P1–N2, Rh1–P1–N8, and N2–P1–N8) equals 350.26°, expressing a pseudo-trigonal bipyramidal geometry around the phosphorus atom, which deviates from the more common tetrahedral geometries of phosphines. The interatomic distance between P1 and N5 (2.608(1) Å) is longer than for similar azaphosphatranes,^7*f*,27^ but shorter than in the T^i^PrAP starting material,^27*a*^ suggesting an intermediate transannular interaction between P1 and N5 atoms. Another interesting structural feature is given by the short N11–C18 bond length (1.2837(19) Å) and the trigonal planar nature of C18, advocating for an imine functional group. Finally, a *trans*-effect of P1 is evidenced by an increased length of the Rh1–C35 and Rh1–C36 bonds, compared to the shorter bond lengths found for the Rh1–C31 and Rh1–C32 bonds located *trans* to N11.

**Fig. 3:**
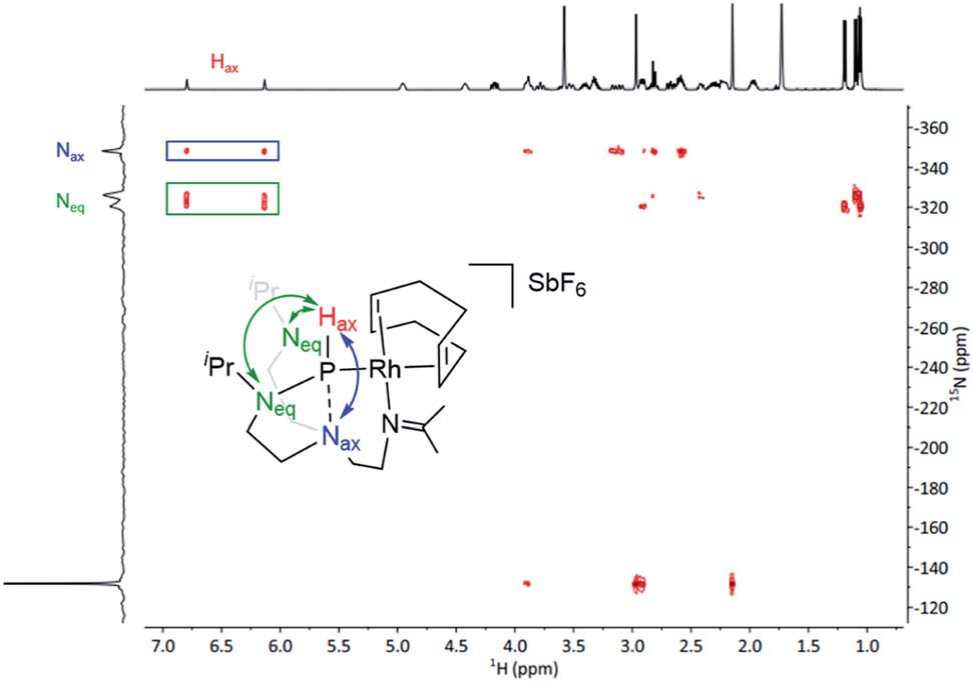
^1^H–^15^N HMBC spectrum of complex 1. The double-headed arrow indicates the long-range 2-bond correlations.

^1^H NMR spectroscopy further asserts the asymmetric character of complex 1 and is in good agreement with the solid-state molecular structure. Notably, the ^1^H NMR spectrum displays a characteristic doublet at 6.36 ppm with a large coupling constant (^1^*J*_P,H_ = 331 Hz), resolving in a singlet upon ^31^P decoupling in the ^1^H{^31^P} NMR experiment. This large coupling constant shows a direct attachment of a proton to the phosphorus nuclei and can be confirmed by infrared spectroscopy, where the characteristic band at 2209 cm^−1^ corresponds to the stretching frequency of the P–H bond (see Fig. S13 in the ESI†). The doublet at 178.4 ppm in the ^13^C{^1^H} NMR spectrum confirms the formation of an imine group. Additionally, the corresponding ^31^P{^1^H} NMR shows a doublet signal with a coupling constant ^1^*J*_Rh,P_ = 201.5 Hz, which is in the range of typical Rh–P couplings of square planar rhodium complexes.^28^ Importantly, ^1^H–^15^N HMBC spectrum unveiled strong correlations of the proton located at the phosphorus atom with the N_eq_ and the N_ax_ (both over a two-bond distance), suggesting the presence of a bond between the P and the N_ax_ (Fig. 3). Under these premises, *i.e.*, presence of a H_ax_ and simultaneous interaction with the N_ax_, the phosphorus atom exhibits a trigonal bipyramidal geometry.

**Fig. 4:**
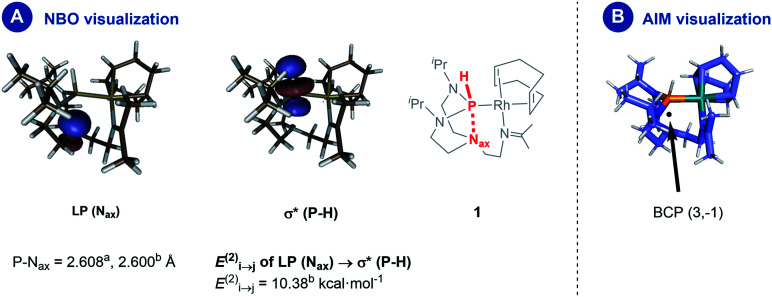
Panel (A) donor–acceptor interaction estimated by second-order perturbation theory; panel (B) the visualization of the bond critical point from the AIM analysis (further BCPs are omitted for clarity). ^*a*^Experimental value from the molecular structure as determined by X-ray diffraction analysis, ^*b*^theoretical value from DFT calculations.

A natural bond orbital (NBO) perturbation theory-based approach was chosen to characterize the P–N_ax_ interaction to gain further insights into this interaction. The LP(N_ax_) → σ*(P–H) stabilization is 10.38 kcal mol^−1^ (Fig. 4, panel A), which falls into the same range but is slightly stronger than the corresponding interaction in chloro- and iodo-azaphosphatranes (7.10 and 5.16 kcal mol^−1^, respectively).^21^ The bonding character is also verified by the atoms-in-molecules (AIM) method, revealing a bond critical point BCP (3,−1) located between the P and the N_ax_ atoms (Fig. 4, panel B).

**Fig. 5:**
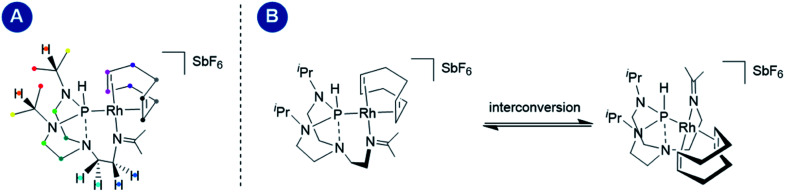
Panel (A) schematic representation of complex 1 with nuclei undergoing mutual exchange depicted in the same color; panel (B) shows the dynamic intramolecular interconversion occurring within complex 1.

On the EXSY time scale (visible in the 2D NOESY spectra, *t*_mix_ = 1 s, 298 K; see Fig. S4 in the ESI†), exchange peaks between the different protons depicted in identical colors were observed (Fig. 5, panel A), indicating a structural interconversion as illustrated in Fig. 5, panel B.

**Fig. 6:**
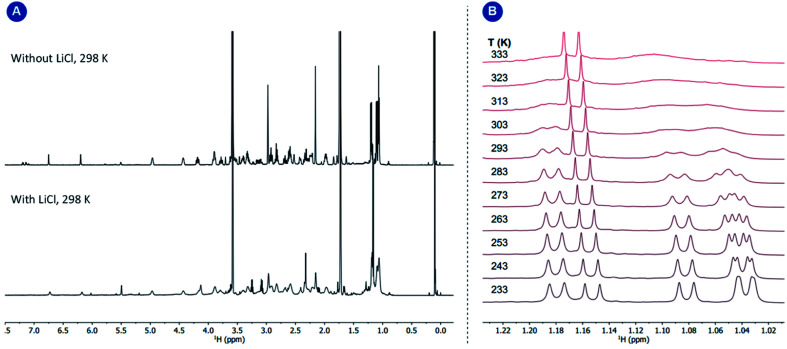
Panel (A) ^1^H NMR spectra of complex 1 with and without LiCl; panel (B) variable temperature ^1^H NMR study of complex 1 in the presence of LiCl (14 equiv.). The temperature-independent sharp signal corresponds to the minor amounts of the protonated T^i^PrAP.

Variable temperature 1D EXSY experiments provided access to the rate constant, activation entropy and enthalpy for the intramolecular interconversion (see Section 5 in the ESI†). The obtained values correspond to a concentration-independent equilibrium of approximately *k*_293.2 K_ = 0.113 ± 0.018 s^−1^ with an activation enthalpy of Δ*H*^‡^ = 18.5 ± 2.0 kcal mol^−1^ and an activation entropy Δ*S*^‡^ = −0.8 ± 6.4 cal mol^−1^ K^−1^ according to Eyring's theory.^29^ Given that the interconversion, shown in Fig. 5, panel B, requires ligand dissociation and recoordination of either the imine, phosphine, or cyclooctadiene ligand, we questioned whether the rate of ligand dissociation could be increased by the addition of a Lewis acid capable of interacting with one of the electron-donating atoms present in the complex and thus facilitate the process. The addition of lithium chloride (14 equiv.) resulted in a considerable line broadening of the peaks in the ^1^H NMR spectrum (Fig. 6, panel A). This is illustrated by the complete coalescence of the methyl peaks of the isopropyl groups at 313 K (Fig. 6, panel B).

**Fig. 7:**
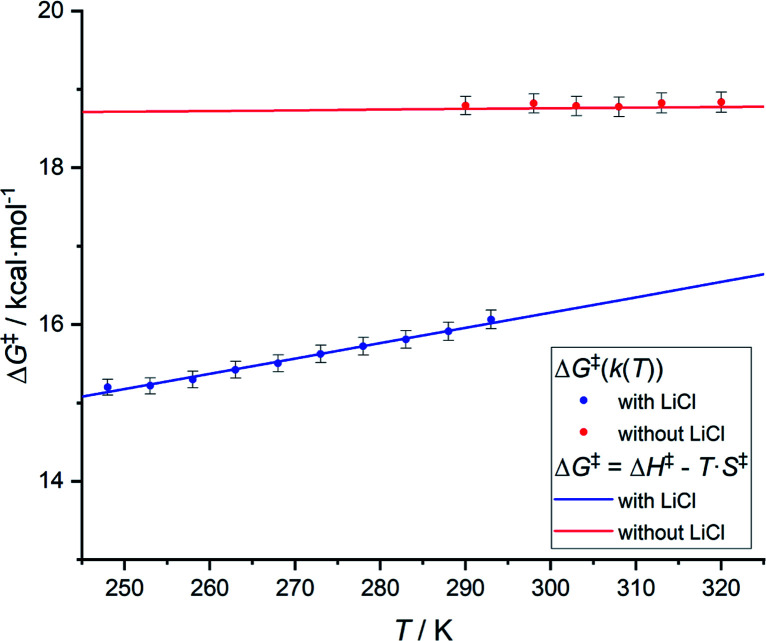
Graph showing the free energy of activation for the intramolecular interconversion of complex 1 with and without lithium chloride as a function of temperature.

Based on variable temperature ^1^H NMR experiments, in the presence of lithium chloride (14 equiv.), an activation enthalpy Δ*H*^‡^ = 10.3 ± 1.0 kcal mol^−1^ and entropy of Δ*S*^‡^ = −19.5 ± 3.6 cal mol^−1^ K^−1^ could be determined. The resulting lower activation enthalpy value illustrates that LiCl supports the dynamic interconversion process. Besides, the decreased and negative activation entropy suggests an associative mechanism, where the interconversion process might involve lithium coordination to the complex on one of the chelating groups (*e.g.*, tertiary nitrogen, phosphorus center). Apart from that, a hypothetic associative mechanism through the formation of a square-pyramidal geometry with chloride coordination to the rhodium center cannot be ruled out at this stage. Fig. 7 summarizes the free activation energy for the intramolecular interconversion of complex 1 with and without lithium chloride as a function of temperature.

This result is important for homogeneous catalysis, where the generation of inorganic salts as a byproduct is well-documented.^30^ Their presence in the reaction medium may radically change the performance of a catalyst by accelerating ligand coordination and dissociation and affecting the reaction kinetics of a given transformation.^31^

In attempts to further control the environment around the rhodium center, we hypothesized that replacing the bidentate COD ligand in complex 1 with a monodentate ligand might trigger the reorganization of the T^i^PrAP ligand framework. This was done by selecting triphenylphosphine as a candidate, carrying a soft phosphorus donor atom in high affinity with the soft rhodium center.

**Scheme 2:**
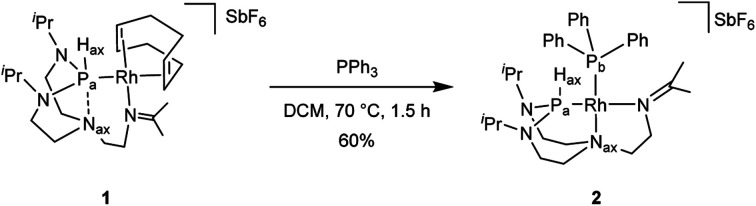
Preparation of complex 2.

Reacting complex 1 with a stoichiometric amount of triphenylphosphine in dichloromethane at 70 °C led to complex 2 (Scheme 2).

Coordination of the triphenylphosphine ligand is evidenced by ^31^P{^1^H} NMR spectroscopy and the presence of two doublets of doublets. The coupling constants ^1^*J*_P_a___,Rh_ = 163 Hz and ^1^*J*_P_b___,Rh_ = 202 Hz are falling in the range of a typical square planar complex.^28^ The magnitude of the coupling constant ^1^*J*_P_a_,P_b__ = 60 Hz reveals a *cis* conformation. The ^13^C{^1^H} NMR spectrum hints at the coordination of the imine group to the rhodium center with a coupling constant ^2^*J*_C,Rh_ = 1.6 Hz, which is similar to the value observed for complex 1 (^2^*J*_C,Rh_ = 1.9 Hz). The square planar geometry of the rhodium(i) center is satisfied by the occupation of its fourth coordination site by the N_ax_, shifting from the phosphorus atom to the rhodium center. This is reflected by the absence of correlation peaks between the H_ax_ and N_ax_ atoms in the ^1^H–^15^N HMBC spectrum of complex 2 (see Fig. S26 in the ESI†). Moreover, the shift of the N_ax_ leads to a significant geometric change at the P_a_ atom, evolving from a trigonal bipyramid (complex 1) to a tetrahedral geometry (complex 2). This results in the higher s-character in P_a_–H_ax_ bond as shown by an increased coupling constant with the H_ax_ (from ^1^*J*_P_a_,H_ = 331 Hz in complex 1 to 413 Hz in complex 2). Besides, the protons close to the N_ax_ show strong correlations with the P_b_, further corroborating its *trans*-position to the triphenylphosphine as confirmed by the ^1^H–^31^P HMBC experiment (see Fig. S25 in the ESI†). Finally, a direct correlation of these protons (*i.e.*, H_ax_ and the protons close to the N_ax_) with the rhodium center at −8430 ppm in a ^1^H–^103^Rh-HMBC was observed (see Fig. S27 in the ESI†).

The ^1^H NMR spectrum suggests that complex 2 has a more symmetric molecular structure when compared to the structure of 1. It was initially hypothesized that a fast-intramolecular exchange averages signals on the NMR time scale. However, the variable temperature experiments indicate only a slight change in the ^1^H NMR spectra and a negligible line broadening for the signals in the ^13^C{^1^H} NMR spectra are observed (see Fig. S20 in the ESI†).

Diffusion experiments were conducted to inquire whether complex 2 exists in an oligomeric state in solution (see Section 4 in the ESI†). The diffusion constant (*D* = 6.4 ± 0.2 × 10^−10^ m^2^ s^−1^) matched the predicted volume of a monomeric structural model of 2 (*V*_hydrodyn,exp_ = 746 ± 84 Å^3^, *V*_VdW,calc,monomer_ = 555 Å^3^, *V*_VdW,calc,dimer_ = 1105 Å^3^). The slightly larger experimental volume can be explained by additional flexibility due to the rotatability of the PPh_3_ group, stronger ion pairing of SbF_6_,^32^ or stronger solvent association. In addition, the computational studies combined with 2D NOESY were carried out to get further insight into the detailed structure of the complex (see Section 6 in the ESI†). The Conformer–Rotamer Ensemble Sampling Tool (CREST)^33^ with the xtb1 semi-empirical tight-binding method^34^ was implemented to produce possible conformers and rotamers, which were then used as the inputs for the program DISCON^35^ under the Janocchio^36^ interface. Careful evaluation of the interproton distances within complex 2 was obtained employing a set of 2D z-filtered NOESY spectra with increasing mixing time (see Section 6 in the ESI†). The calculated and experimental inter-proton distances are summarized and compared in Table 1. The obtained values are mostly within the experimental error.^37^

**Table 1:** Comparison of experimental and computational interproton distances *R*_*i*,*j*_ as calculated by Janocchio from the DISCON derived ensemble. Some distances were omitted as they either gave no additional information (*e.g.*, short-range *r*_18#,19#_) or were less reliable due to severe overlap in the 2D NOESY spectra

*i*	*j*	*R*_*i*,*j*,calc_/Å	*r*_*i*,*j*,exp_/Å	|Δ*r*|/Å
15	18#,18′#	4.97	5.17	0.20
13#	18#,18′#	3.85	4.04	0.19
2#	18#,18′#	3.60	3.27	0.33
14	18#,18′#	3.29	3.50	0.21
11	19#,19′#	4.92	4.28	0.64
11	18#,18′#	3.54	3.24	0.30
14	19#,19′#	4.18	3.81	0.37
15	19#,19′#	5.73	5.13	0.60
2#	13#	2.70	2.46	0.24
11	14	5.14	4.86	0.28
12	8	2.63	2.67	0.04
8	11	4.65	4.42	0.23

**Fig. 8:**
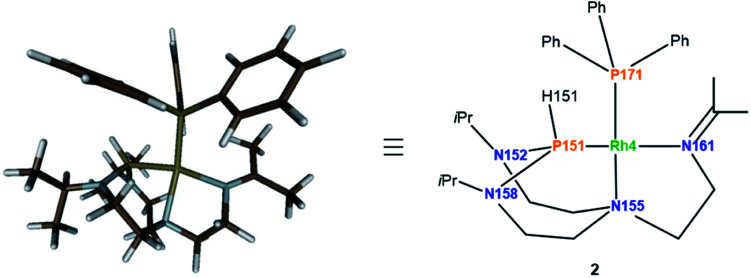
Computed structure of complex 2 at the D3BJ-PBE/def2-SVP level of theory (left) and the associated drawing with labels (right). SbF_6_ anion is left out for clarity. Selected interatomic distances (Å) and angles (°): Rh4–P151 2.207, Rh4–P171 2.218, Rh4–N155 2.247, Rh4–N161 2.109, N158–P151–Rh4 106.14, N152–P151–Rh4 107.76, N158–P151–N152 109.91.

Considering the monomeric molecule suggested by the DOSY experiments, together with the comparison of the interatomic distances of the computed rotamers with the ones of complex 2, enables us to propose the structure of complex 2 as shown in Fig. 8.

**Fig. 9:**
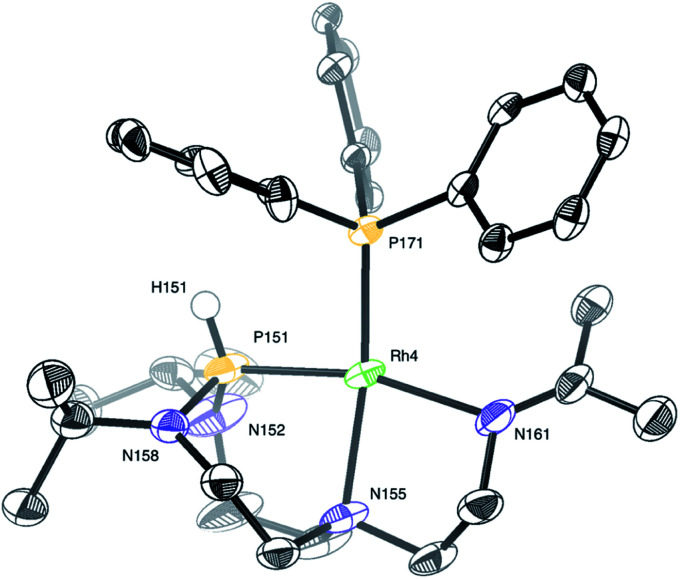
Molecular structure of complex 2 in the solid state. The SbF_6_ anion and protons were removed for clarity, except for the proton on phosphorus. Selected interatomic distances (Å) and angles (°): Rh4–P151 2.162(2), Rh4–P171 2.2158(18), Rh4–N155 2.191(6), Rh4–N161 2.137(6), N158–P151–Rh4 107.4(2), N152–P151–Rh4 109.1(3), N158–P151–N152 109.6(4).

In turn, single crystals of 2 could be obtained by slow evaporation of a diluted solution of the complex solubilized in a dichloromethane : diethyl ether (1 : 4) mixture of solvents. Only one complex of the four independent molecules present in the asymmetric unit is shown for clarity (Fig. 9). The rhodium(i) center displays a square planar geometry with a P–N–N tridentate and triphenylphosphine coordination. X-ray diffraction analysis conclusively shows the shift of the N_ax_ atom to the rhodium center, with the soft monodentate triphenylphosphine ligand promoting a change of the T^i^PrAP coordination mode from bidentate to tridentate. The shifted coordination of N155 atom to rhodium is evidenced by the formation of the Rh4–N155 bond (2.191(6) Å). This confirms the observations made in ^1^H–^31^P HMBC, with the protons close to N155 having strong correlations with the phosphorus of triphenylphosphine (see Fig. S25 in the ESI†). Besides, the angles around the P151 atom, ranging from 107.4(2)° to 109.6(4)°, suggest a tetrahedral structure, which is in good agreement with the larger P–H coupling constant of 2 compared to complex 1. Other structural parameters provided by X-ray diffraction analysis of 2 are compared to the computed data and summarized in Table 2. Altogether, it transpires that the combination of NMR experiments and DFT calculations provides an accurate molecular description of the complex.

**Table 2:** Comparison of computationally and experimentally determined interatomic distances (Å) and angles (°) for complex 2

Selected interatomic distances	*R*_calc_ (Å)	*r*_exp_ (Å)	|Δ*r*| (Å)	Selected angles	∠_calc_ (°)	∠_exp_ (°)	|Δ∠| (°)
Rh4–P151	2.207	2.162(2)	0.045	N158–P151–Rh4	106.14	107.4(2)	1.3
Rh4–P171	2.218	2.2158(18)	0.002	N152–P151–Rh4	107.76	109.1(3)	1.3
Rh4–N155	2.247	2.191(6)	0.056	N158–P151–N152	109.91	109.6(4)	0.3
Rh4–N161	2.109	2.137(6)	0.028				

**Scheme 3:**
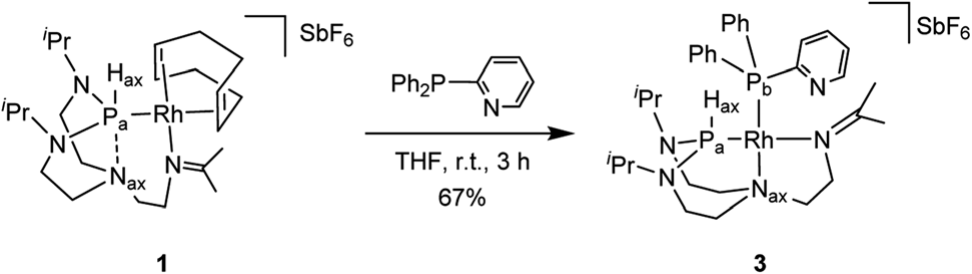
Preparation of complex 3.

Combining hard and soft donor atoms into a ligand environment can be used as a proxy to control a given complex's thermodynamic and kinetic stability. Thus, we considered 2-pyridyldiphenylphosphine as another ancillary ligand and investigated the effect of an additional hard pyridyl donor on the coordination chemistry and thermodynamic properties of the resulting complex. The reaction of complex 1 with 2-pyridyldiphenylphosphine in tetrahydrofuran afforded complex 3 (Scheme 3). The ^31^P{^1^H} NMR spectrum shows two doublet of doublets with the constants (^1^*J*_P_a_,P_b__ = 60 Hz, ^1^*J*_P_a_,Rh_ = 161 Hz, ^1^*J*_P_b_,Rh_ = 204 Hz). The value of the ^1^*J*_P_a_,P_b__ coupling constant reveals a *cis* conformation to each other. The ^1^*J*_P_a___,__H_ax__ coupling increases from 331 Hz (complex 1) to 426 Hz (complex 3), indicating a higher s-character of the P–H bond. The ^13^C{^1^H} NMR spectrum reveals that the imine group coordinated to the rhodium center has a coupling constant (^2^*J*_C,Rh_ = 1.6 Hz) similar to the one of complex 2 (^2^*J*_C,Rh_ = 1.6 Hz). The correlation between the H_ax_ and the N_ax_ atoms present in complex 1 is absent in the ^1^H–^15^N HMBC spectrum (see Fig. S39 in the ESI†). The diffusion constant of 3 (*D* = 6.33 ± 0.08 × 10^−10^ m^2^ s^−1^) is comparable to 2 (*D* = 6.4 ± 0.2 × 10^−10^ m^2^ s^−1^). Besides, the ^1^H NMR spectrum of complex 3 shows considerable similarities with complex 2. Based on these observations, it can be reasonably assumed that the two complexes feature an analogous molecular structure.

**Scheme 4:**
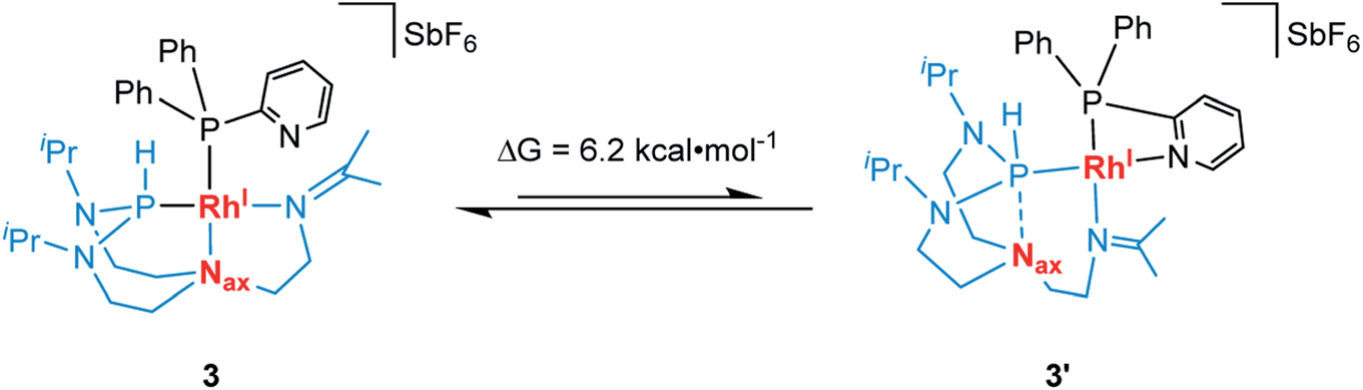
Suggested dynamic equilibrium between complex 3 and 3′ and the associated variation in free energy.

We then hypothesized whether, in contrast to complex 2, the pyridyl donor in complex 3 might displace the imine at the rhodium center resulting in its bidentate chelation to the metal center.^38^ This, in turn, may cause the T^i^PrAP ligand backbone to further rearrange its coordination mode from tridentate to bidentate and generate a complex similar to 3′. Therefore, computational calculations at the ωB97X-D3BJ/def2-TZVPP level of theory were carried out (see Section 7 in the ESI†).^39^ Theory suggests that the pyridine coordinated complex 3′ is higher in energy by only Δ*G* = 6.2 kcal mol^−1^ when compared to complex 3 (Scheme 4). It might thus constitute a relevant intermediate at elevated temperatures. Attempts to structurally characterize 3′ have, however, been unsuccessful at this stage. In terms of perspectives, introducing a spatial distance between the pyridine and phosphorus atom might reduce the ring strain and enhance the structural flexibility of the resulting complex. This might shift the equilibrium towards the formation of a complex comparable to 3′ by enhancing its intrinsic stability.

The propensity of the T^i^PrAP ligand backbone to purposely adapt itself and accommodate different geometries and coordination modes around the metal center offers strategies for controlling the coordination environment at the metal center and subsequent activity of the entire molecular edifice.

## Conclusion

In summary, the synthesis and coordination chemistry of rhodium(i) complexes with a tris(isopropyl)-azaphosphatrane (T^i^PrAP) ligand architecture are reported. The molecular structure of complex 1 indicated the insertion of the metal center into one of the P–N bonds of the ligand and proton transfer from a methine group to the phosphorus atom. A transannular interaction was confirmed by NMR spectroscopy, single-crystal X-ray diffraction study, and NBO analysis. The addition of lithium chloride resulted in the altered dynamic behavior of complex 1 in solution, which was studied by NMR spectroscopy. Substituting the cyclooctadiene ligand at the metal center with triphenylphosphine or 2-pyridyldiphenylphosphine unveiled the adaptive nature of the T^i^PrAP backbone. This adaptability resides in the axial nitrogen, which can shift from an interaction with the phosphorus atom to the coordination of the rhodium center. The ligand architecture is therefore capable of binding the rhodium center both in a bidentate or tridentate manner. This reversible and apparent hemilability demonstrates that the T^i^PrAP ligand framework can accommodate electronic or steric changes around the rhodium center by opening or closing a coordination site at the metal center. Altogether, our study shows that precise molecular control of the metal center's immediate environment can develop. Application of this peculiarity in controlled bond activation reactions is currently underway.

## Conflicts of interest

There are no conflicts to declare.

## Supplementary Material

RA-011-D1RA07126B-s001

RA-011-D1RA07126B-s002
