# Supplementary material for: Rhodium(i) complexes derived from tris(isopropyl)-azaphosphatrane—controlling the metal–ligand interplay
Source: RSC Adv. 2021 Nov 22;11(59):37383–91. doi: 10.1039/d1ra07126b (PMC9043836; doi:10.1039/d1ra07126b)
Supplement: RA-011-D1RA07126B-s001 [file RA-011-D1RA07126B-s001.pdf]

Electronic Supplementary Material (ESI) for RSC Advances.
This journal is © The Royal Society of Chemistry 2021

Supporting Information

Rhodium(I) Complexes Derived from Tris(isopropyl)-azaphosphatrane—Controlling the Metal-Ligand Interplay

Wei-Chieh Chang,[1,2,#] Fritz Deufel,[3,4,#] Thomas Weyhermüller,[1] Christophe Farès[3] and Christophe Werlé[1,2,*]

[1] Max Planck Institute for Chemical Energy Conversion, Stiftstr. 34 – 36, 45470 Mülheim an der Ruhr, Germany.

[2] Ruhr University Bochum, Universitätsstr. 150, 44801 Bochum, Germany.

[3] Max-Planck-Institut für Kohlenforschung, Kaiser-Wilhelm-Platz 1, D-45470 Mülheim an der Ruhr, Germany.

[4] Current Address: Organisch-Chemisches Institut, Corrensstraße 36, D-48149 Münster, Germany.

[#] These authors contributed equally to this work.

[*] Email: christophe.werle@cec.mpg.de

Contents

1. General method S2
2. Synthesis of the complexes S3
3. NMR spectra S6
   Complex **1** S6
   Complex **2** S14
   Complex **3** S22
4. Diffusion measurement S29
5. Kinetic studies S34
6. Quantitative NOESY S43
7. Computational details S49
   Geometries S53
   Complex **1** (crystal structure) S53
   Complex **1** (optimized structure) S55
   Complex **2** S58
   Complex **3** S61
   Complex **3’** S65
8. X-ray diffraction studies S69
9. References S71

1. General method

All the reactions were prepared inside an MBraun glovebox under an argon atmosphere. Solvents for air- and moisture-sensitive experiments were purified using a two-column solvent purification system (MBraun-SPS-7), transferred directly to the glovebox, and stored over molecular sieves (3 Å). Tris(isopropyl)-azaphosphatrane (T^{i}PrAP) was prepared according to the corresponding published procedures.[1] $Rh(COD)_2SbF_6$, triphenylphosphine, and 2-pyridyldiphenylphosphine were purchased from abcr and Sigma Aldrich and used without further purification. IR spectra of the samples were recorded on Thermo Scientific™ Nicolet™ iS™5 FT-IR Spectrometer with iD7 ATR Accessory. HR-MS spectra were recorded on a Bruker ESQ3000 spectrometer. Elemental analyses (carbon, hydrogen, and nitrogen) were carried out using an Elementar UNICUBE elemental analyzer. NMR spectra were recorded using the following Bruker spectrometer: 400 MHz Avance III (PA BBO 400SB BB-H-D-05 Z), 500 MHz Avance III (PA BBO 500S1 BBF-H-D-05 Z PLUS SP), 600 MHz Avance III* (CP TCI 600S3 H-C/N-D-05 Z) or 600 MHz Bruker Avance Neo* (CP2.1 BBO 600S3 BB-H&F-D-05 Z XT). Some measurements were carried out exclusively at specific instruments due to probe specifications or beneficial sensitivity gains due to, e.g., *cryogenic probes: ^{1}H-^{15}N-HMBC: 600 MHz Avance III, Diffusion: 400 MHz Avance III, EXSY build-up: 600 MHz Avance III, 600 MHz Avance Neo, NOESY build-up: 600 MHz Avance III. ^{1}H-^{103}Rh-HMBC: 500 MHz Avance III. ^{103}Rh chemical shifts are given to high frequency from $\Xi(^{103}Rh) = 3.16$ MHz.

2. Synthesis of the complexes

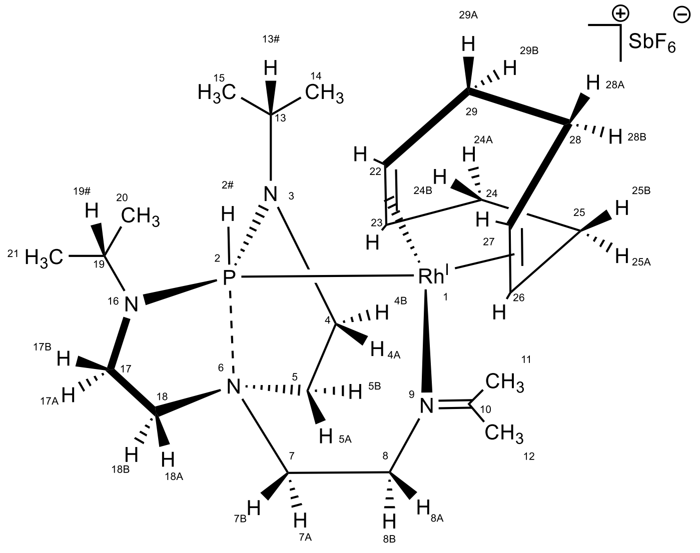

Complex 1. A suspension of $Rh(COD)_2SbF_6$ (0.3353 g, 0.604 mmol) in DCM (4 mL) containing 4 Å molecular sieves was stirred for 30 mins at room temperature. After that time, a solution of T^{i}PrAP (0.1815 g, 0.604 mmol) in DCM (4 mL) was added to the aforementioned solution and stirred for 1 hour. The reaction mixture was filtered and the filtrate was concentrated. The residue was washed with diethyl ether. The crude product was dissolved in DCM and layered with diethyl ether at -20°C for 1-2 days to afford yellow solid (0.3311g, 73% yield). **^{1}H NMR (500.1 Hz, THF-d$_8$)**: δ 6.46 (d, *J* = 331.1 Hz, 1H, $H_{2\#}$), 5.02-4.90 (m, 1H, H_{26}), 4.49-4.37 (m, 1H, H_{27}), 4.17 (ddd, *J* = 13.0, 11.3, 4.6 Hz, 1H, H_{7A}), 3.94-3.84 (m, 2H, H_{8A} and H_{22}), 3.78 (tdd, *J* = 12.3, 3.9 and 2.1, 1H,H_{4A}), 3.52 (m, 1H,H_{8B}), 3.46-3.36 (m, 1H, $H_{13\#}$), 3.36-3.27 (m, 2H, $H_{19\#}$ and H_{23}), 3.13 (ddd, *J* = 29.3, 14.1, 4.0 Hz, 1H, H_{4B}), 2.97 (s, 3H, H_{11}), 2.95-2.88 (m, 2H, H_{5A} and H_{7B}), 2.87-2.75 (m, 2H, H_{17A} and H_{18A}), 2.68 (td, *J* = 12.8, 4.0 Hz, 1H, H_{5B}), 2.63-2.55 (m, 3H, H_{18A}, H_{25A}, H_{29A}), 2.46-2.37 (m, 1H, H_{18B}), 2.36-2.26 (m, 2H, H_{24A} and H_{28A}), 2.25-2.17 (m, 2H, H_{29B} and H_{25B}), 2.15 (s, 3H, H_{12}), 2.03-1.90 (m, 2H, H_{24B} and H_{28B}), 1.19 (d, *J* = 6.6 Hz, 3H, H_{15}), 1.10 (, *J* = 6.6 Hz, 3H, H_{21}), 1.06 (d, *J* = 6.7 Hz, 3H, H_{20}), 1.06 (d , *J* = 6.6 Hz, 3H, H_{14}) ppm. **^{13}C{^{1}H} NMR (125.8 Hz, THF-d$_8$)**: δ 178.6 (d, C_{10}), 103.0 (dd, C_{26}), 101.6 (dd, C_{27}), 81.8 (ds, C_{22}), 76.2 (d, C_{23}), 58.66 (d, C_5), 56.1 (d, C_{13}), 54.4 (d, C_{19}), 53.9 (s, C_{18}), 53.9 (s, C_7), 52.2 (s, C_8), 41.9 (d, C_4), 40.7 (d, C_{17}), 34.1 (d, C_{29}), 33.2 (d, C_{11}), 31.1 (d, C_{25}), 30.9 (d, C_{24}), 28.6 (s, C_{28}), 22.8 (s, C_{20}), 22.8 (d, C_{21}), 22.5 (d, C_{14}), 22.2 (s, C_{12}), 21.2 (s, C_{15}), 20.8 (s, C_{20}) ppm. **^{31}P{^{1}H} NMR (202.4 Hz, THF-d$_8$)**: δ 68.5 (d, $J_{P\text{-}Rh}$ = 201.5 Hz) ppm. **^{31}P NMR (202.42 Hz, THF-d$_8$)**: δ 68.5 (dd, $J_{P\text{-}H,\,P\text{-}Rh}$ = 331.4, 201.5 Hz) ppm. **ATR-IR**: 2974, 2959, 2927, 2883, 2835, 2208, 1648 cm^{-1}. **HRMS (ESI)**: calcd. for $[C_{23}H_{45}N_4P_1Rh_1]^+$: 511.24314; found 511.24347. **Anal.** Calcd for $C_{23}H_{45}N_4P_1Rh_1Sb_1F_6$: C, 36.97; H, 6.07; N, 7.50. Found: C, 37.36; H, 6.21; N, 7.60.

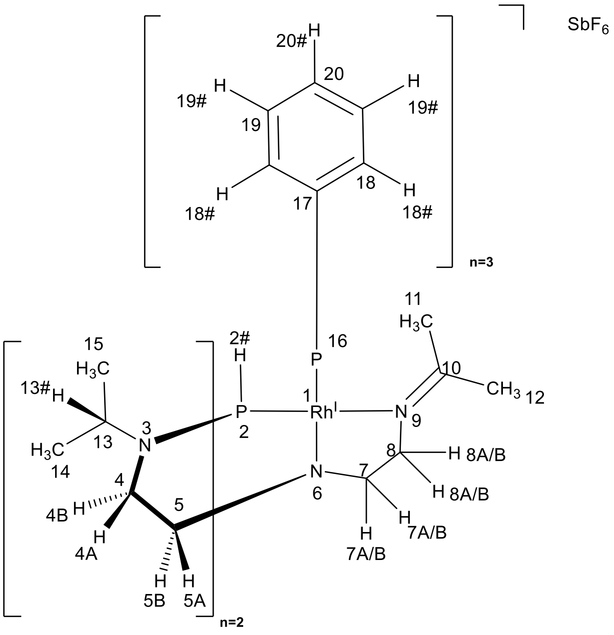

Complex 2. A mixture of complex **1** (0.1128 g, 0.151 mmol) and triphenylphosphine (0.0396 g, 0.151 mmol) was dissolved in dichloromethane (4 mL) inside a pressure tube. The resulting mixture was then stirred for 90 minutes at 70 °C. After that time, the mixture was concentrated and washed with diethyl ether leading to a gel like compound. Drying the residue under vacuum afforded complex **2** as a yellow solid (0.0816 g, 60% yield). **^{1}H NMR (500.1 Hz, THF-d$_8$)**: δ 7.6 (m, 2H, $H_{18\#}$), 7.39(m, $C_{20\#}$), 7.37 (m, 2H, $H_{19\#}$), 5.61 (ddd, $J_{H\text{-}P2,H\text{-}Rh,\ H\text{-}P16}$ = 413.4, 11.8, 6.9 Hz, 1H, $H_{2\#}$), 3.93 (m, 2H, H_{4A}), 3.91 (m, 2H, H_{8AB}), 3.27 (m, 2H, H_{4B}), 3.13 (m, 2H, H_{5A}), 3.07 (m, 2H, H_{5B}), 3.05 (m, 2H, $H_{13\#}$), 2.94 (m, 2H, H_{7AB}), 1.83 (s, 3H, H_{12}), 1.51 (s, 3H, H_{11}) 1.15 (d, $J_{H\text{-}H}$ = 6.7Hz, 6H, C_{14}), 1.06 (d, $J_{H\text{-}H}$ = 6.6Hz, 6H, C_{15}) ppm. **^{13}C{^{1}H} NMR (125.8 Hz, THF-d$_8$)**: δ 178.6 (d, C_{10}), 137.0 (d, C_{17}), 134.5 (d, C_{18}), 130.5 (d, C_{20}), 129.2 (d, C_{19}), 69.7 (s, C_7), 65.1 (d, C_5), 54.9 (s, C_8), 51.9 (d, C_{13}), 43.9 (d, C_4), 32.3 (d, C_{11}), 23.1 (d, C_{14}), 21.8 (d, C_{15}), 20.0 (d, C_{12}) ppm. ^{31}P{^{1}H} NMR (202.4 Hz, THF-d$_8$): δ 119.7 (dd, $J_{P2\text{-}Rh,\ P\text{-}P}$ = 163.8, 59.9 Hz, P_2), 50.7 (dd, $J_{P\text{-}Rh,\ P16\text{-}P2}$ = 202.8, 59.8 Hz, P_{16}) ppm. **^{31}PNMR (202.4 Hz, THF-d$_8$)**: δ 119.7 (dd, $J_{P\text{-}H,\ P\text{-}Rh,\ P\text{-}P}$ = J = 413.4, 163.8, 59.9 Hz), 50.7 (dd, $J_{P\text{-}Rh,\ P16\text{-}P2}$ = 202.8, 59.8 Hz, P_{16}) ppm. **^{103}Rh NMR (15.8 Hz, THF-d$_8$)**: δ -8430 ppm. **ATR-IR**: 3070, 3053, 2964, 2932, 2874, 2305, 1647 cm^{-1}. **HRMS (ESI)**: calcd. for $[C_{33}H_{48}N_4P_2Rh_1]^+$: 665.24038; found 665.24032.

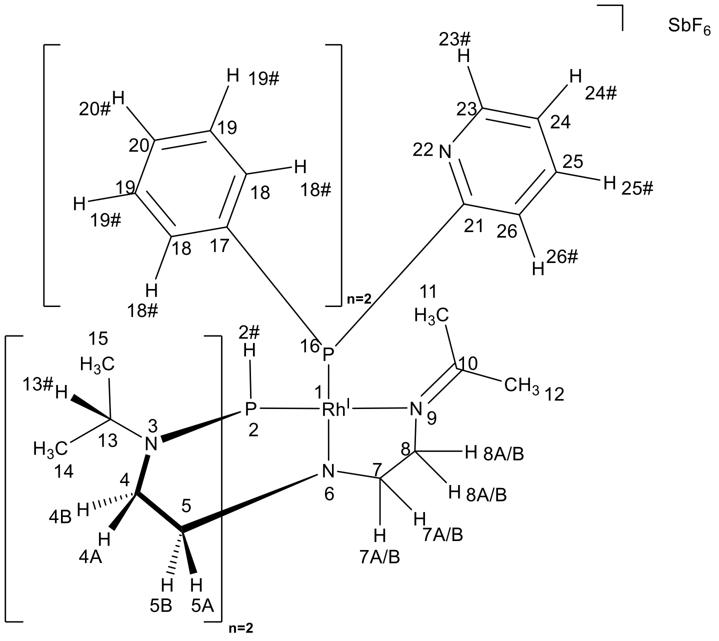

Complex 3. A mixture of complex **1** (0.0769 g, 0.010 mmol) and 2-pyridyldiphenylphosphine (0.0298 g, 0.11 mmol) was dissolved in THF (3 mL) and stirred during 3 hours at room temperature. After that time, the mixture was concentrated and washed with diethyl ether (3×1 mL). Further purification was accomplished by layering a dichloromethane solution of the obtained complex with pentane. This led to the precipitation of a gel-like material which afforded complex **3** as a yellow solid upon drying under vacuum (0.0626 g, 67% yield). **^{1}H NMR (500.1 Hz, THF-d$_8$)**: δ 8.65, (d, $J_{H23\text{-}H24}$= 4.5 Hz, 1H, H_{23}), 7.74 (m, 4H, H_{18}), 7.69 (m, 1H, H_{25}), 7.59 (m, 1H, H_{26}), 7.39 (m, 2H, H_{20}), 7.36 (m, 4H, H_{19}), 7.27 (m, 1H, H_{24}), 5.61 (ddd, $J_{H\text{-}P2,H\text{-}Rh,\ H\text{-}P16}$ = 426.0, 11.3, 6.9 Hz, 1H, $H_{2\#}$), 3.92 (m, 2H, H_{4A}), 3.90 (m, 2H, H_{8A} and H_{8B}), 3.24 (m, 2H, H_{4B}), 3.12 (m, 2H, H_{5A}), 3.11 (m, 2H, $H_{13\#}$), 3.06 (m, 2H, H_{5B}), 2.94 (m, 2H, H_{7A} and H_{7B}), 1.82, (s, 3H, H_{12}), 1.55 (s, 3H, H_{11}), 1.13 (d, $J_{H14\text{-}H13\#}$ = 6.6 Hz, 6H, H_{14}), 1.07 (d, $J_{H15\text{-}H13\#}$ = 6.6 Hz, 6H, H_{15}) ppm. **^{13}C{^{1}H} NMR (125.8 Hz, THF-d$_8$)**: δ 178.0 (d, C_{10}), 161.77 (d, C_{21}), 150.6 (d,C_{23}), 136.5 (d, C_{25}), 136.3 (d, C_{17}), 135.2 (d, C_{18}), 130.6 (d, C_{20}), 129.0 (d, C_{26}), 128.7 (d, C_{19}), 124.0 (d, C_{24}), 69.2 (s, C_7), 65.1 (d, C_5), 54.5 (s, C_8), 51.8 (s, C_{13}), 44.0 (d, C_4), 32.5 (d, C_{11}), 23.2 (d, C_{14}), 21.8 (d, C_{15}), 20.0 (d, C_{12}) ppm. **^{31}P{^{1}H} NMR (202.4 Hz, THF-d$_8$):** δ 119.7 (dd, $J_{P2\text{-}Rh,\ P\text{-}P}$ = 163.8, 59.9 Hz, P_2), 51.4 (dd, $J_{P\text{-}Rh,\ P16\text{-}P2}$ = 203.7, 60.1 Hz, P_{16}) ppm. ^{31}PNMR (202.4 Hz, THF-d$_8$): δ 119.7 (dd, $J_{P\text{-}H,\ P\text{-}Rh,\ P\text{-}P}$ = J = 413.4, 163.8, 59.9 Hz), 51.4 (dd, $J_{P\text{-}Rh,\ P16\text{-}P2}$ = 203.7, 60.1 Hz, P_{16}) ppm. **ATR-IR**: 3052, 2963, 2935, 2873, 2315, 1648 cm^{-1}. **HRMS (ESI)**: calcd. for $[C_{32}H_{47}N_5P_2Rh_1O_2]^+$: 698.22545; found 698.22466. **Anal.** Calcd for $C_{32}H_{47}N_5P_2Rh_1Sb_1F_6$ • 0.5 CH_2Cl_2: C, 41.32; H, 5.12; N, 7.41. Found: C, 41.52; H, 5.22; N, 7.03.

3. NMR spectra

Complex 1

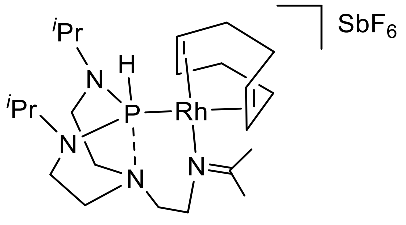

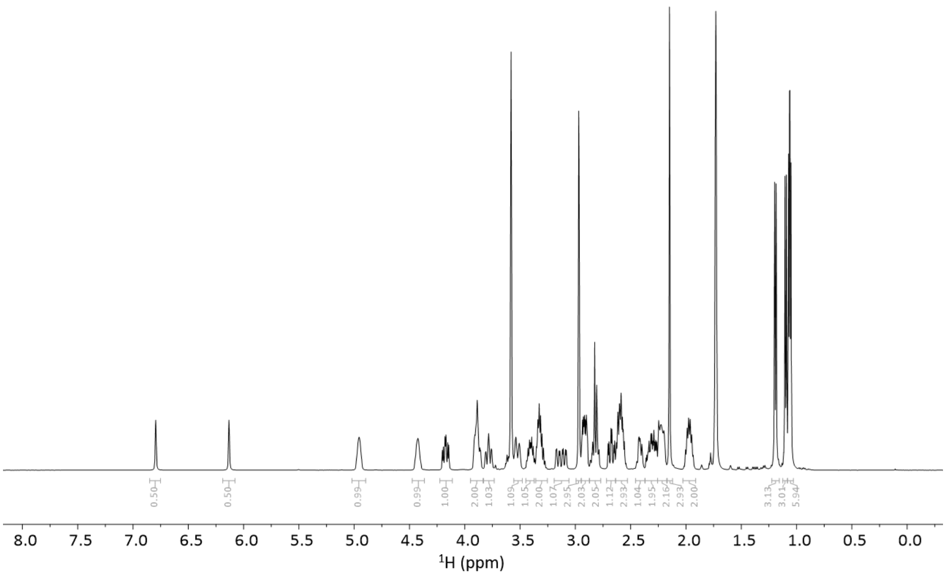

Figure S1 – **^{1}H NMR (500.1 MHz, THF-d$_8$)** spectrum of complex **1**.

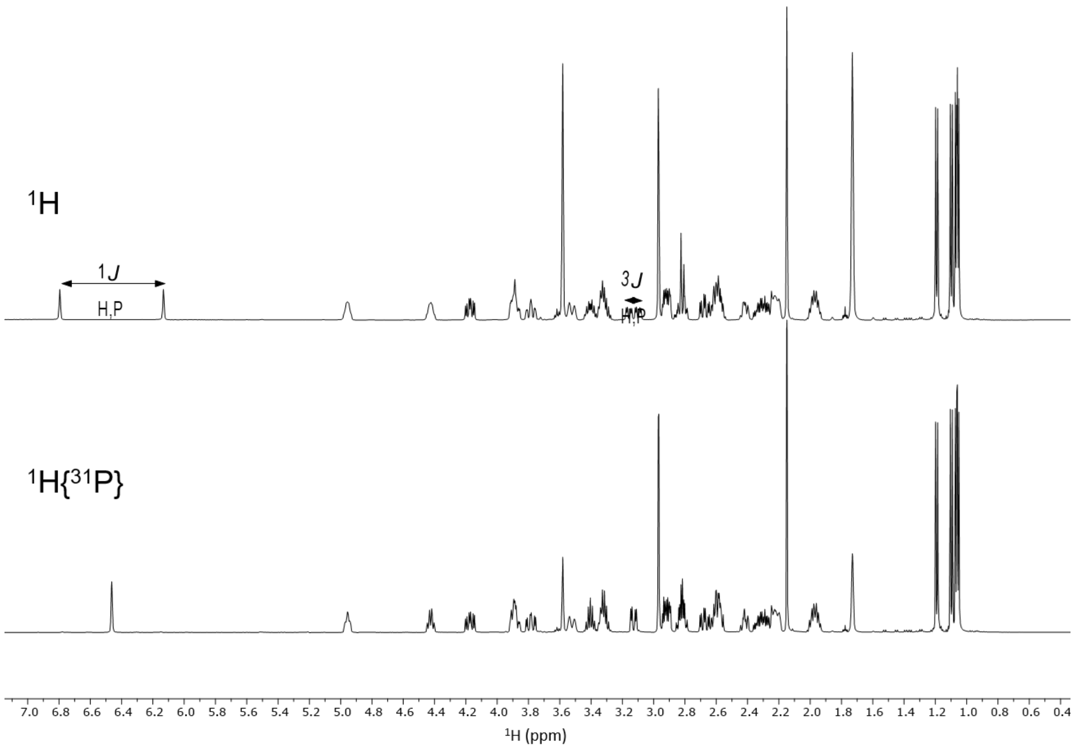

Figure S2 – Comparison of **^{1}H** and **^{1}H{^{31}P} (500.1 MHz, THF-d$_8$)** spectra of complex **1**.

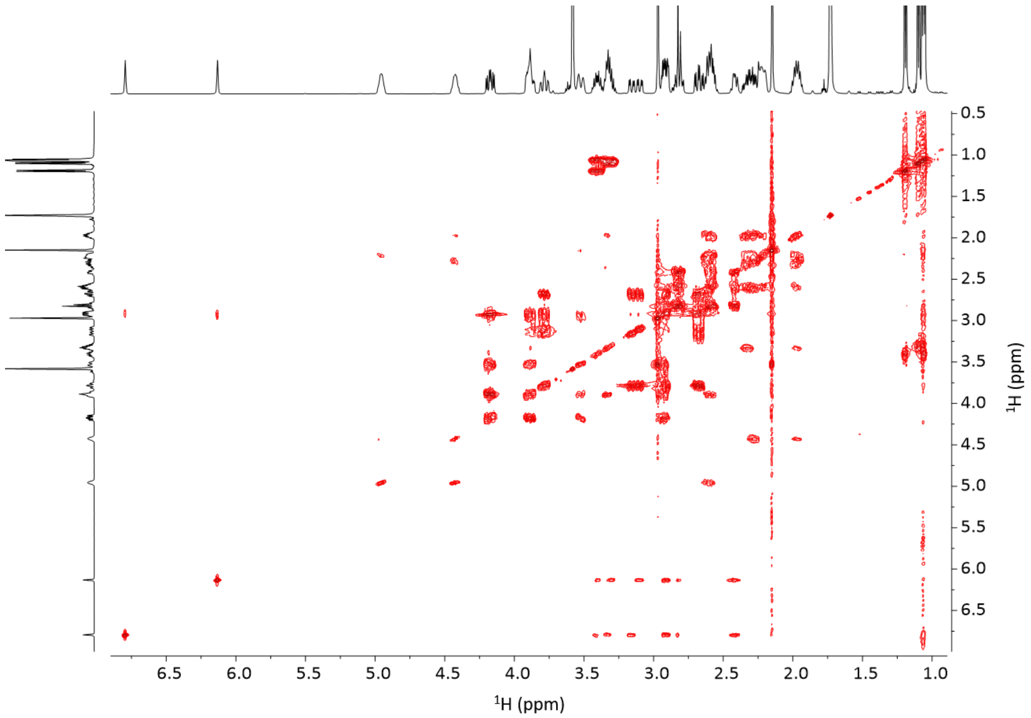

Figure S3 – ^{1}H,^{1}H-COSY (cosygpppqf) (499.9 MHz, THF-d$_8$) spectrum of complex **1**.

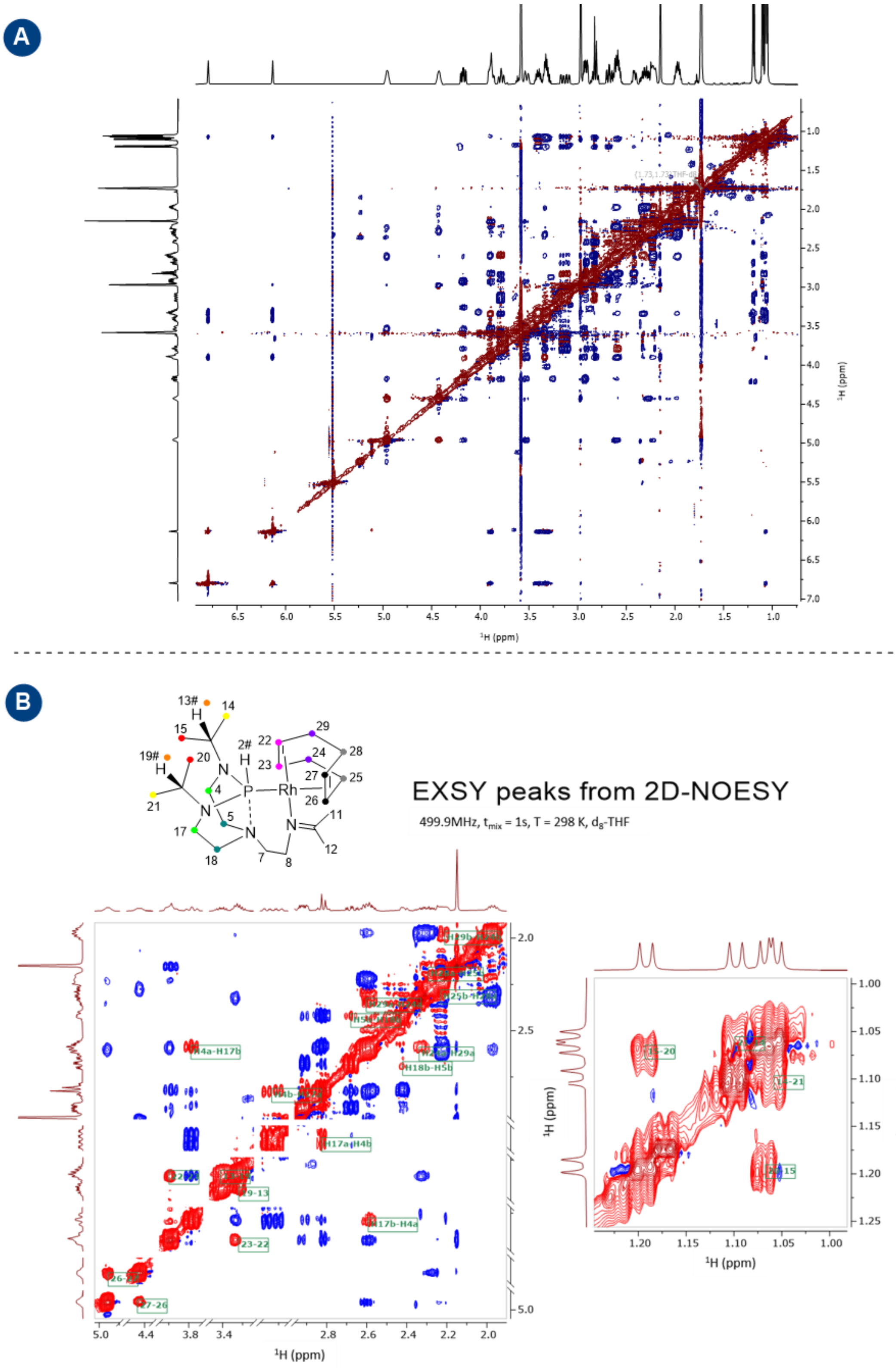

Figure S4 – Panel A: **^{1}H,^{1}H-NOESY (noesygpph) (499.9 MHz, THF-d$_8$)** spectrum of complex **1**; panel B: enlarged **^{1}H,^{1}H-NOESY** peaks of complex **1**.

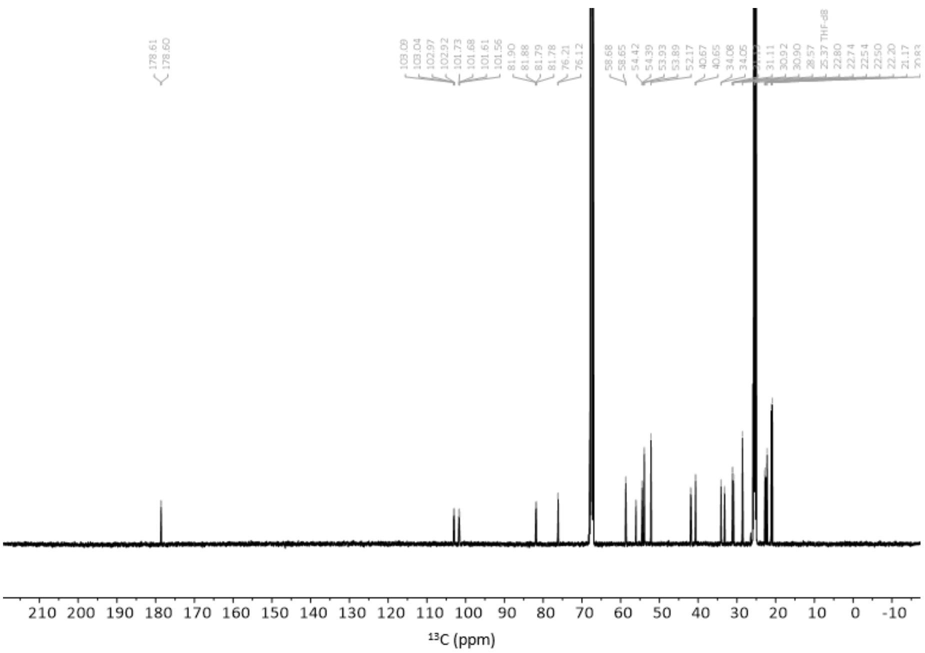

Figure S5 – $^{13}C\{^{1}H\}$ NMR (125.8 MHz, THF-d_8) spectrum of complex **1**.

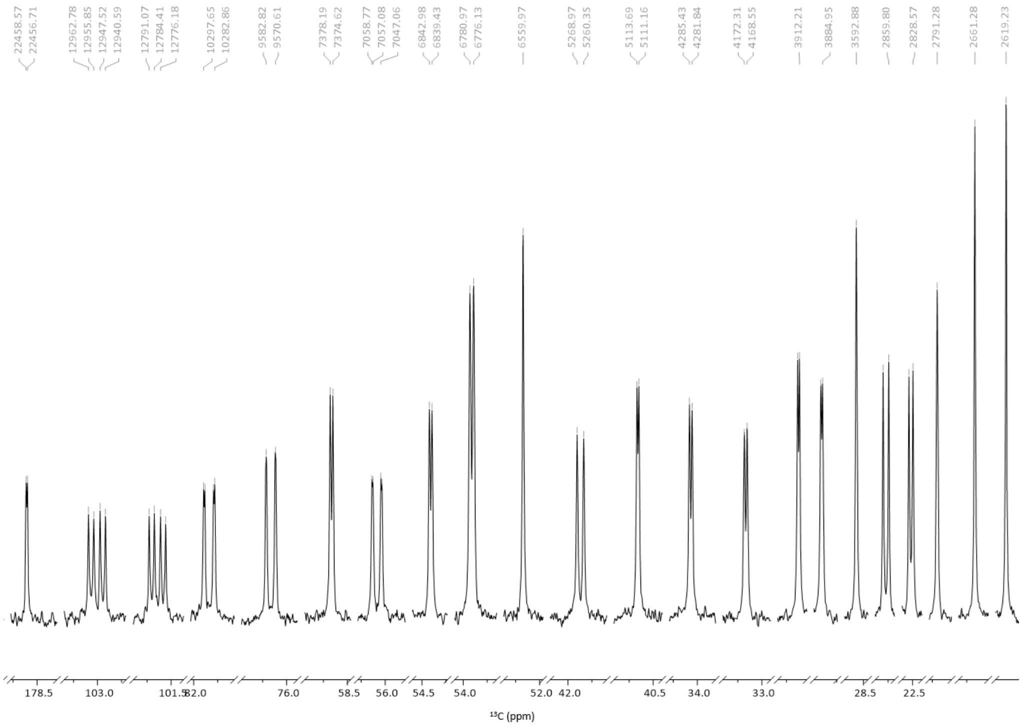

Figure S6 – Enlarged **$^{13}C\{^{1}H\}$ NMR (125.8 MHz, THF-d_8)** peaks of complex **1**.

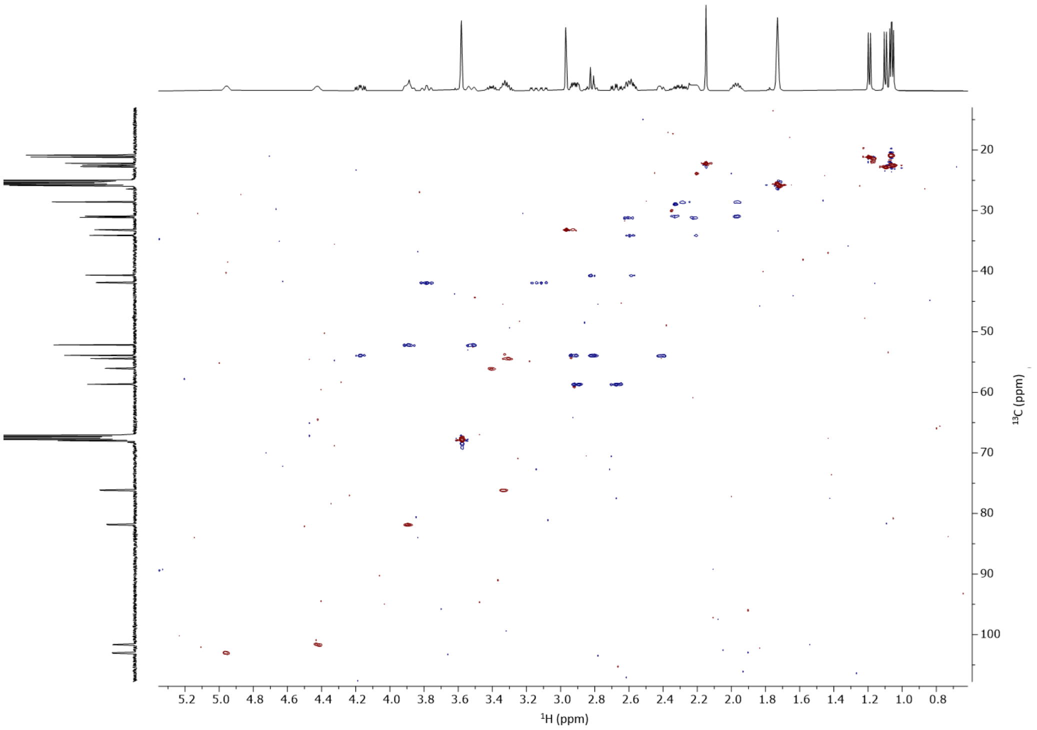

Figure S7 – Multiplicity edited **^{1}H-^{13}C HSQC (hsqcedetgp) (499.9, 125.70 MHz, THF-d$_8$)** experiment of complex **1**.

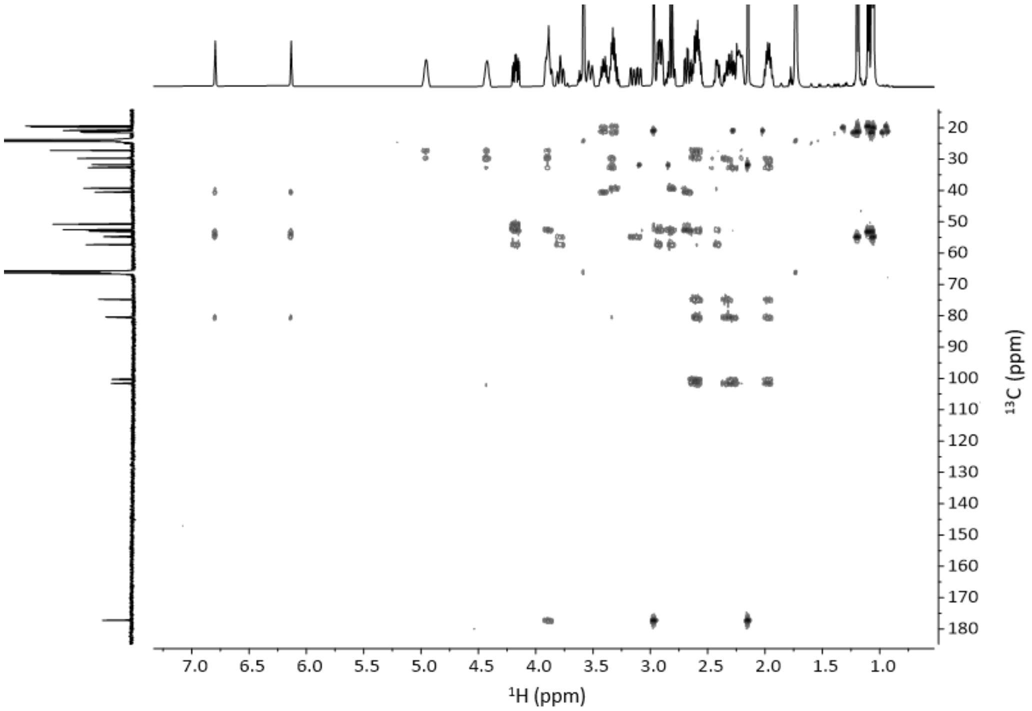

Figure S8 – ^{1}H-^{13}C HMBC (hmbcgplpndqf) (499.9, 125.70 MHz, THF-d$_8$) experiment of complex **1**.

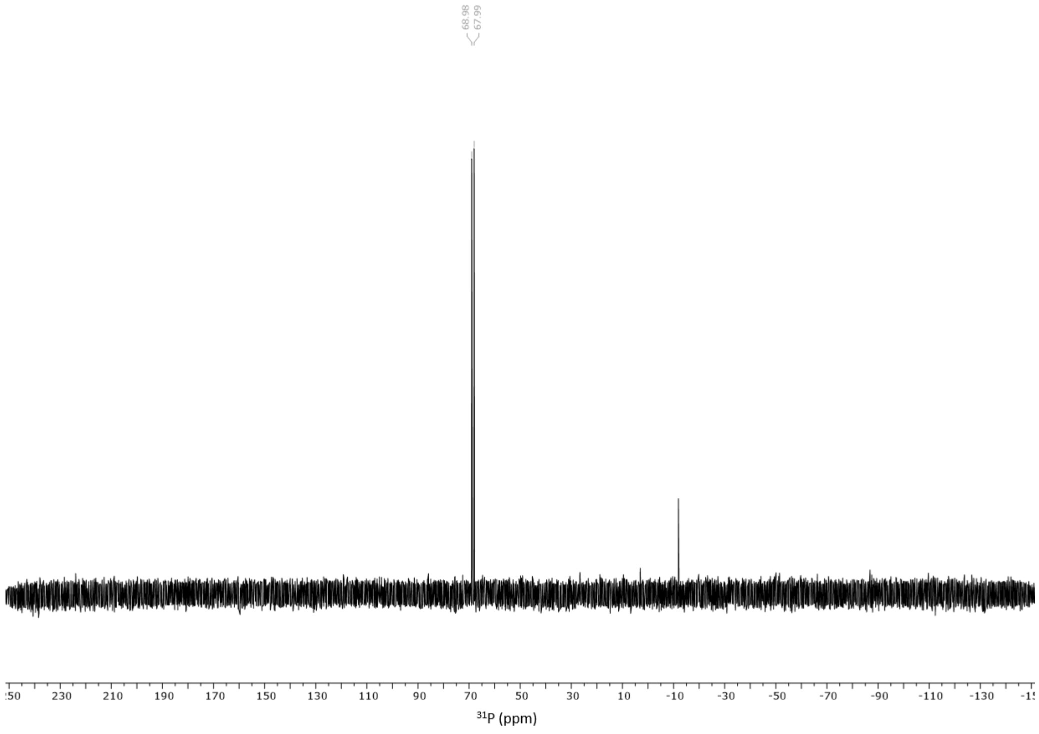

Figure S9 – Overview $^{31}P\{^{1}H\}$ (202.4 MHz, THF-d_8) spectrum of complex **1**.

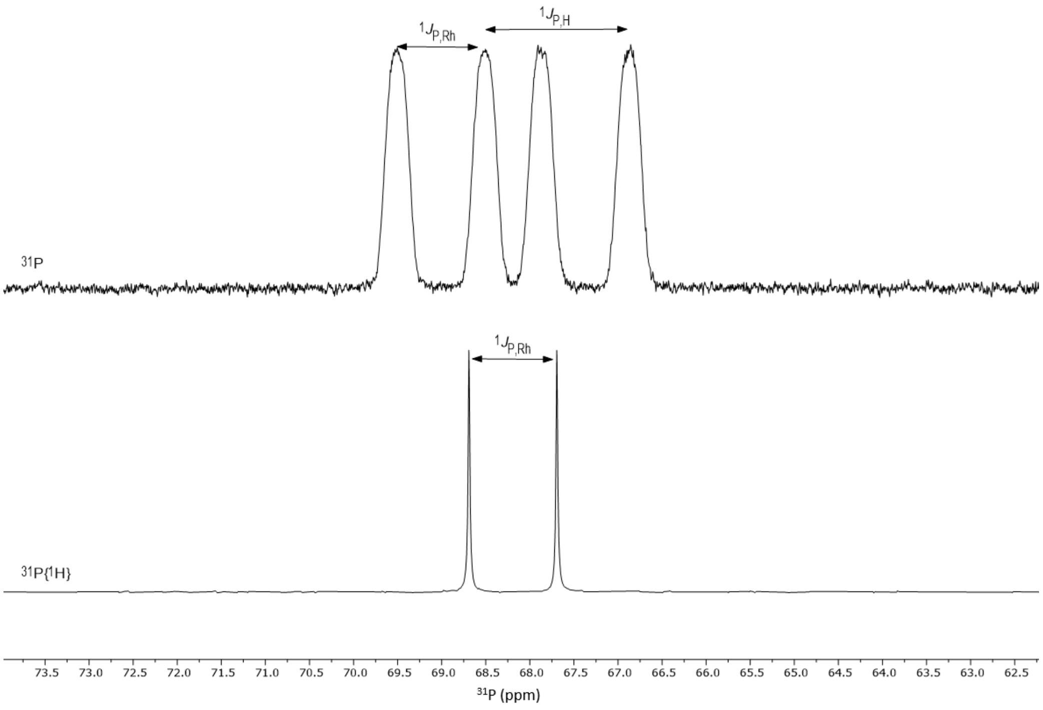

Figure S10 – Comparison of **^{31}P** and **$^{31}P\{^{1}H\}$ (202.4 MHz, THF-d_8)** spectra of complex **1**.

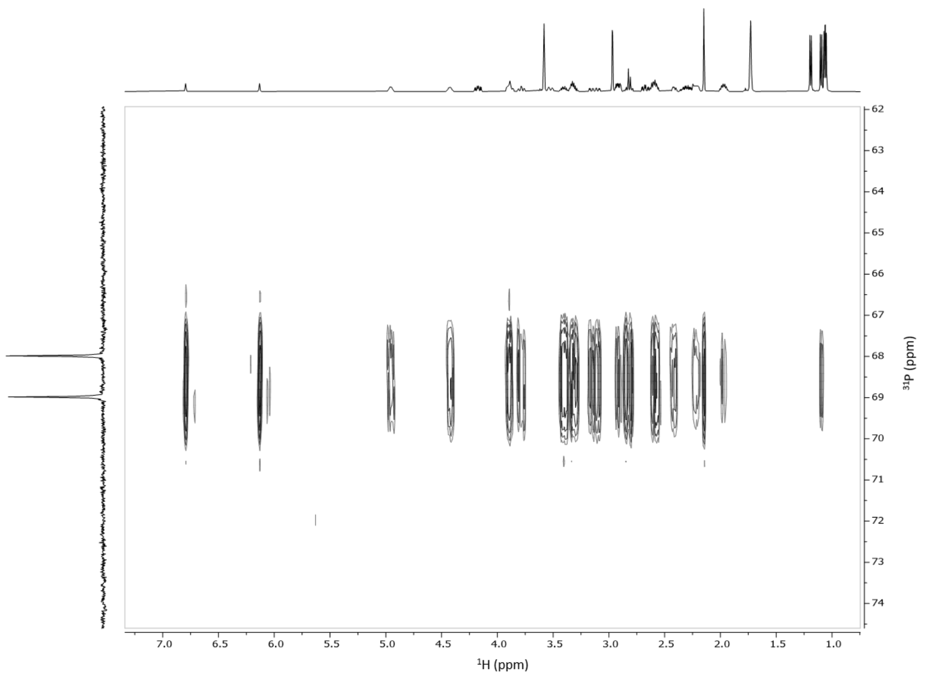

Figure S11 – ^{1}H-^{31}P HMBC (hmbcgpndqf) (499.9, 202.4 MHz, THF-d$_8$) experiment of complex **1**.

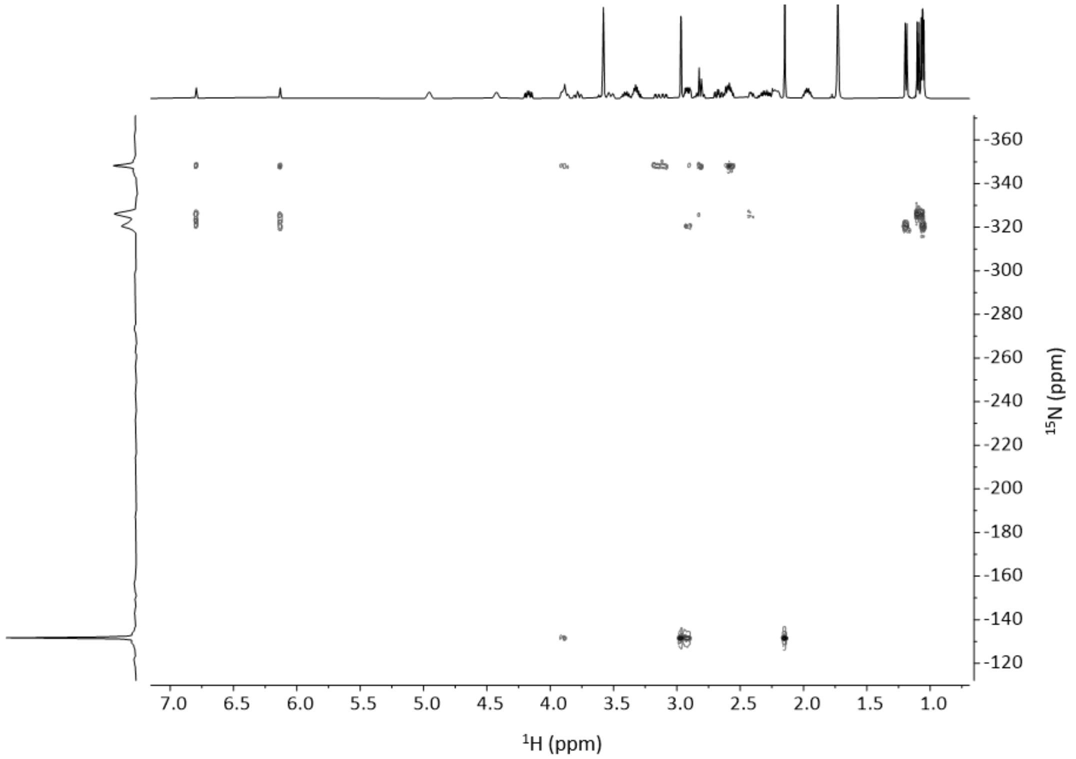

Figure S12 – ^{1}H-^{15}N HMBC (hmbcgpndqf) (500.1, 50.7 MHz, THF-d$_8$) experiment of complex **1**.

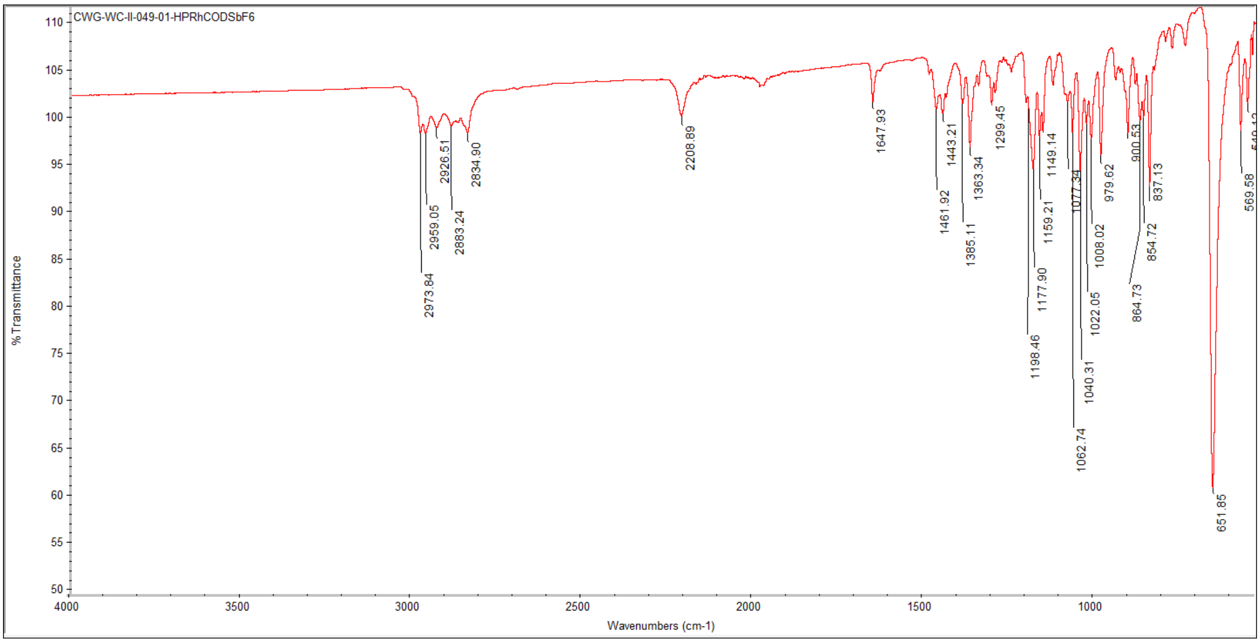

Figure S13 – ATR-IR spectrum of complex **1**.

Complex 2

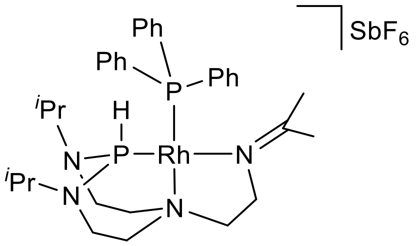

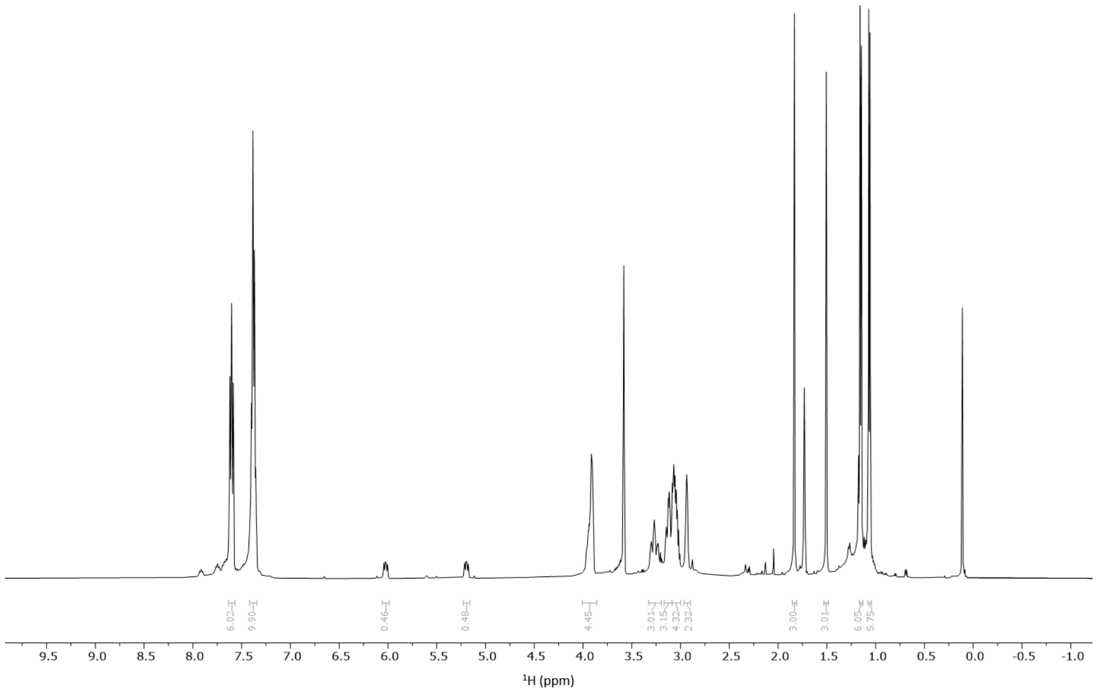

Figure S14 – ^{1}H NMR (500.1 MHz, THF-d_8) spectrum of complex **2**.

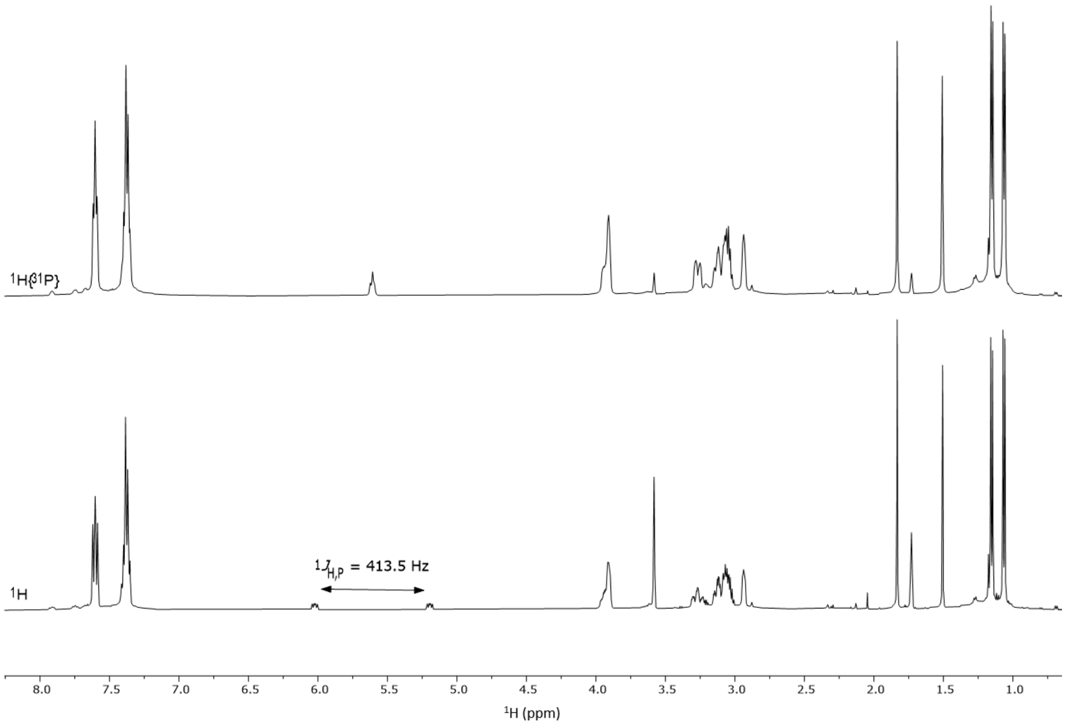

Figure S15 – Comparison of **^{1}H** and **^{1}H{^{31}P} (500.1 MHz, THF-d$_8$)** spectra of complex **2**.

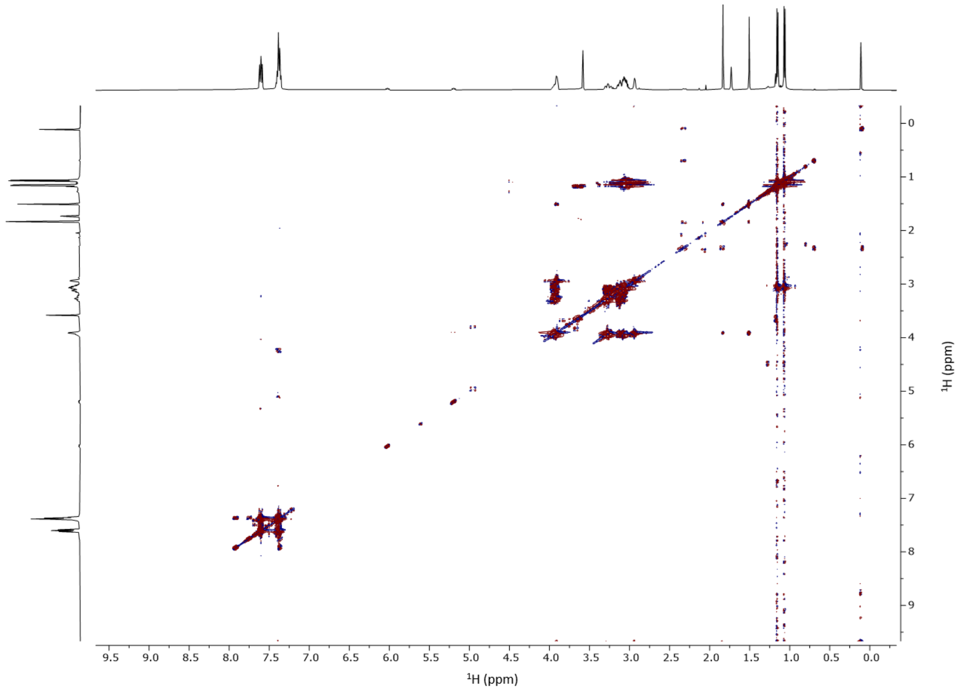

Figure S16 – DQF ^{1}H,^{1}H-COSY (cosygpmfphpp) (499.9 MHz, THF-d$_8$) of complex **2**.

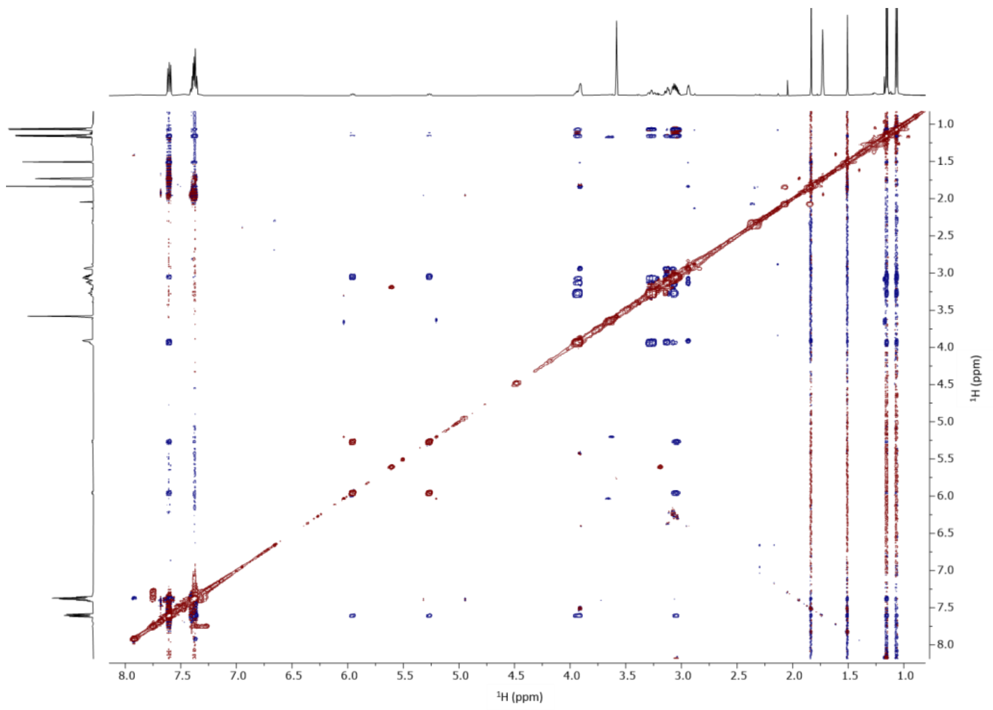

Figure S17 – ^{1}H,^{1}H-NOESY (noesygpphzs) (600.2 MHz, THF-d$_8$) spectrum of complex **2**.

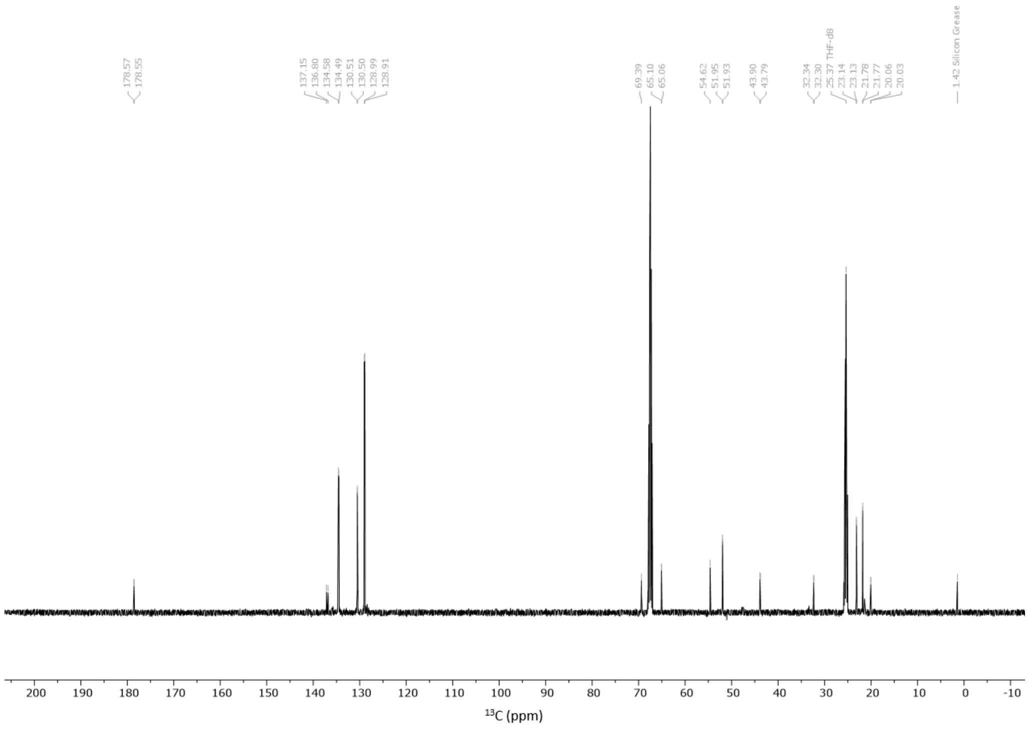

Figure S18 – ^{13}C{^{1}H} NMR (125.8 MHz, THF-d$_8$) spectrum of complex **2**.

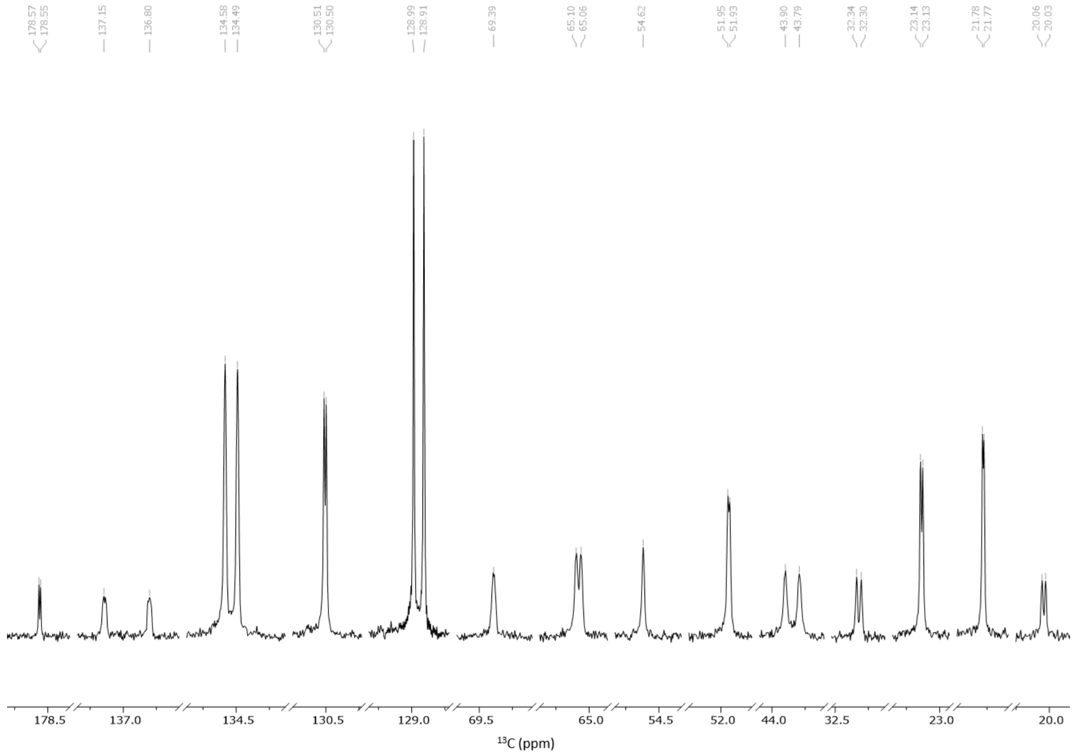

Figure S19 – Enlarged **$^{13}C\{^1H\}$ NMR (125.8 MHz, THF-d_8)** peaks of complex **2**.

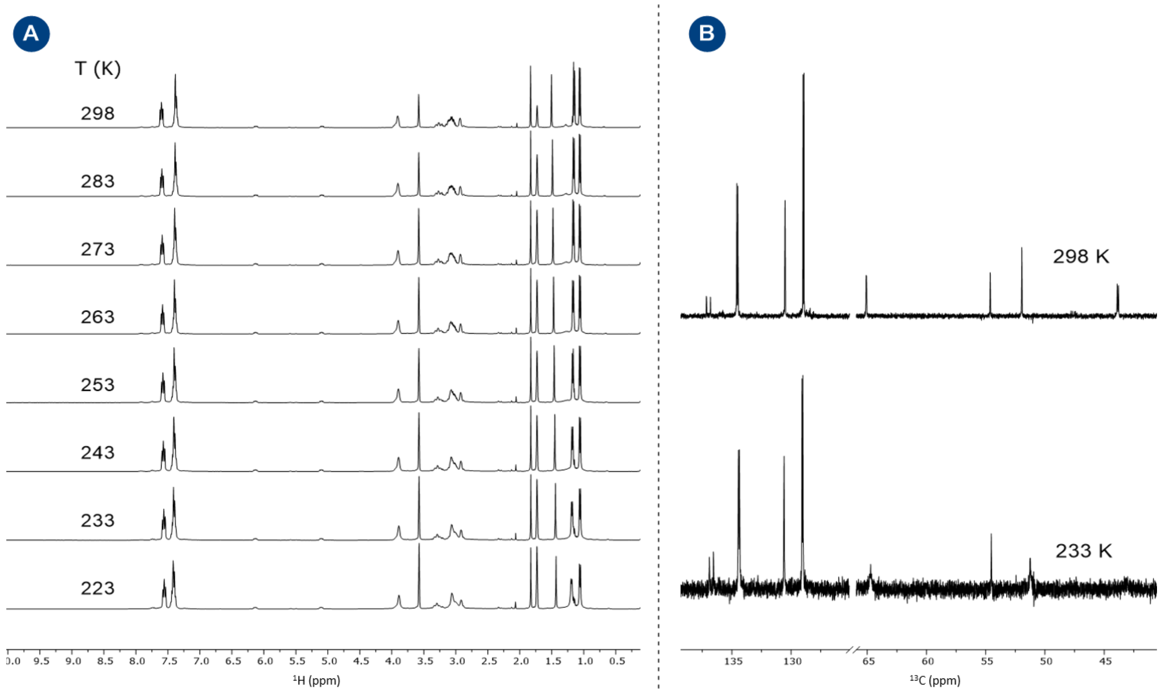

Figure S20 – Variable temperature study of complex **2**: **^1H** (panel A) and **$^{13}C\{^1H\}$** (panel B) **NMR** spectra showing no distinct appearance of further signal and only minor line broadening pronounced in the indicated regions.

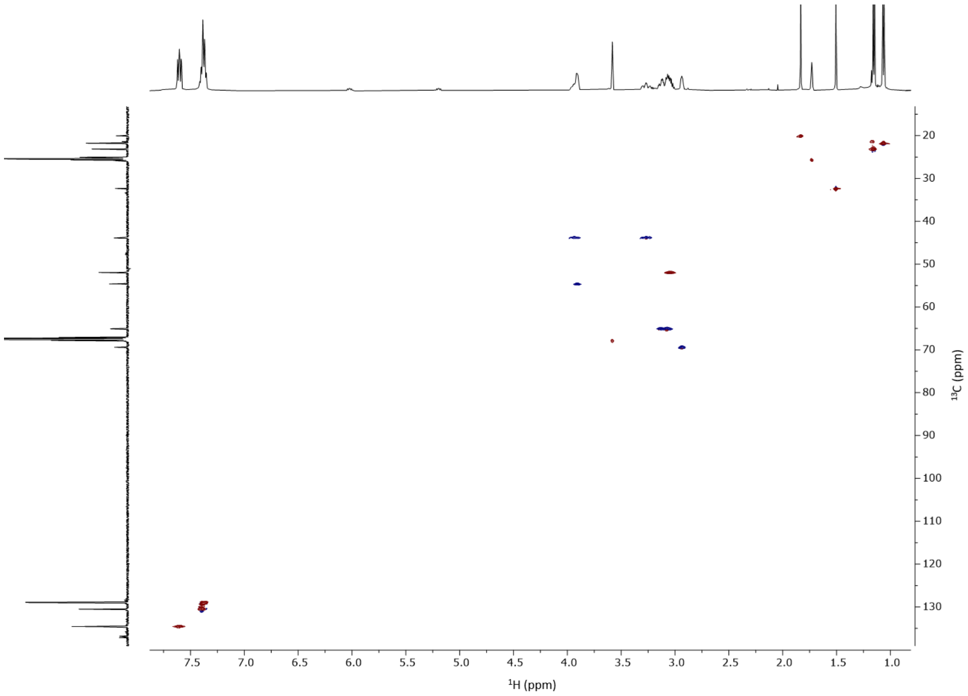

Figure S21 – Multiplicity edited **^{1}H-^{13}C HSQC (hsqcedetgp) (499.9, 125.70 MHz, THF-d$_8$)** experiment of complex **2**.

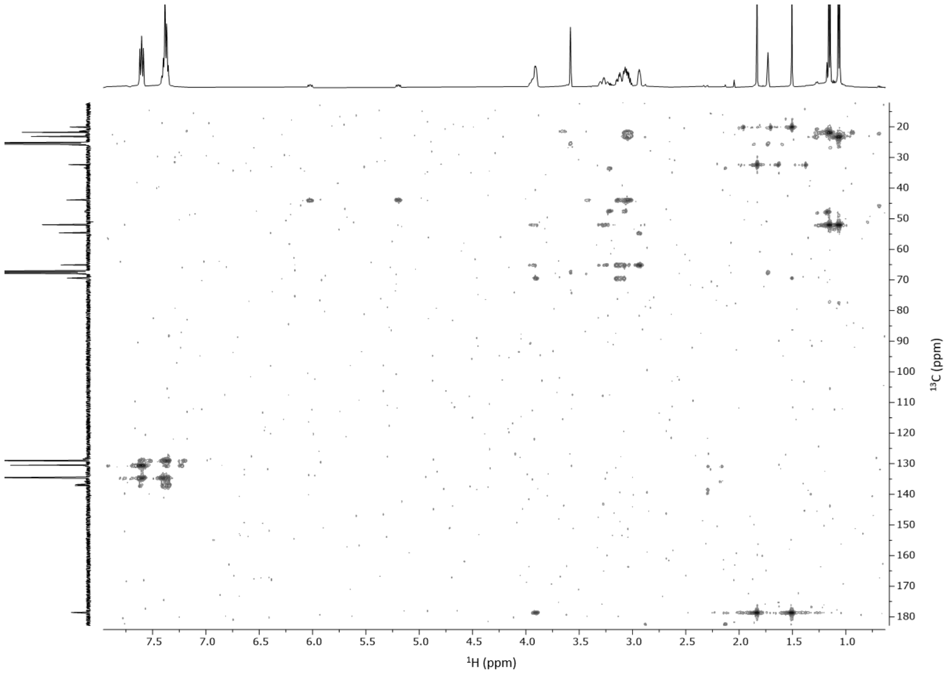

Figure S22 – ^{1}H-^{13}C HMBC (hmbcetgpl3nd) (499.9, 125.70 MHz, THF-d$_8$) experiment of complex **2**.

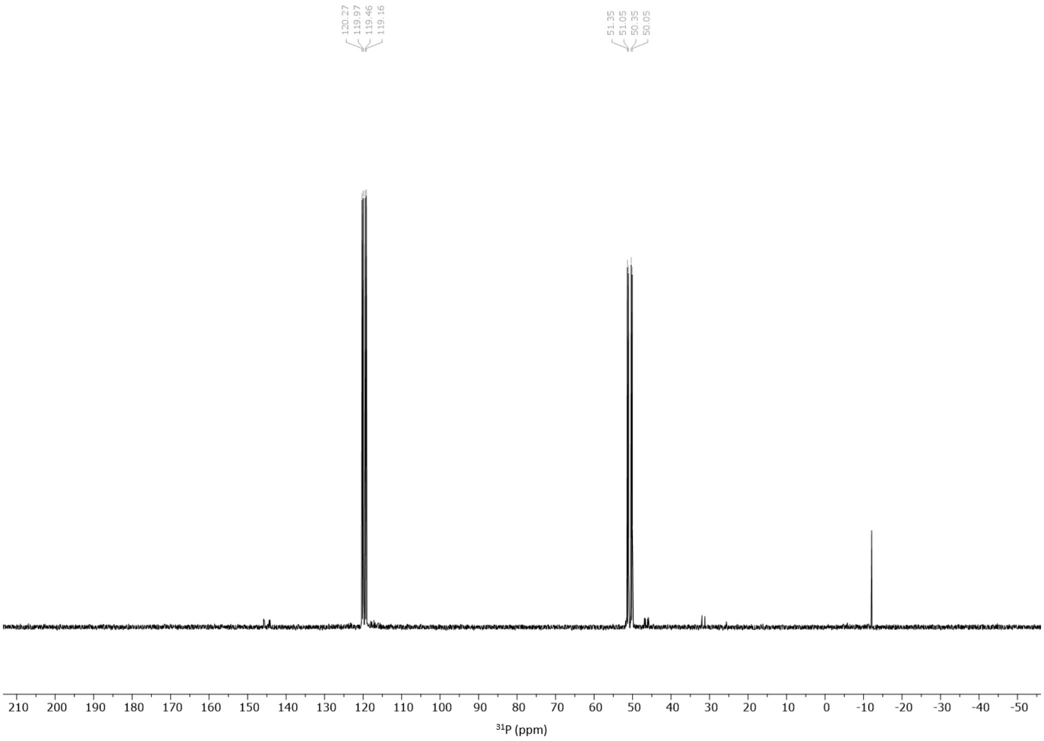

Figure S23 – ^{31}P{^{1}H} (202.4 MHz, THF-d$_8$) spectrum of complex **2**.

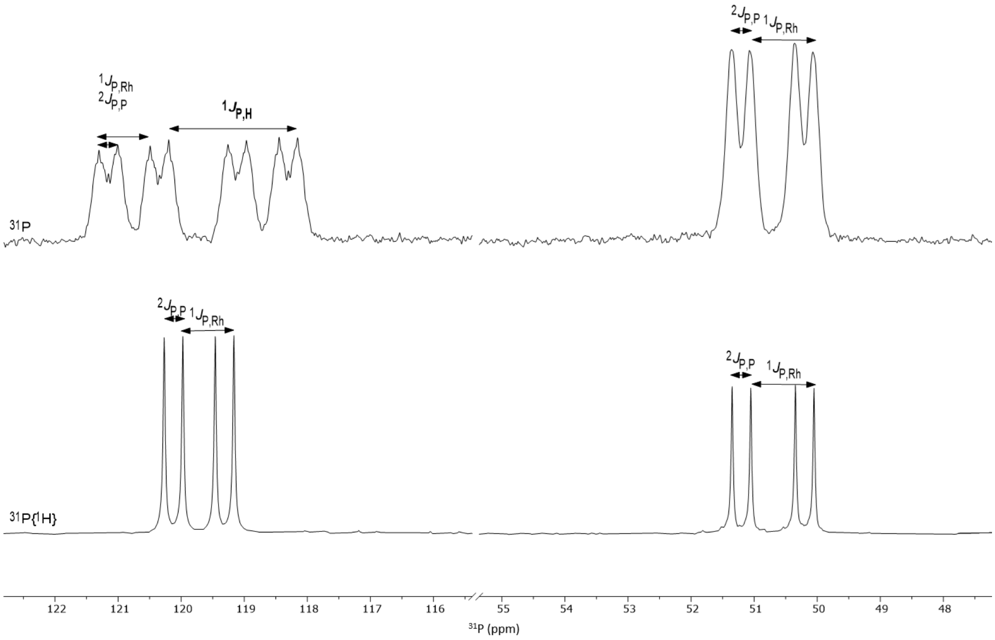

Figure S24 – Comparison of **^{31}P** and **^{31}P{^{1}H} (202.4 MHz, THF-d$_8$)** spectra of complex **2**.

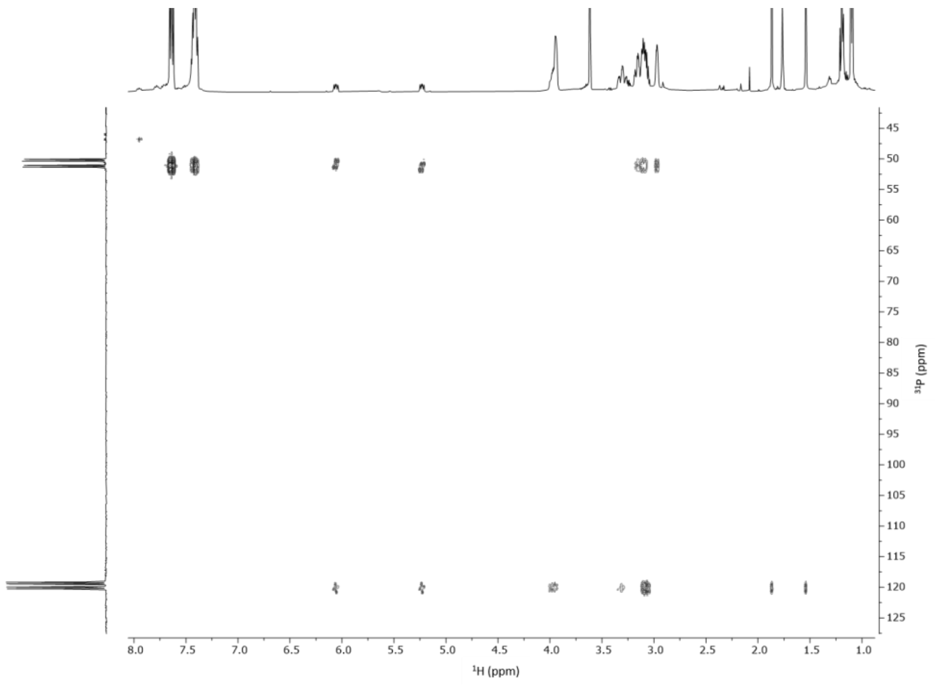

Figure S25 – ^{1}H-^{31}P HMBC (hmbcgpndqf) (499.9, 202.4 MHz, THF-d$_8$) experiment of complex **2**.

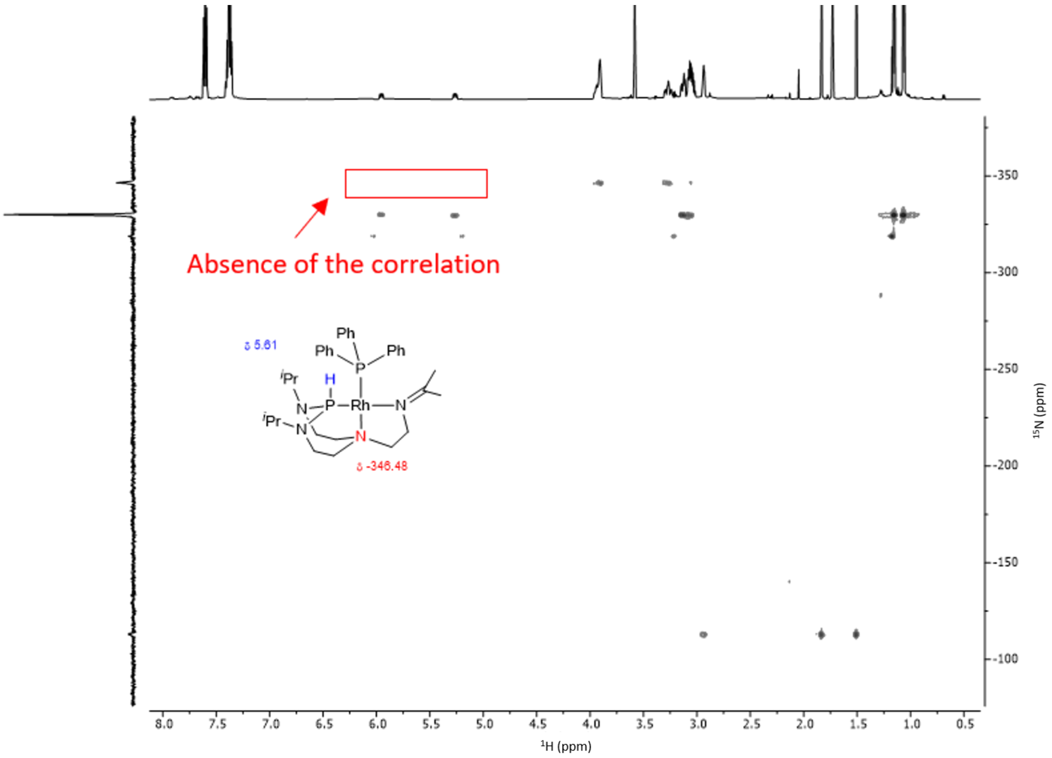

Figure S26 – ^{1}H-^{15}N HMBC (hmbcf3gpndqf) (600.3, 60.8 MHz, THF-d$_8$) experiment of complex **2**.

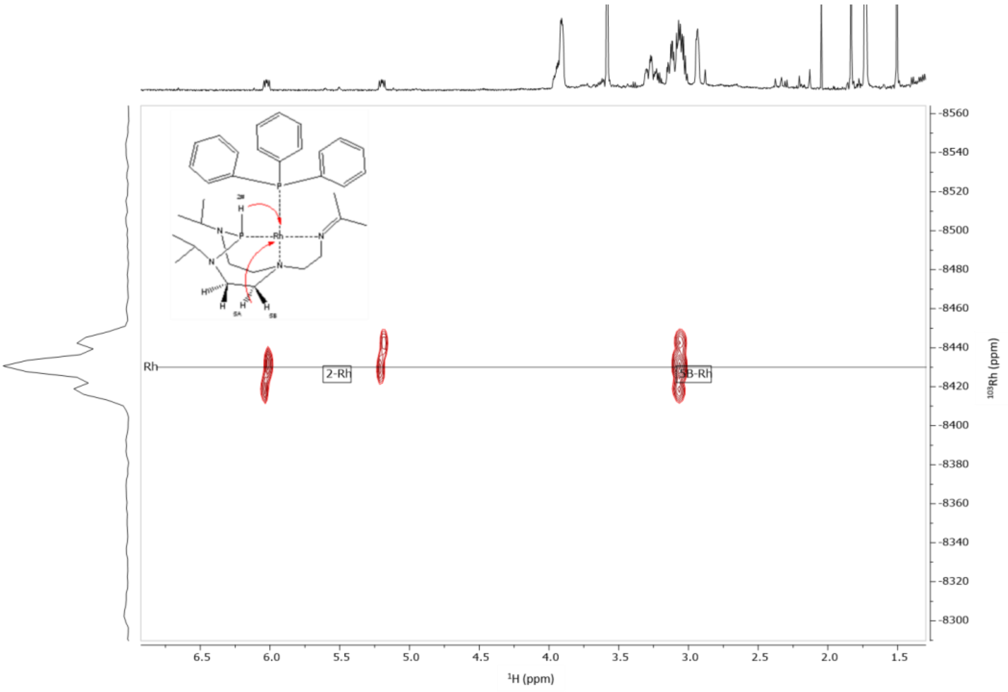

Figure S27 – **^{1}H-^{103}Rh HMBC (hmbcgpndqf) (499.9, 15.8 MHz, THF-d$_8$)** experiment of complex **2**.

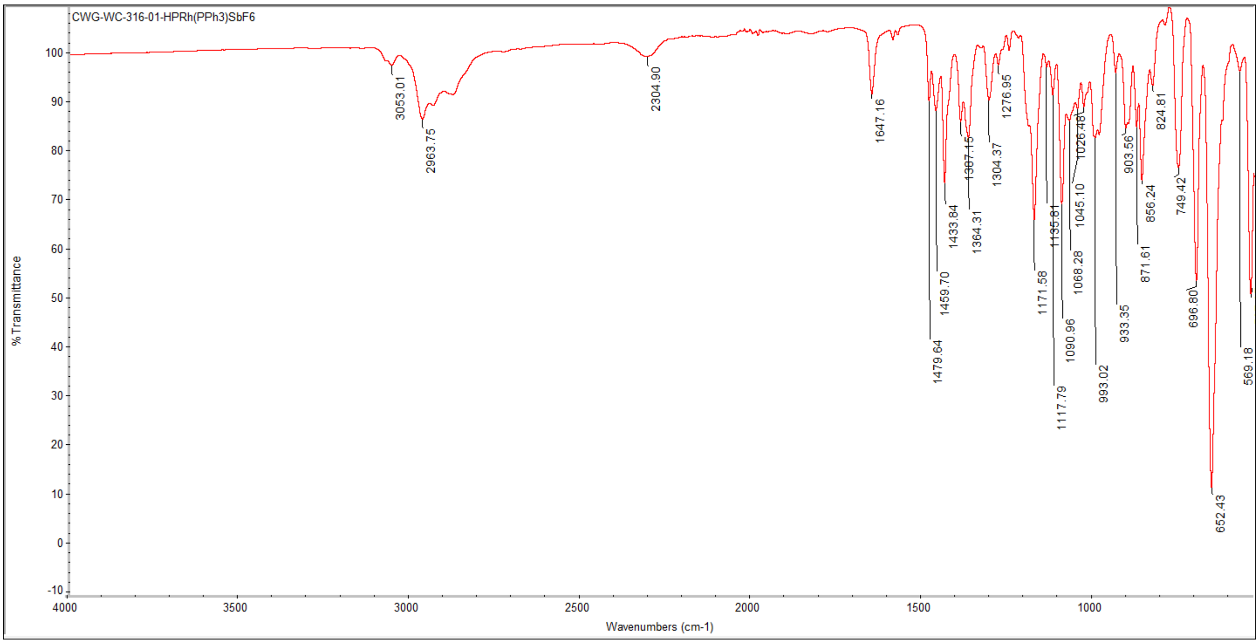

Figure S28 – **ATR-IR spectrum** of complex **2**.

Complex 3

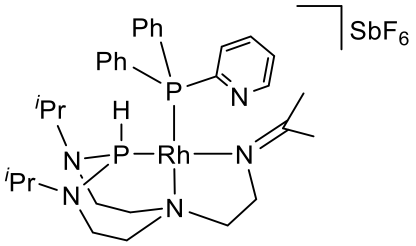

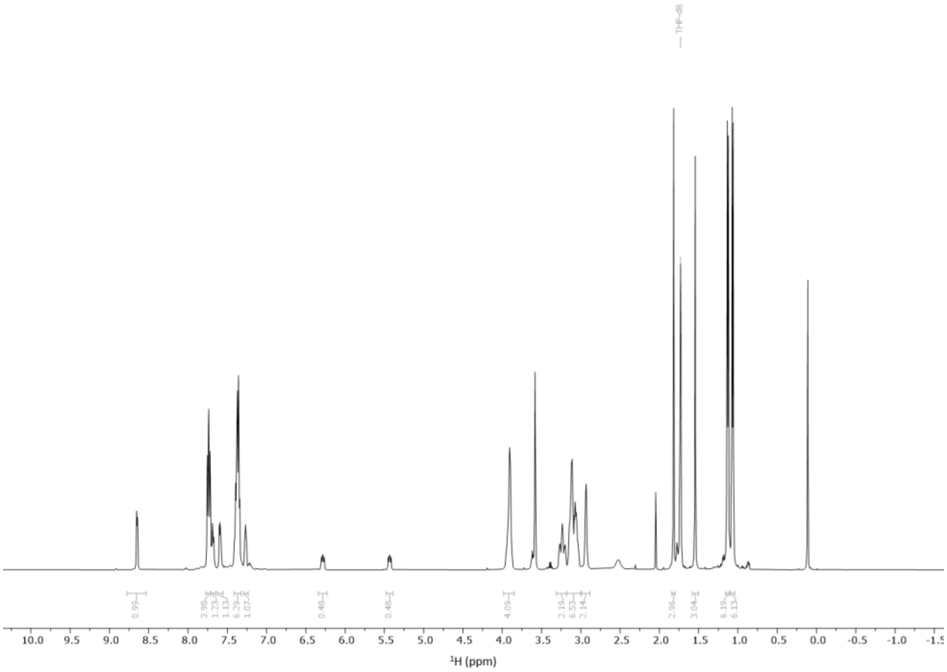

Figure S29 – ^{1}H NMR (499.9 MHz, THF-d$_8$) spectrum of complex **3**.

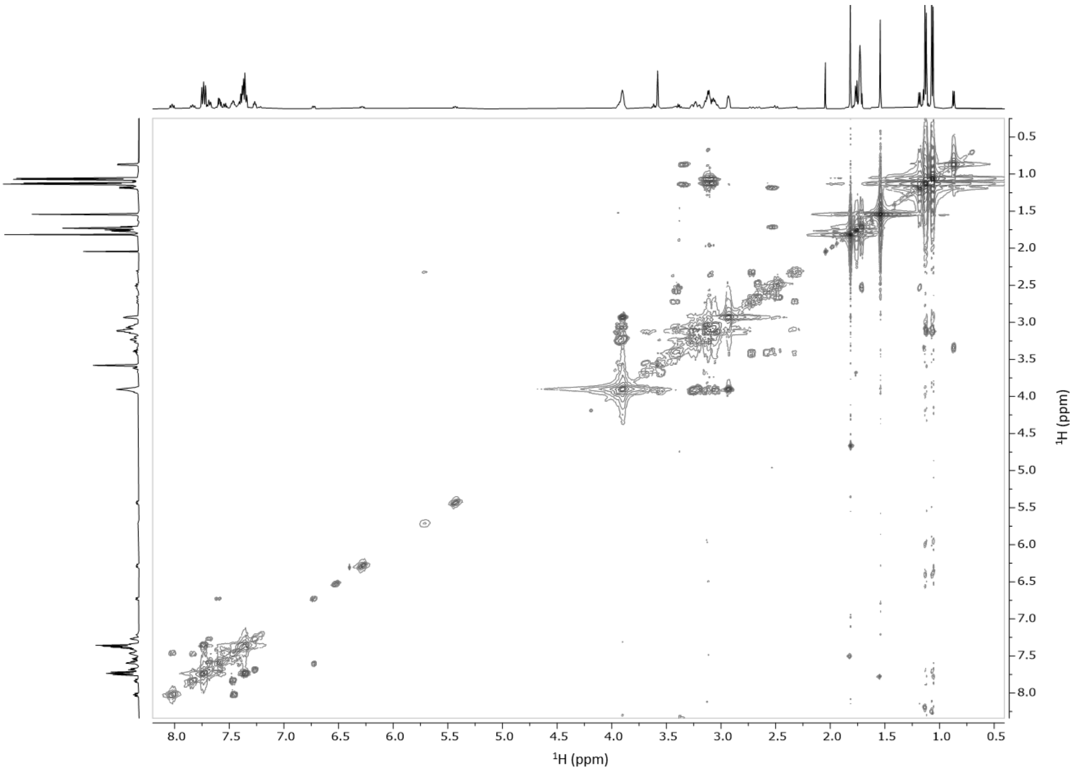

Figure S30 – DQF ^{1}H,^{1}H-COSY (cosygpmfphpp) (499.9 MHz, THF-d$_8$) of complex **3**.

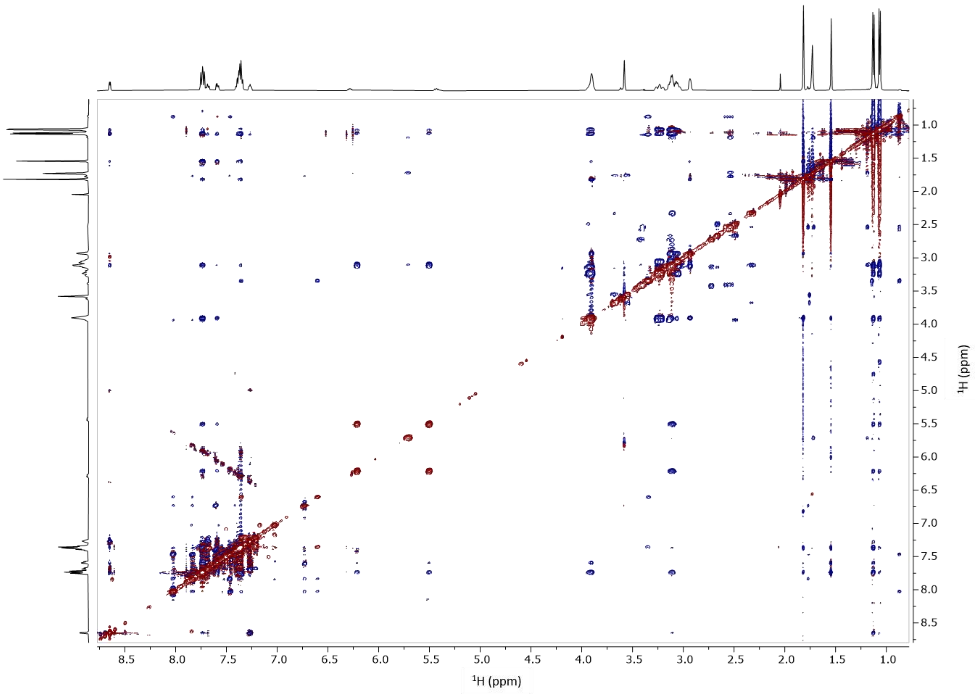

Figure S31 – ^{1}H,^{1}H-NOESY (noesygpphzs) (600.2 MHz, THF-d$_8$) spectrum of complex **3**.

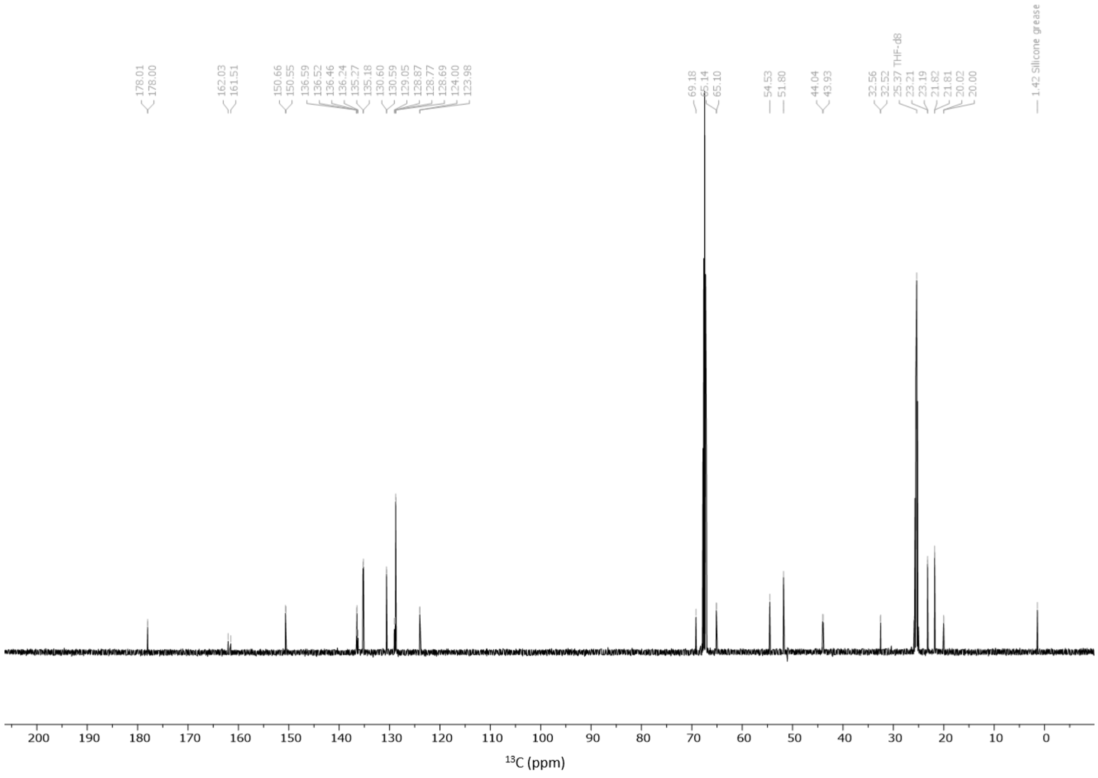

Figure S32 – $^{13}C\{^1H\}$ NMR (125.8 MHz, THF-d$_8$) spectrum of complex **3**.

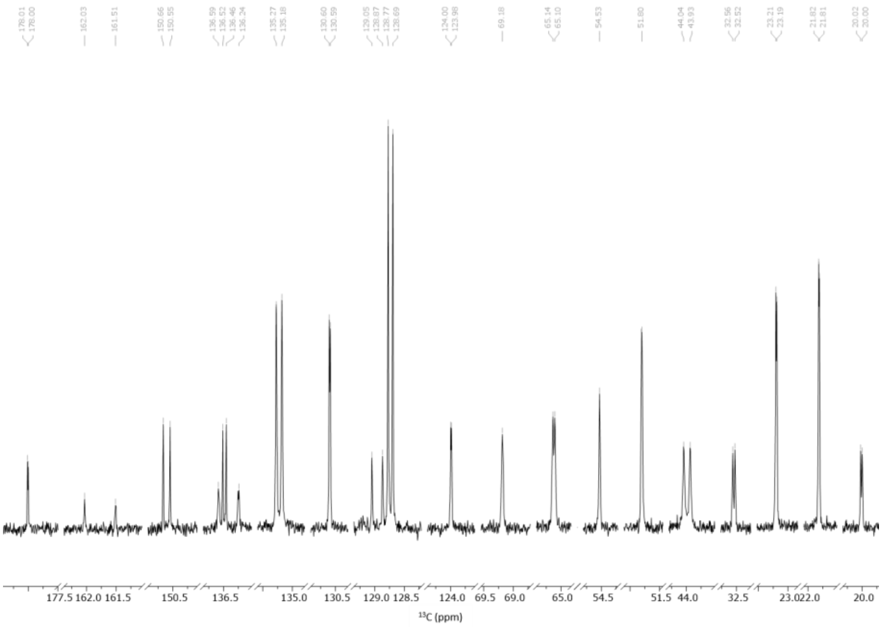

Figure S33 – Enlarged **$^{13}C\{^1H\}$ NMR (125.8 MHz, THF-d$_8$)** peaks of complex **3**.

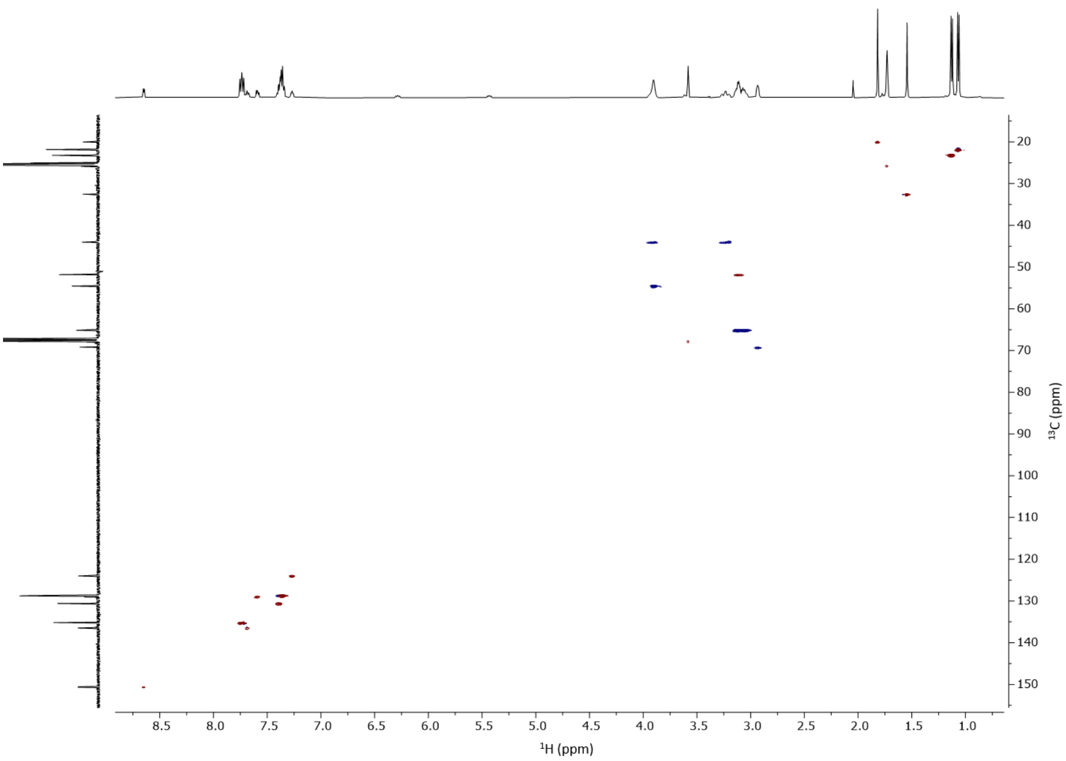

Figure S34 – Multiplicity edited **^{1}H-^{13}C HSQC (hsqcedetgp) (499.9, 125.7 MHz, THF-d$_8$)** experiment of complex **3**.

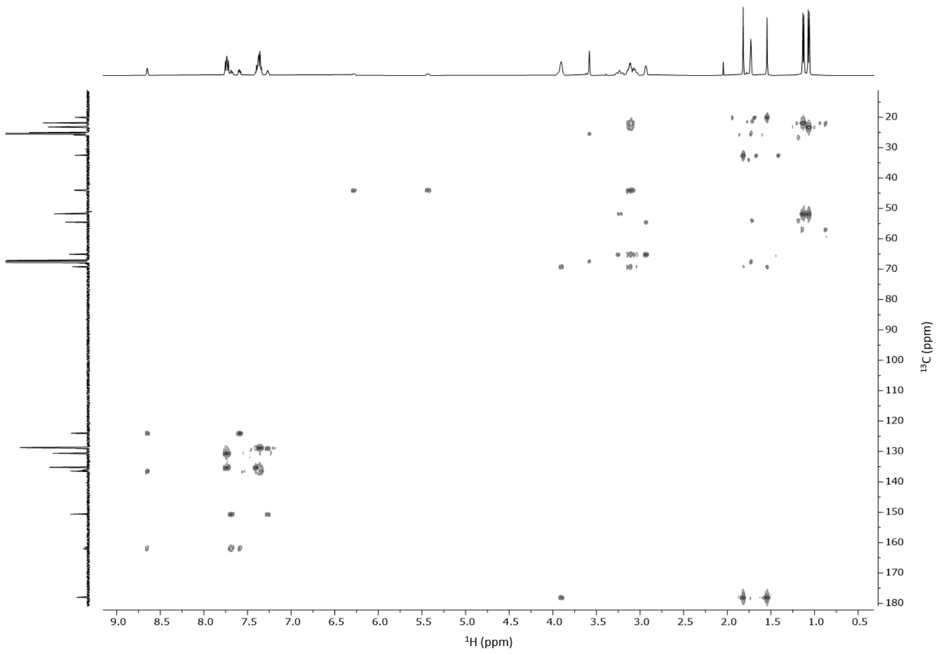

Figure S35 – ^{1}H-^{13}C HMBC (hmbcetgpl3nd) (499.9, 125.7 MHz, THF-d$_8$) experiment of complex **3**.

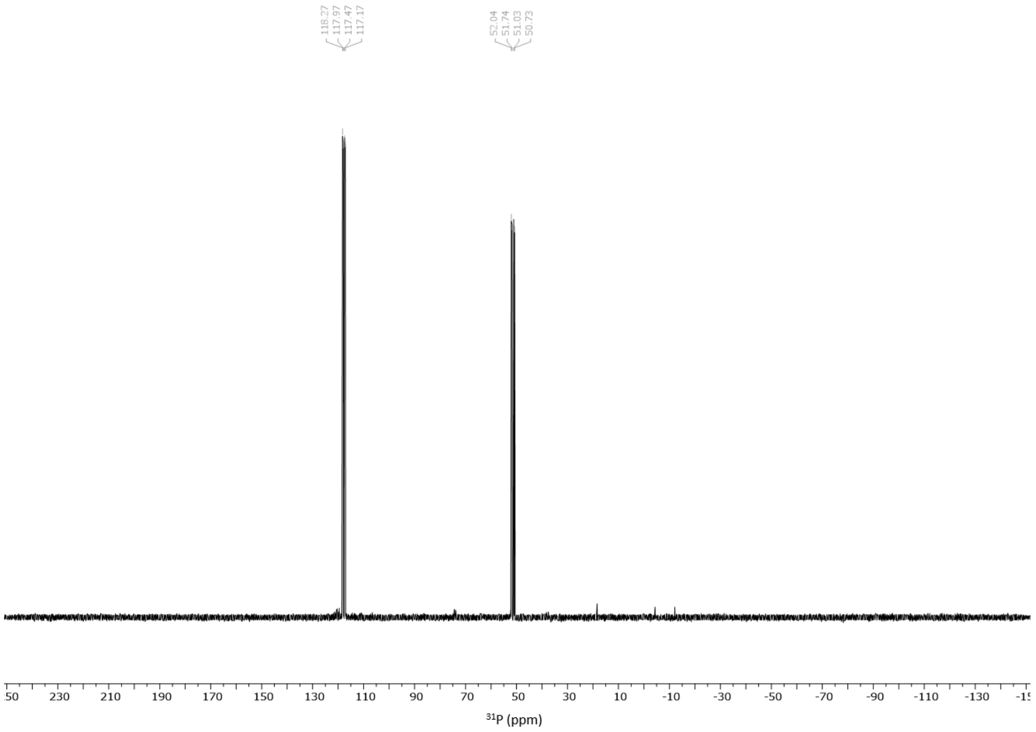

Figure S36 – ^{31}P{^{1}H} NMR (202.4 MHz, THF-d$_8$) spectrum of complex **3**.

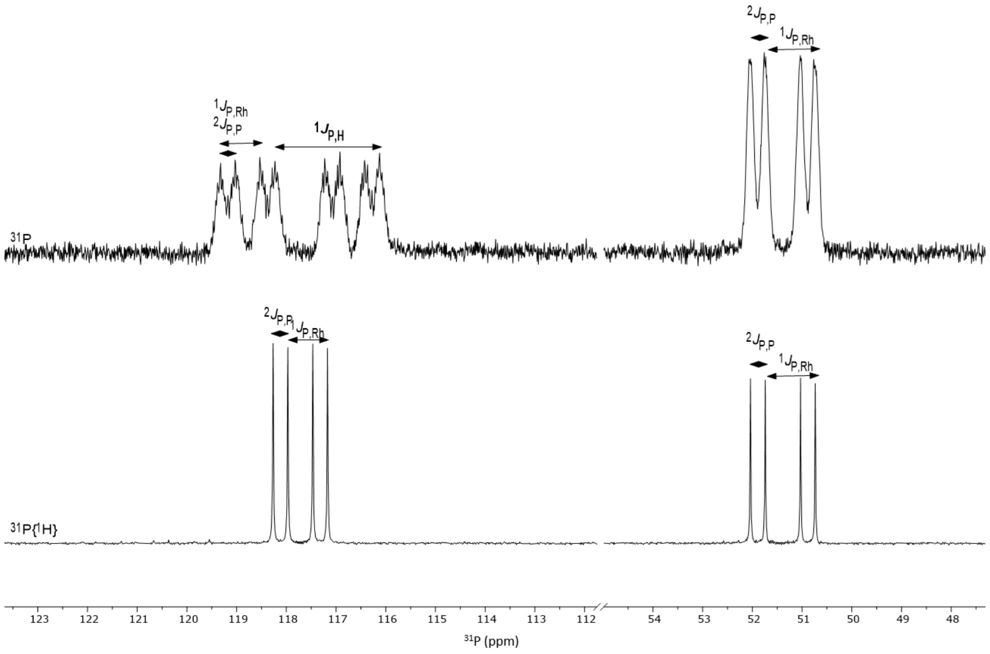

Figure S37 – Comparison of **^{31}P** and **^{31}P{^{1}H} NMR (202.4 MHz, THF-d$_8$)** spectra of complex **3**.

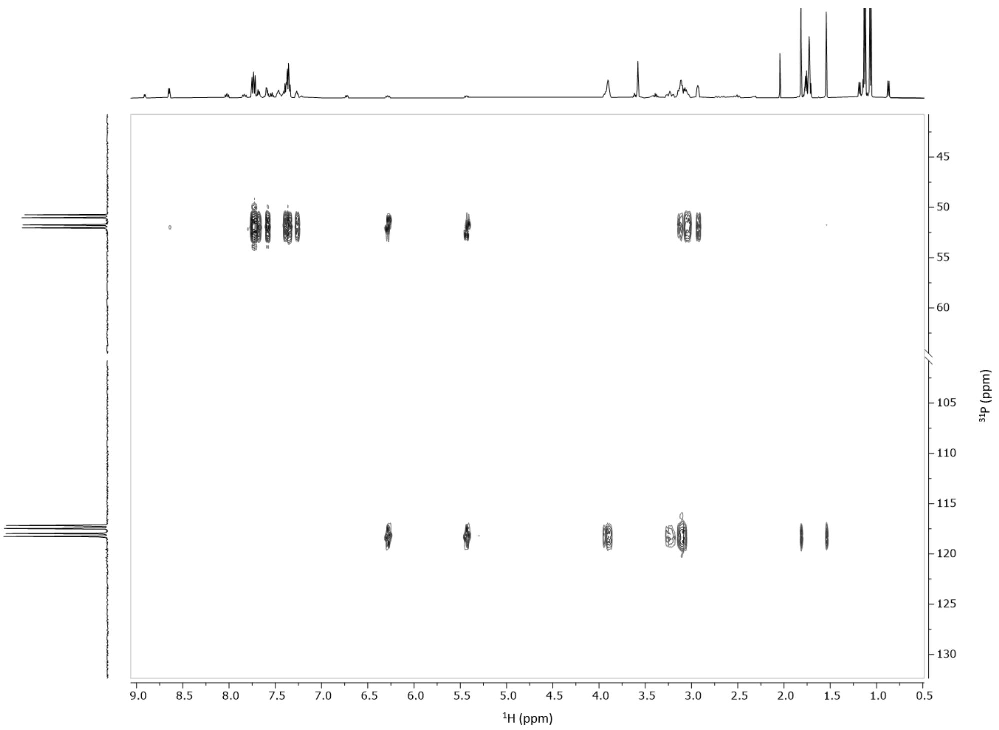

Figure S38 – ¹H-³¹P HMBC (hmbcgpndqf) (499.9, 202.4 MHz, THF-d8) experiment of complex **3**.

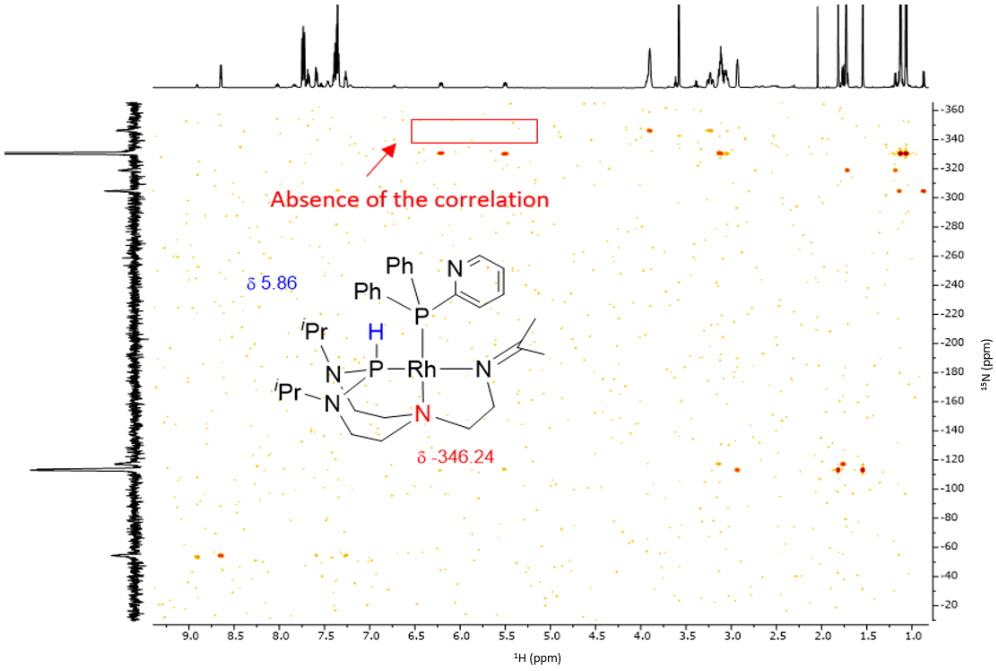

Figure S39 – ¹H-¹⁵N HMBC (hmbcf3gpndqf) (600.3, 60.8 MHz, THF-d8) experiment of complex **3**.

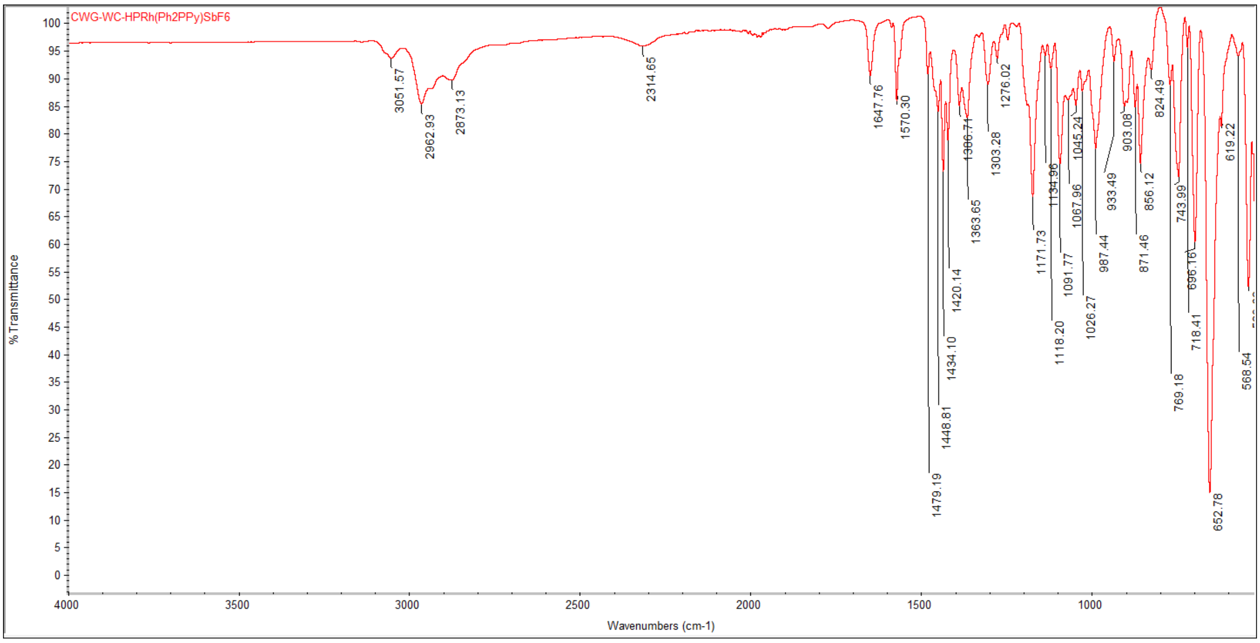

Figure S40 – ATR-IR spectrum of complex **3**.

4. Diffusion measurement

For measuring diffusion constants, the convection-corrected *dstebpgp3s* pulse sequence (Figure S41) using a double-stimulated echo experiment, bipolar gradients, and minimization of eddy currents, suggested by *Jerschow and Müller*[2] was used, as implemented in Topspin 3.6.2.[3]

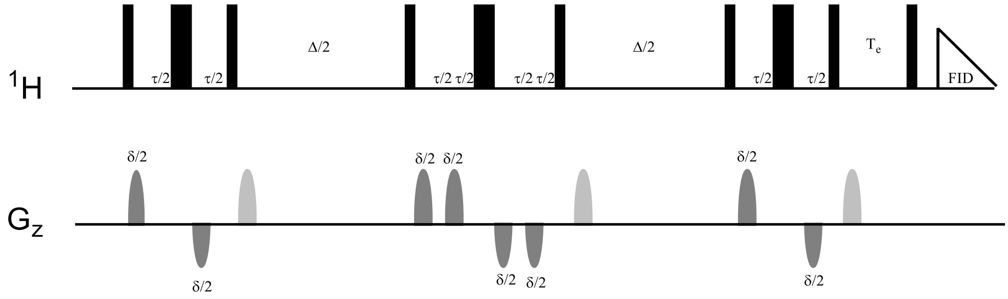

Figure S41 – Visualization of the *dstebgp3s* pulse sequence.[2]

The measurements were carried out at 298K on a 400 MHz spectrometer, using either 16 or 32 scans, depending on the solute concentration and 45 different gradient strengths. Sufficiently long relaxation delays were chosen (prior determination of T_1 values was conducted) to ensure full relaxation. Δ and δ were adjusted to give a reasonable signal decay to ensure a high-quality fit. DOSY transformation using the Peak Fit method, as implemented in MestReNova,[4] was used as a comparison, but the diffusion constants were determined by employing an exponential fit ($y = a \cdot e^{bx}$) using the Levenberg-Marquardt algorithm,[5] as implemented in OriginPro2019b.[6] The equations 1-3, were used, as suggested in the original literature[2] to obtain different plots and fitting data for complex **1** (Figure S42, Table S1), complex **2** (Figure S43, Table S2), and complex **3** (Figure S44, Table S3).

$$\boldsymbol{y} = I_0 \cdot e^{-D\boldsymbol{x}} \tag{1}$$

$$\boldsymbol{x} = (\gamma \cdot \delta \cdot \boldsymbol{G})^2 \cdot \left(\Delta + \frac{4}{3}\delta + \frac{3}{2}\tau\right) \tag{2}$$

$$\boldsymbol{y} = \boldsymbol{I} \tag{3}$$

D = measured diffusion constant, *G* = z-gradient strength, I_0 = integral with *G*=0, δ = length of bipolar gradient pulse, Δ = diffusion delay, γ = ^{1}H gyromagnetic ratio, τ = gradient recovery delay, *I* = integral at corresponding *G*.

In order to obtain more reliable diffusion values, only values for which $R^2 > 0.996$ is fulfilled were considered for arithmetic averaging. The error on the final value was estimated by the standard deviation from the mean value.

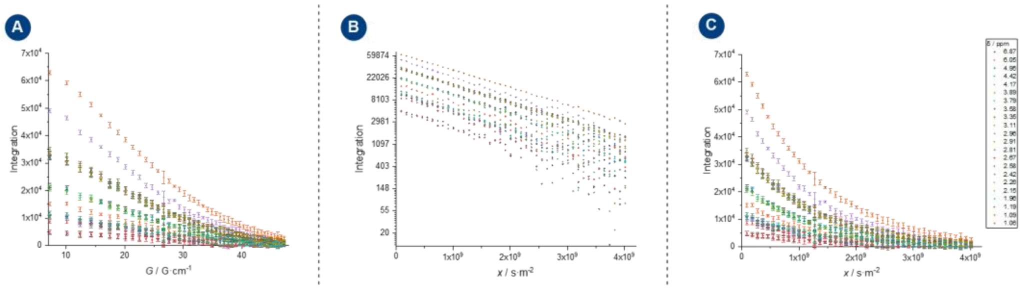

Figure S42 – Plot of different diffusion data of complex **1**. Panel A: integration vs. gradient strength; panel B: integration vs. x specified in equation 2 with a natural logarithmic scale; panel C: integration vs. x specified in equation 2. Panel C was used for fitting.

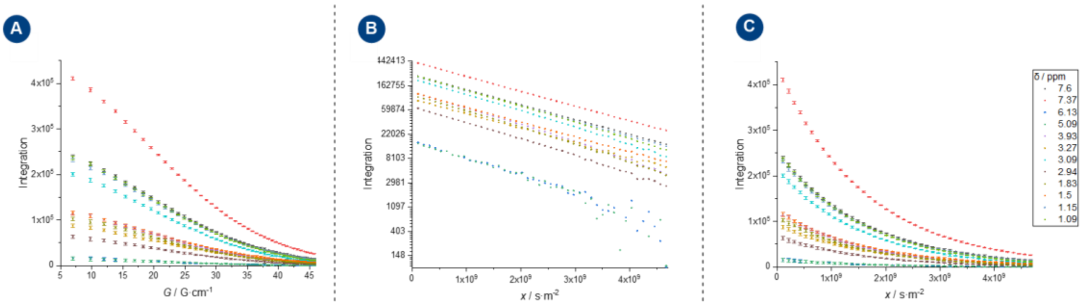

Figure S43 – Plot of different diffusion data of complex **2**. Panel A: integration vs. gradient strength; panel B: integration vs. x specified in equation 2 with a natural logarithmic scale; panel C: integration vs. x specified in equation 2. Panel C was used for fitting.

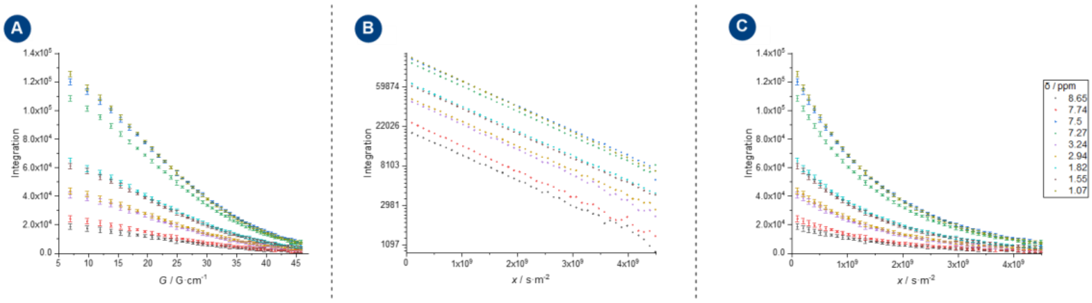

Figure S44 – Plot of different diffusion data of complex **3**. Panel A: integration vs. gradient strength; panel B: integration vs. x specified in equation 2 with a natural logarithmic scale; panel C: integration vs. x specified in equation 2. Panel C was used for fitting.

Table S1 – Values obtained from the exponential fit of the diffusion data from complex **1**.

	I_0		D / $m^2 \cdot s^{-1}$		Statistics
δ / ppm	Value	Standard Error	Value	Standard Error	Adj. R^2
6.87	5.4E+03	1E+02	-8.14E-10	2E-11	0.98049
6.05	5.1E+03	1E+02	-8.45E-10	3E-11	0.97787
4.95	1.14E+04	2E+02	-8.47E-10	2E-11	0.98841
4.42	1.17E+04	2E+02	-9.00E-10	2E-11	0.98991
4.17	1.20E+04	3E+02	-9.12E-10	3E-11	0.98036
3.89	2.33E+04	2E+02	-8.39E-10	9E-12	0.99738
3.79	1.17E+04	2E+02	-9.08E-10	2E-11	0.98911
3.58	1.15E+04	2E+02	-9.23E-10	2E-11	0.99163
3.35	3.82E+04	3E+02	-8.43E-10	9E-12	0.99739
3.11	1.70E+04	3E+02	-8.75E-10	2E-11	0.99103
2.96	3.469E+04	9E+01	-8.00E-10	3E-12	0.99971
2.91	2.23E+04	1E+02	-8.13E-10	7E-12	0.99820
2.81	2.37E+04	2E+02	-8.13E-10	9E-12	0.99743
2.67	9.7E+03	2E+02	-8.22E-10	2E-11	0.98341
2.58	3.50E+04	3E+02	-8.25E-10	8E-12	0.99772
2.42	1.30E+04	2E+02	-8.62E-10	2E-11	0.99110
2.26	5.31E+04	3E+02	-8.52E-10	7E-12	0.99835
2.15	3.471E+04	9E+01	-8.04E-10	3E-12	0.99970
1.96	2.37E+04	2E+02	-8.57E-10	1E-11	0.99614
1.19	3.613E+04	8E+01	-7.85E-10	2E-12	0.99978
1.09	3.43E+04	1E+02	-8.02E-10	4E-12	0.99949
1.06	6.80E+04	1E+02	-7.80E-10	2E-12	0.99985

The averaged diffusion value for complex **1** is $D = 8.2 \pm 0.2 \cdot 10^{-10}$ $m^2 \cdot s^{-1}$.

Table S2 – Values obtained from exponential fit of the diffusion data from complex **2**.

	I_0		D / m^2·s^{-1}		Statistics
δ / ppm	Value	Standard Error	Value	Standard Error	Adj. R^2
7.6	2.549E+05	2E+02	6.171E-10	7E-13	0.99997
7.37	4.384E+05	3E+02	6.153E-10	7E-13	0.99997
6.13	1.69E+04	2E+02	7.2E-10	1E-11	0.99447
5.09	1.63E+04	3E+02	7.1E-10	2E-11	0.98997
3.93	1.251E+05	4E+02	6.75E-10	3E-12	0.99948
3.27	9.35E+04	2E+02	6.02E-10	2E-12	0.99969
3.09	2.166E+05	5E+02	6.60E-10	2E-12	0.99979
2.94	6.81E+04	2E+02	6.64E-10	3E-12	0.99952
1.83	1.113E+05	4E+02	6.68E-10	3E-12	0.99948
1.5	1.225E+05	2E+02	6.14E-10	1E-12	0.99986
1.15	2.462E+05	3E+02	6.27E-10	1E-12	0.99994
1.09	2.547E+05	4E+02	6.48E-10	1E-12	0.99988

The averaged diffusion value for complex **2** is $D = 6.4 \pm 0.2 \cdot 10^{-10}$ m^2·s^{-1}.

Table S3 – Values obtained from exponential fit of the diffusion data from complex **3**.

	I_0		D / $m^2 \cdot s^{-1}$		Statistics
δ / ppm	Value	Standard Error	Value	Standard Error	Adj. R^2
8.65	1.999E+04	6E+01	6.28E-10	3E-12	0.99953
7.74	2.570E+04	7E+01	6.44E-10	2E-12	0.99967
7.5	1.287E+05	2E+02	6.23E-10	2E-12	0.99985
7.27	1.158E+05	2E+02	6.37E-10	1E-12	0.99991
3.24	4.40E+04	1E+02	6.38E-10	2E-12	0.99969
2.94	4.676E+04	8E+01	6.22E-10	1E-12	0.99985
1.82	6.84E+04	1E+02	6.26E-10	1E-12	0.99988
1.55	6.526E+04	5E+01	6.296E-10	7E-13	0.99997
1.07	1.333E+05	1E+02	6.493E-10	9E-13	0.99995

The averaged diffusion value for complex **3** is $D = 6.33 \pm 0.08 \cdot 10^{-10}$ $m^2 \cdot s^{-1}$.

5. Kinetic studies

In order to get information about the intramolecular conversion rate of complex **1**, equation 4 was used as a model for which the integral of the observed exchanging peak $I_{observed}$ was normalized by the integral of the corresponding irradiated peak $I_{irradiated}$ of a series of selective 1D NOESY experiments (Figure S45). The setup and adjustment of adiabatic and gradient pulses were carried out as suggested by *Keeler et al.*[7] The equilibrium observed here is assumed to be independent of substrate concentration in the diluted sample (<<1 M).

$$\frac{I_{observed}(t_{mix})}{I_{irradiated}(t_{mix})} = k_{obs} \cdot t_{mix} \qquad (4)$$

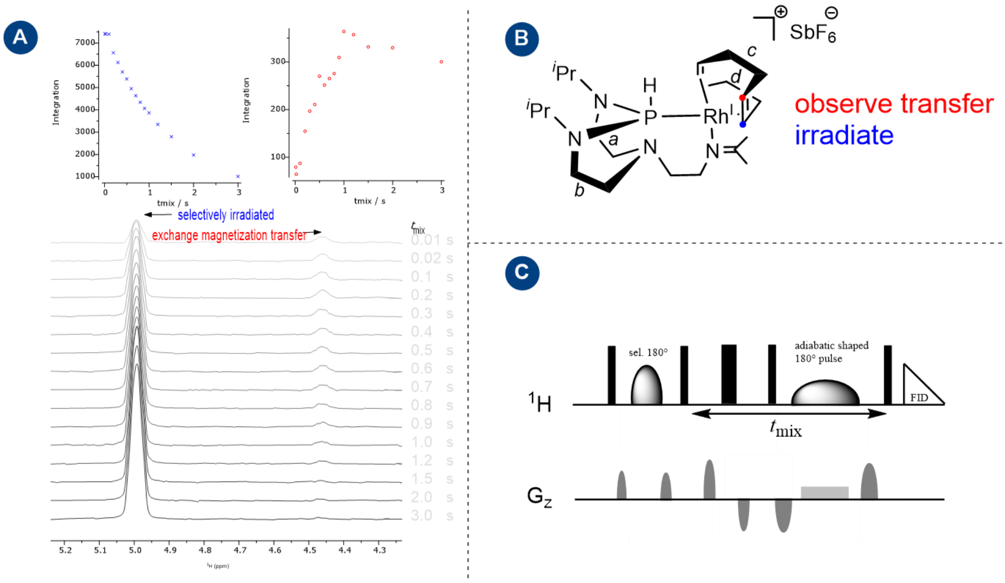

Figure S45 –EXSY build-up curve irradiating (panel A) and observing the position specified in panel B using the selnozgpzs pulse sequence (panel C).

Normalization against the initially irradiated signal $I_{irradiated}(0)$ was attempted as well, as suggested by *Gschwind et al.*[8] Both methods gave similar results as long as only the initial data points (e.g., 100 to 400 ms) were considered in case of normalizing against $I_{irradiated}(0)$ (Figure S46). Overall the normalization method described here is similarly applied and recommended for the initial rate approximation of NOE build-up[9] and gave more reliable results as additional relaxation effects are normalized.[10]

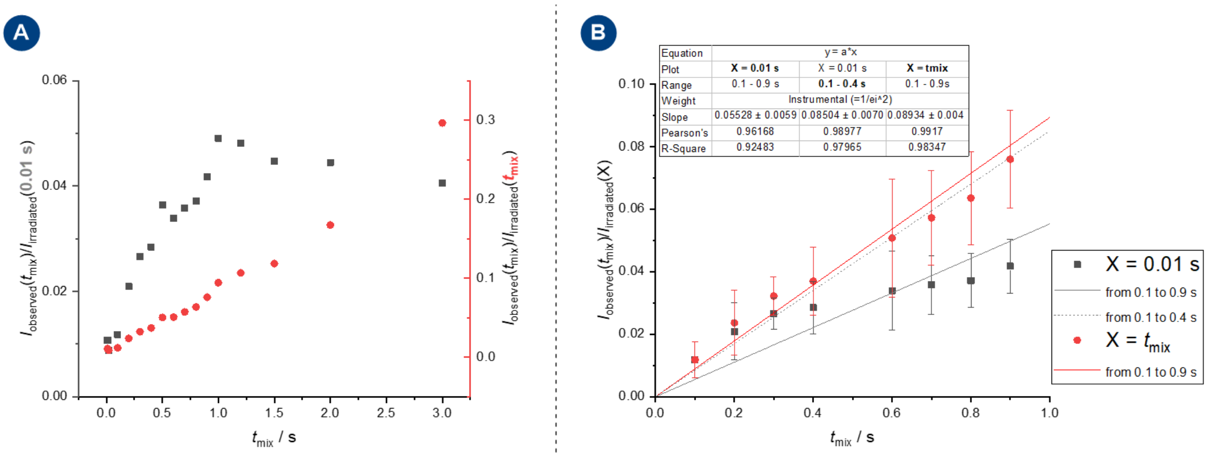

Figure S46 – Panel A: data obtained from EXSY build-up (4.42 ppm) normalized against the irradiated peak (4.96 ppm) at t_{mix} = 0.01s and normalized (black) against the irradiated peak at each mixing time (red); panel B: linear fit comparison for the two normalization procedures considering different data ranges.

As an alternative method, a selective inversion recovery method could have been envisioned, but satisfying build-up curves justified the use of the EXSY build-up.[11] In order to get higher quality results and compare different exchange sites and get an error estimate, the 1D EXSY experiment was repeated on an instrument with higher sensitivity, and different sites for irradiation were chosen. Geminal exchanging protons were not considered due to compensating nuclear Overhauser and exchange effects. Protons in severely crowded regions were not considered due to overlap and irradiation of other signals, and exchanging protons that were too close in chemical shift were not considered due to overlap of irradiated and build-up peak. The mixing times (0.001s to 0.8s) were recorded in a randomized manner for irradiation at 4.99, 3.77, 3.13, 2.68, 1.20 ppm. Linear fitting ($y = m \cdot x$, Table S4) after normalization was done from 0.03 to 0.8s as this was in a linear regime for most curves (Figure S47). The temperature of the NMR probe was calibrated prior to the measurement using a 99.8% MeOD-d_4 sample. The error on the temperature was estimated to be 1K by comparing selected temperatures with other references samples. A cautious error of 20% was estimated on the experimental k values at 3.13 ppm irradiation, which covers the slight discrepancies displayed in Figure S47.

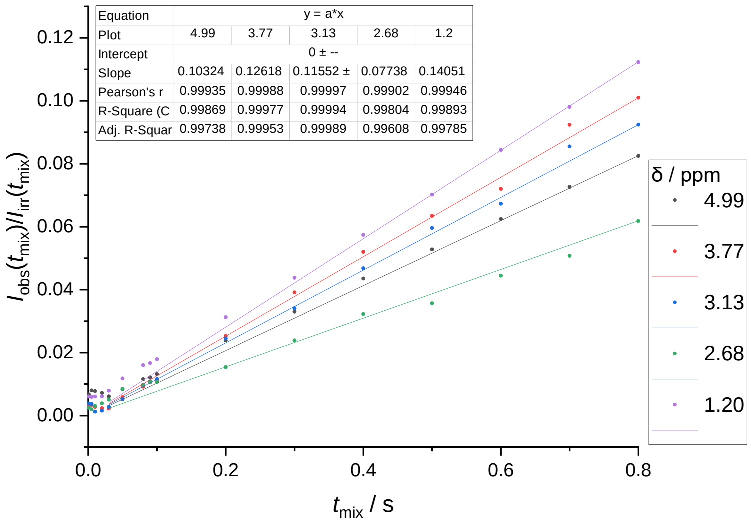

Figure S47 – Normalized integrals of the EXSY build-up of complex **1** at different mixing times and irradiation frequencies at 293.2 K used for linear regression.

Table S4 – Results from linear regression of normalized 1D-EXSY build-up intensity vs. varied mixing for complex **1** with different irradiation frequencies.

	k_{298K} / s^{-1}		Statistics
δ / ppm	Value	Standard Error	Adj. R^2
4.99	0.103	0.004	0.99738
3.77	0.1262	0.0019	0.99953
3.13	0.1155	0.0009	0.99989
2.68	0.077	0.003	0.99608
1.20	0.141	0.005	0.99785

For temperature studies, irradiation was exclusively done at 3.13 or 4.99 ppm, as they seem to yield the most reliable results. A set of 1D EXSY[i] spectra at 290, 298, 303, 308, 313, and 320 K was recorded, and the rate constants were determined at each point (Figure S48). Fitting the data to a rearranged form equation 6) of the Eyring equation (equation 5) resulted in activation entropy and enthalpy values (Figure S48, Table S5).

$$k(T) = \frac{k_B \cdot T}{h} \cdot e^{-\Delta G^{\ddagger}/RT} = \frac{k_B \cdot T}{h} \cdot e^{-\Delta H^{\ddagger}/RT} \cdot e^{\Delta S^{\ddagger}/R} \qquad (5)$$

[i] The following settings were used for data acquisition: 32 scans, 4 dummy scans, sufficiently long relaxation delay according to previously determined T_1 relaxation.

$$R \cdot \left(\ln \left(\frac{k(T)}{T} \right) + \ln \left(\frac{h}{k_B} \right) \right) = -\Delta H^{\ddagger} \cdot \frac{1000}{T} + \Delta S^{\ddagger} \qquad (6)$$

The errors were calculated according to the error propagation equations 7 and 8.[12]

$$\sigma(\Delta H^{\ddagger}) = \frac{R \cdot T_{\max} \cdot T_{\min}}{\Delta T} \sqrt{\left(\frac{\sigma(T)}{T} \right)^2 \left(\left(1 + T_{\min} \frac{\Delta L}{\Delta T} \right)^2 + \left(1 + T_{\max} \frac{\Delta L}{\Delta T} \right)^2 \right) + 2 \left(\frac{\sigma(k)}{k} \right)^2} \qquad (7)$$

$$\sigma(\Delta S^{\ddagger}) = \frac{R}{\Delta T} \sqrt{\left(\frac{\sigma(T)}{T} \right)^2 \left(T_{\max}^2 \left(1 + T_{\min} \frac{\Delta L}{\Delta T} \right)^2 + T_{\min}^2 \left(1 + T_{\max} \frac{\Delta L}{\Delta T} \right)^2 \right) + \left(\frac{\sigma(k)}{k} \right)^2 (T_{\max}^2 + T_{\min}^2)} \qquad (8)$$

With $\Delta T = T_{\max} - T_{\min}$, $\Delta L = \ln(k_{\max}/T_{\max}) - \ln(k_{\min}/T_{\min})$.

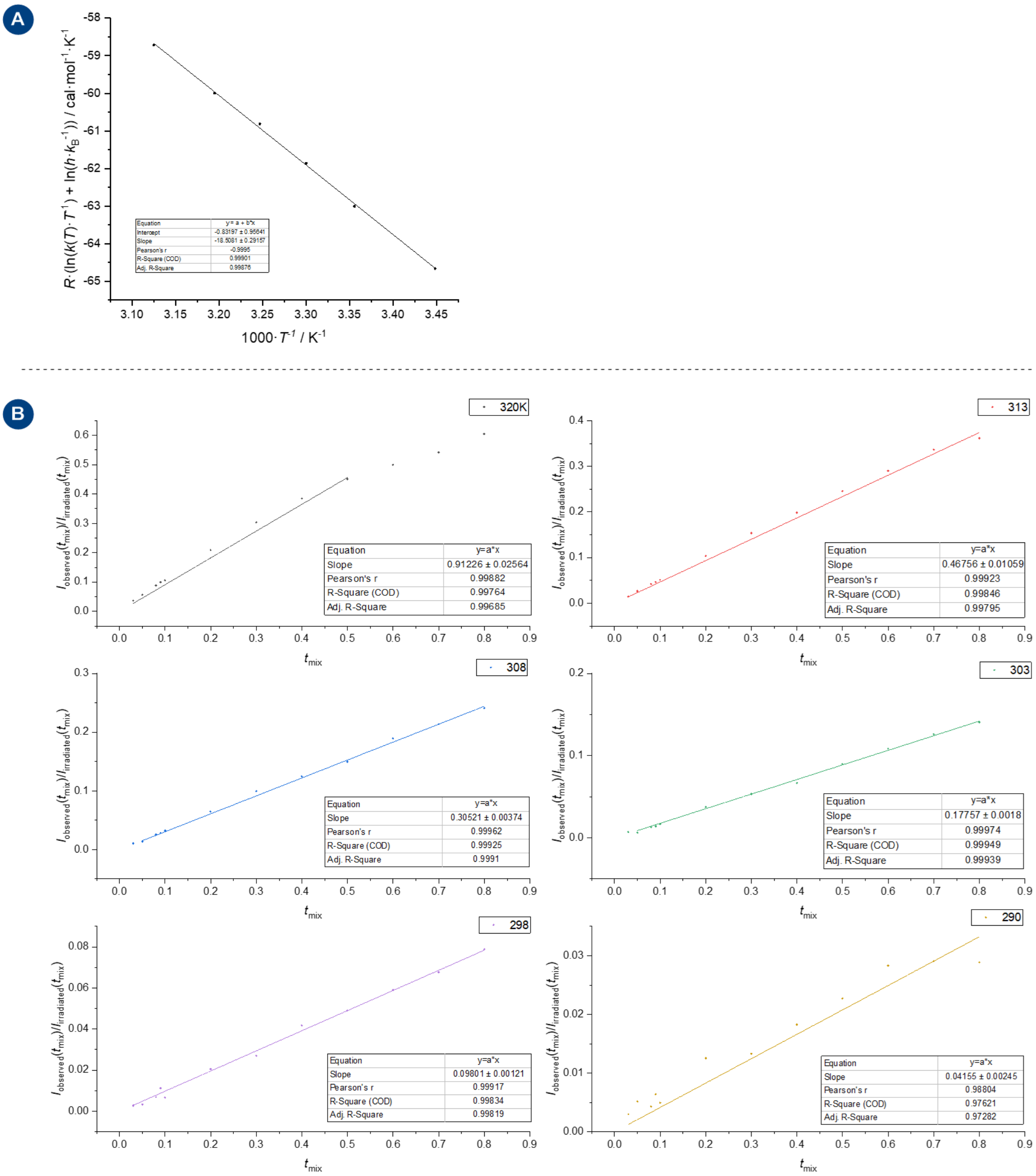

Figure S48 – Study of complex **1** in THF without LiCl. The sample was selectively excited at 3.13 ppm. Panel A: graph used for linear regression of equation 6 to obtain activation enthalpy and entropy as slope and intercept respectively; panel B: overview of the linear fits of the normalized EXSY build-up curves at different temperatures.

Table S5 – Values used for linear regression and error calculation in the EXSY temperature series of complex **1**.

T / K	k / s^{-1}	1000·T^{-1} / K^{-1}	R·(ln($k(T)$·T^{-1}) + ln(h·k_B^{-1})) / cal·mol^{-1}·K^{-1}	σ(Δ$H^‡$) / kcal·mol^{-1}	σ(Δ$S^‡$) / cal·mol^{-1}·K^{-1}
290	0.04155	3.448276	-64.80415	2.0	6.5
298	0.09801	3.355705	-63.15287	2.0	6.5
303	0.17757	3.300330	-62.00495	2.0	6.4
308	0.30521	3.246753	-60.96113	2.0	6.4
313	0.46756	3.194888	-60.14554	1.9	6.4
320	0.91226	3.125000	-58.86125	1.9	6.4

Using this approach, the activation enthalpy was determined to be $\Delta H^‡$ = 18.5 ± 1.9 kcal·mol^{-1} and the activation entropy $\Delta S^‡$ = 0.8 ± 6.4 cal·mol^{-1}·K^{-1}.

To investigate whether this exchange behavior could be altered upon the addition of an additive, LiCl was added to a new sample, and the behavior was investigated again. Upon adding LiCl (14 equiv.), a significant line broadening and starting coalescence was observed at room temperature, indicating a significantly increased exchange behavior. The temperature study was repeated in analogy to the previously described procedure but was carried out at lower temperatures to have still adequately separated lines suitable for irradiation and observable exchange on the EXSY timescale. Lineshape analysis could have been chosen as an alternate method for analyzing the kinetics, but better comparability with the previous study was expected using the same method as before. The concentration dependence of LiCl was not yet investigated. Hence, the given rate constants are kobs and likely depend on LiCl concentration, especially for catalytic amounts of LiCl. For the 1D EXSY[ii] build-up, the peak at 4.99 ppm was selectively irradiated since the broader signals of the sample made irradiating the signal at 3.13 ppm less reliable.

[ii] The following settings were used for data acquisition: 128 scans, 4 dummy scans, sufficiently long relaxation delay according to previously determined T_1 relaxation.

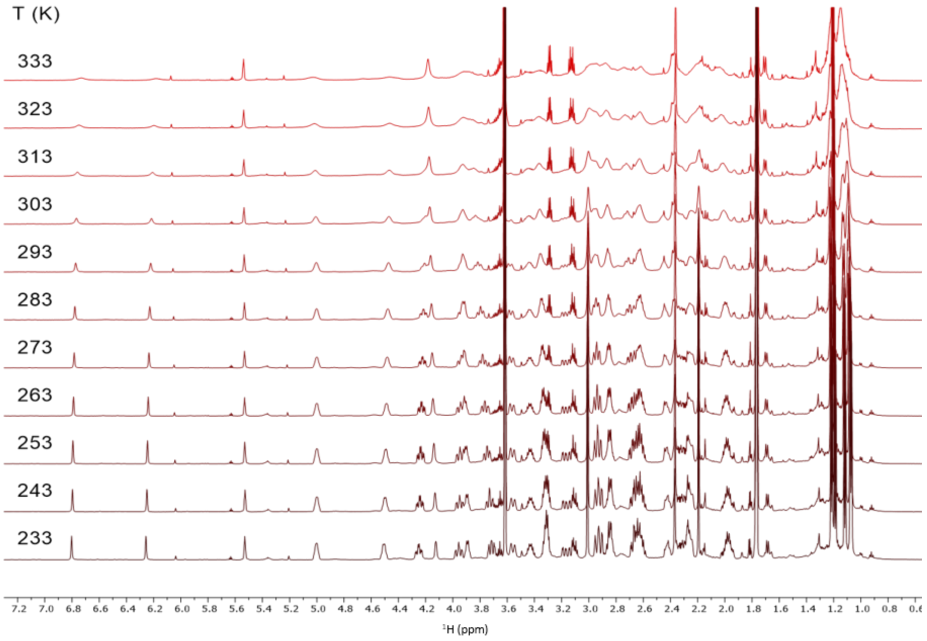

Figure S49 – VT study of complex **1** with an excess of LiCl in THF-d_8. A line broadening at higher temperatures is observed. Sharp signals correspond to minor amounts of free COD and the protonated T^{i}PrAP.

The existence of a faster exchange is directly observable by comparing the LiCl and LiCl free sample at room temperature, as shown in Figure S50.

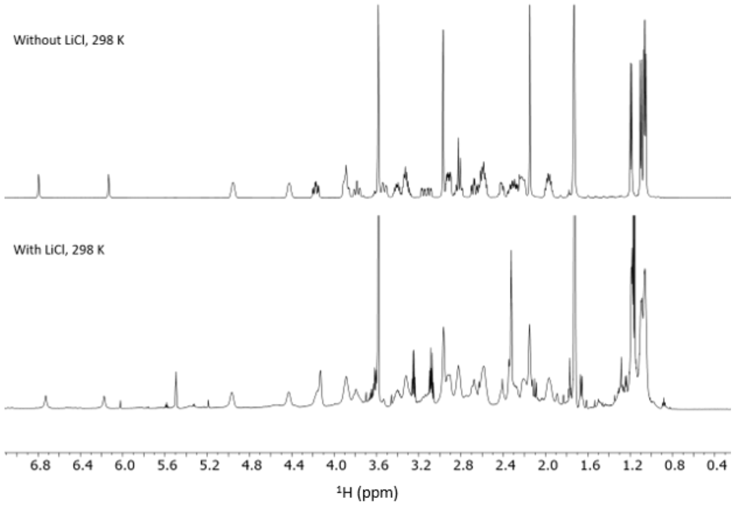

Figure S50 – Comparison of spectra of complex **1** with and without LiCl at room temperature in THF-d_8. Small impurities are observable, like the small signals at 6.1 and 5.2 ppm in the LiCl trace corresponding to the protonated T^{i}PrAP and the signal at 5.5 ppm corresponding to free COD.

To adequately study the faster exchange using the same method (EXSY), lower temperatures had to be selected and were subjected to an identical fitting and standard error analysis, as indicated above. This resulted in similar graphs in Figure S51 and data in Table S6.

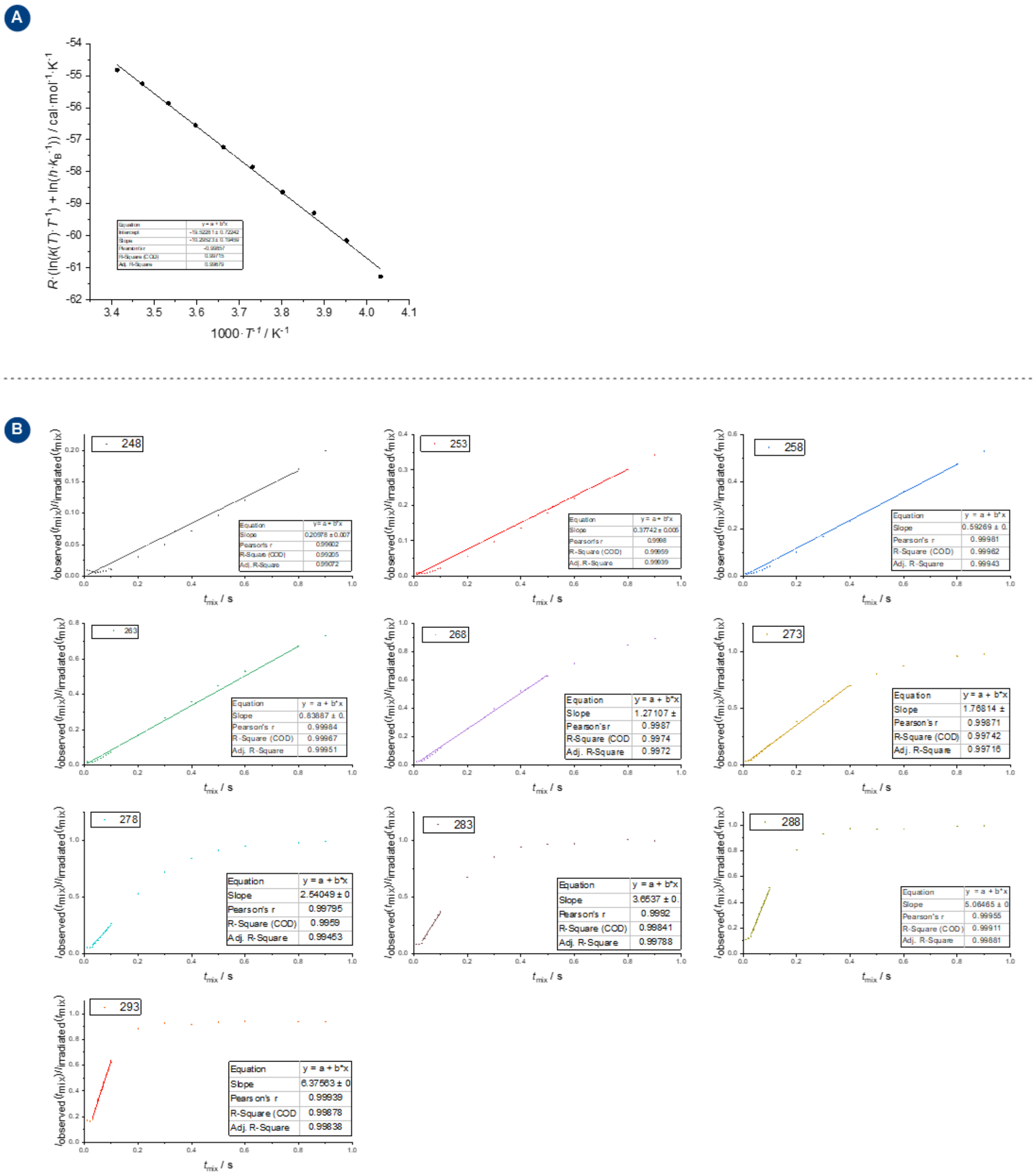

Figure S51 – Study of complex **1** in THF-d$_8$ and LiCl (14 equiv.). The sample was selectively excited at 4.99 ppm. Panel A: Eyring plot of complex **1** with LiCl according to equation 5. Slope = -Δ$H^‡$ ± 1.0 in kcal·mol^{-1} and intercept = Δ$S^‡$ ± 3.6 cal·mol^{-1}K^{-1}); panel B: overview of the EXSY build-up curves.

Table S6 – Values used for linear regression and error calculation in the EXSY temperature series of complex **1** with LiCl.

T / K	k / s^{-1}	1000·T^{-1} / K^{-1}	R·(ln(k(T)·T^{-1}) + ln(h·k_B^{-1})) / cal·mol^{-1}·K^{-1}	σ(Δ$H^‡$) / kcal·mol^{-1}	σ(Δ$S^‡$) / cal·mol^{-1}·K^{-1}
248	0.20978	4.032258	-61.27565	1.0	3.6
253	0.37742	3.952569	-60.14823	1.0	3.6
258	0.59269	3.875969	-59.29027	1.0	3.6
263	0.83887	3.802281	-58.63809	1.0	3.6
268	1.27107	3.731343	-57.84971	1.0	3.6
273	1.76814	3.663004	-57.23053	0.9	3.6
278	2.54049	3.597122	-56.54638	0.9	3.6
283	3.6537	3.533569	-55.85968	0.9	3.6
288	5.06465	3.472222	-55.24558	0.9	3.6
293	6.37563	3.412969	-54.82233	0.9	3.6
248	0.20978	4.032258	-61.27565	1.0	3.6

The activation parameters of $\Delta H^‡$ = 10.3 ± 1.0 kcal·mol^{-1} and the entropy $\Delta S^‡$ = -19.5 ± 3.6 cal·mol^{-1}·K^{-1} could hereby be estimated. A comparison of the individually calculated free enthalpy at different temperatures for both samples is visualized in Figure S52.

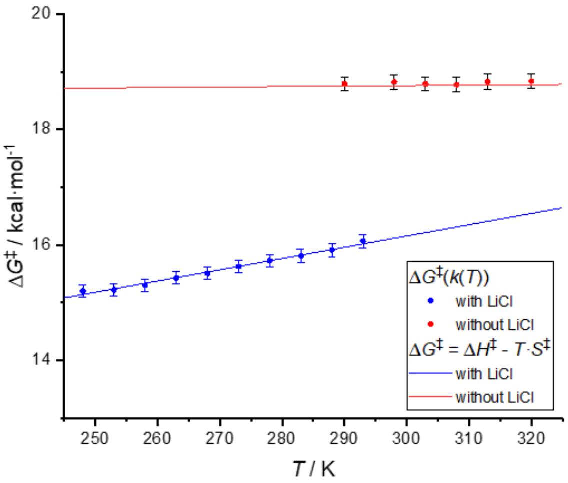

Figure S52 – Comparison of the calculated free activation enthalpy from individual rate constants with the activation entropy and enthalpy obtained from the VT experiments described above.

6. Quantitative NOESY

2D NOESY spectra[III] (*noesygpphzs*) with different mixing times were recorded (200, 300, 400, 500, 600, 700, 800 ms) in a randomized order. To obtain reliable NOE distances, normalization was done using the cross peak and diagonal peak volumes *A*, according to equation 9. The distances between protons *i* and *j* were obtained by referencing the build-up slope $\sigma_{\mathrm{i,j}}$ (equation 10, Figure S53) to an internal reference internuclear distance (r_{ref} = 1.78 Å) of two geminal protons (equation 11). At least four data points were used (data points were selected to improve R^2 and remove erroneous values) for the linear regression, and only values with $R^2 > 0.98$ were considered.

$$\eta_{\mathrm{NOE}} = \sqrt{\frac{A_{\mathrm{cross\ peak\ 1}} \cdot A_{\mathrm{cross\ peak\ 2}}}{A_{\mathrm{diagonal\ peak\ 1}} \cdot A_{\mathrm{diagonal\ peak\ 2}}}} \tag{9}$$

$$\eta_{\mathrm{NOE}} = \sigma_{\mathrm{i,j}} \cdot t_{\mathrm{mix}} \tag{10}$$

$$r_{\mathrm{i,j}} = r_{\mathrm{ref}} \left(\frac{\sigma_{\mathrm{ref}}}{\sigma_{\mathrm{i,j}}} \right)^{\frac{1}{6}} \tag{11}$$

[III] The following settings were used: 16 scans, 16 dummy scans, f2: 4096 datapoints, f1: 512 (zero filling applied in processing), no non-uniform sampling, relaxation delay (acquisition time + delay prior to start/repetition of pulse sequence) was adapted according to prior T_1 measurement. Values of a relaxation delay > 3-5 · T_1 are usually recommended, but even delays of > 1-2 · T_1 can reportedly lead to reliable quantitative information as a steady state is reached despite not recovering all magnetization.

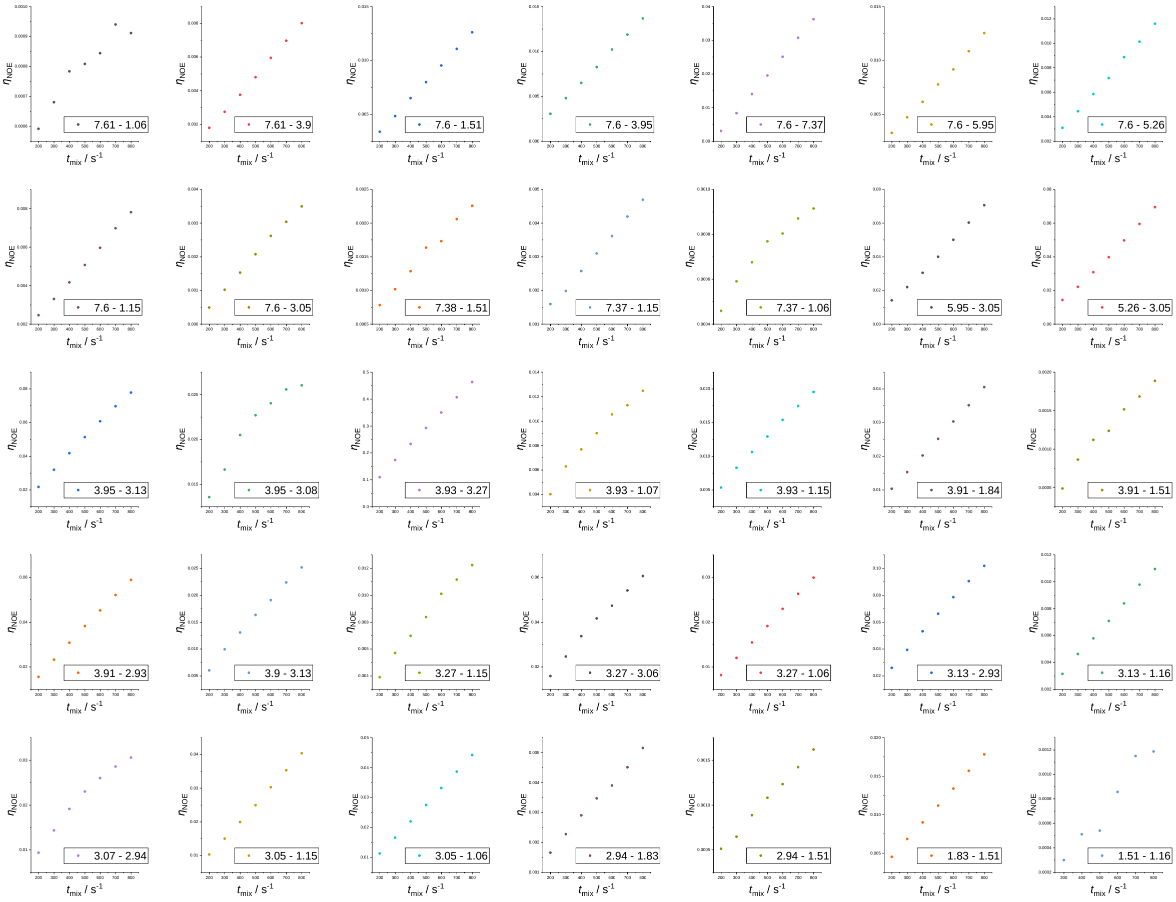

Figure S53 – Overview of NOE build-up of complex **2** for different $\delta_i - \delta_j$.

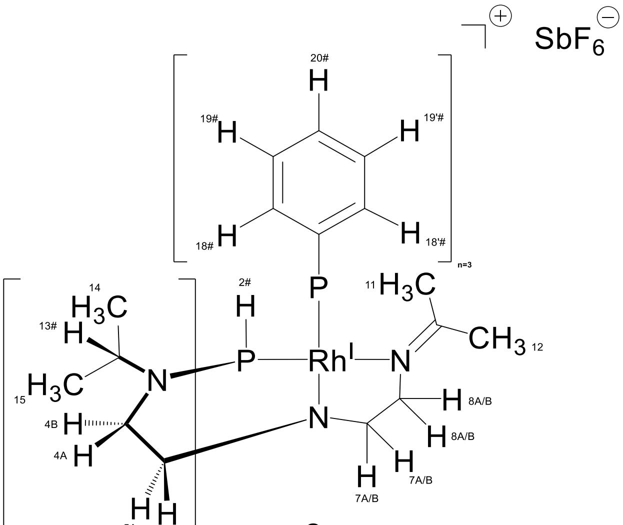

2

Figure S54 – Complex **2** with numbering from the assignment and indication of critical NOE distances.

Table S7 – NOESY derived distance information.

i	j	δ_i / ppm	δ_j / ppm	$\sigma_{i,j}$	R^2	$r_{i,j}$ / Å	$\sigma(r_{i,j})$ / Å
18#,18'#	15	7.61	1.06	9.6135E-07	0.99800	5.17	0.40
18#,18'#	8A,8B	7.61	3.91	9.8239E-06	0.99923	3.51	0.40
18#,18'#	11	7.61	1.51	1.5886E-05	0.99986	3.24	0.30
18#,18'#	4A	7.61	3.94	1.6819E-05	0.99943	3.21	0.30
18#,18'#	19#,19'#	7.61	7.37	5.5563E-05	0.99995	2.63	0.20
18#,18'#	2#	7.61	5.95	1.5530E-05	0.99986	3.25	0.30
18#,18'#	2#	7.61	5.26	1.4561E-05	0.99983	3.29	0.30
18#,18'#	14	7.61	1.15	1.0042E-05	0.99825	3.50	0.40
18#,18'#	13#	7.61	3.05	4.2440E-06	0.99673	4.04	0.40
19#,19'#	11	7.37	1.51	2.9930E-06	0.99460	4.28	0.40
19#,19'#	14	7.37	1.15	6.0796E-06	0.99703	3.81	0.40
19#,19'#	15	7.37	1.06	1.0158E-06	0.98985	5.13	0.40
2#	13#	5.95	3.05	8.3897E-05	0.99677	2.46	0.20
2#	13#	5.26	3.05	8.3055E-05	0.99763	2.46	0.20
4A	5A	3.93	3.13	1.0038E-04	0.99917	2.38	0.20
4A	5B	3.93	3.07	5.0460E-05	0.98500	2.67[b]	0.40
4A	4B	3.93	3.27	5.8038E-04	0.99993	1.78[a]	0.40
4A	15	3.93	1.06	1.8149E-05	0.99829	3.17	0.30
4A	14	3.93	1.15	2.5290E-05	0.99885	3.00	0.30
8A,8B	12	3.91	1.84	5.0483E-05	0.99997	2.67	0.20
8A,8B	11	3.91	1.51	2.4713E-06	0.99652	4.42	0.40
8A,8B	7A,7B	3.91	2.93	7.4900E-05	0.99967	2.50	0.20
8A,8B	5A	3.91	3.13	3.1917E-05	0.99969	2.89	0.20
4B	14	3.27	1.15	1.6315E-05	0.99609	3.23	0.30
4B	5B	3.27	3.06	7.8557E-05	0.99854	2.48	0.20
4B	15	3.27	1.06	3.7956E-05	0.99961	2.80	0.20
5A	7A,7B	3.13	2.93	1.2957E-04	0.99977	2.29	0.20
5A	14	3.13	1.16	1.4041E-05	0.99897	3.31	0.30
5B	7A,7B	3.07	2.93	4.5431E-05	0.99835	2.72	0.20
13#	14	3.05	1.15	5.0304E-05	0.99997	2.68	0.20
13#	15	3.05	1.06	5.5210E-05	0.99999	2.63	0.20
7A,7B	12	2.94	1.83	6.6625E-06	0.99649	3.75	0.40
7A,7B	11	2.94	1.51	2.0777E-06	0.99800	4.55	0.40
12	11	1.83	1.51	2.2346E-05	0.99998	3.06	0.30
11	14	1.51	1.16	1.4056E-06	0.99120	4.86	0.40

[a] reference distance, [b] alternate reference distance

Some distances in Table S7 were omitted for the comparison with experimental data, as they either gave no additional information (e.g., short-range $r_{18\#,19\#}$) or were less reliable due to severe overlap in the 2D

NOESY spectra. A similar error estimate, as suggested by *Snyder, et al.*[13] was used.[IV] Using an alternate reference distance (*ortho* and *meta* ^{1}H of phenyl substituent, $r = 2.5$ Å) leads to slightly different values within the estimated error margins.

To back-calculate theoretical distances to other nuclei, Tropp's method was chosen to average equivalent protons of methyl groups (assumption of slow tumbling), and further equivalent groups were also averaged according to equation 12 (assumption of fast tumbling) with r_i being the equivalent nuclei as implemented in Janocchio.[14]

$$r_{\text{averaged}} = \left(\frac{1}{\text{N}}\sum_{\text{i}}^{\text{N}} r_{\text{i}}^{-6}\right)^{-\frac{1}{6}} \tag{12}$$

[IV] Distances with respective error: $r < 3.0$, $\sigma(r) = 0.2$ Å; $3.0 \leq r < 3.5$, $\sigma(r) = 0.3$ Å; $3.5 \leq r < 6.0$, $\sigma(r) = 0.4$ Å.

Several rotamers and conformers of complex **2** were considered and obtained from the CREST-method (see section 7)[15] and which were used as input for Janocchio[14a] and DISCON.[16] Using only protons 7A/B and 8A/B as well as the methyl signals as equivalent nuclei, gave a more reliable result and only one side *a* or *b* was used as an input. The experimental and back-calculated values are shown in Table S8 and show quite a good fit, thus giving experimental solid evidence for the proposed structure of complex **2**.

Table S8 – Comparison of computational and experimental interproton distances as calculated by Janocchio from the DISCON derived ensemble.

i	*j*	r_{calc} / Å	$r_{i,j}$ / Å	$\|\Delta r\|$ / Å
15	18#,18'#	4.97	5.17	0.20
13#	18#,18'#	3.85	4.04	0.19
2#	18#,18'#	3.6	3.27	0.33
14	18#,18'#	3.29	3.5	0.21
11	19#,19'#	4.92	4.28	0.64
11	18#,18'#	3.54	3.24	0.30
14	19#,19'#	4.18	3.81	0.37
15	19#,19'#	5.73	5.13	0.60
2#	13#	2.7	2.46	0.24
11	14	5.14	4.86	0.28
12	8	2.63	2.67	0.04
8	11	4.65	4.42	0.23

The structures used by DISCON to obtain the averaged calculated distances were superimposed and show large similarities (Figure S55, panel A). The structures were further optimized at the D3BJ-PBE/def2-SVP level of theory and the proposed structure of complex **2** is shown in Figure S55, panel B.[V]

[V] Rotation away from the preferred arrangement of the isopropyl groups is possible but energetically unfavorable (short distance between 2# and 13#). Rotation around the Rh-PPh_3 bond happens fast relative to the NMR time scale (<<ms-µs) as does the interconversion of the quasi symmetric part *a* and *b*.

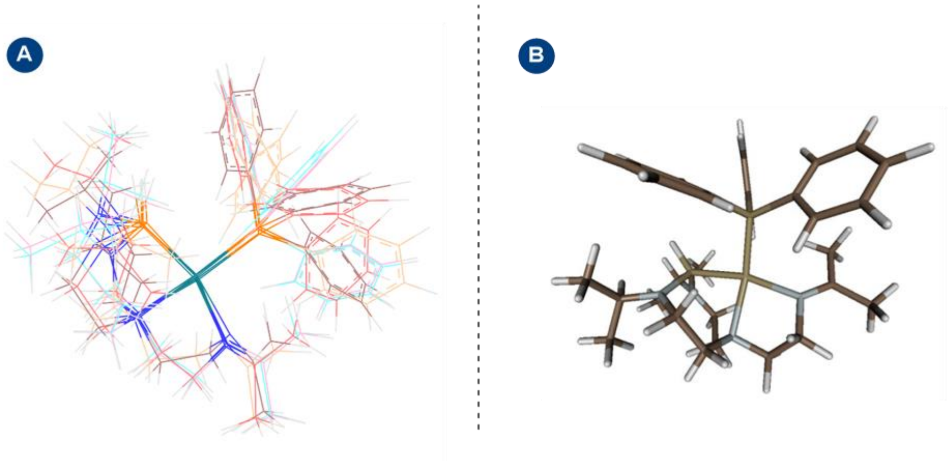

Figure S55 – Panel A: superposition of structures of complex **2** (xtb1 geometry level) used for calculating computational distances; panel B: computed structure of complex **2** at the D3BJ-PBE/def2-SVP level of theory.

7. Computational details

Conformational sampling was carried out by using the Conformer–Rotamer Ensemble Sampling Tool (CREST).[15] The xtb1[17] semi-empirical tight-binding method seemed to produce physically more sound conformers and was employed for the study of the monomer and a potential dimeric species of complex **2** using the following parameters (energy cut-off 6 kcal·mol^{-1}, RMSD = 0.25 Å, $T_{\mathrm{MD\ run}}$ = 400 K).[vi] These geometries were used as a first input for comparison with experimental NOE distances.

For all final structures presented in this report and as a basis for further energy calculations, the D3-PBE/def2-SVP method was selected. To validate the soundness of this structural method, the crystal structure of complex **1** was used and geometry optimized using different semi-empirical (xtb1 and xtb2), GGA, and hybrid GGA DFT methods (PBE/def2-SVP and B3LYP/def2-SVP) and the conductor-like polarizable continuum model (CPCM) for modeling solvation with THF. The DFT calculations here and further DFT calculations were improved by Grimme's D3 dispersion correction with Becke-Johnson damping (if applicable) and accelerated by the use of the resolution of identity approximation for Coulomb integrals and COSX numerical integration for the Hartree-Fock (HF) exchange as implemented in Orca 4.[18] For geometry optimization the def2-SVP basis set was chosen with the corresponding effective core potential def2-ECP to treat the rhodium core. All single point, geometry, frequency, and NMR calculations on the DFT or CCSD(T) level used the Grid6 and GridX7 settings to ensure sufficiently fine integration grids. In case of the absence of negative frequencies, calculated at the same level of theory, the root-mean-square deviation (RMSD) was calculated using VMD[19] to estimate geometric discrepancies (Figure S56). The PBE/def2-SVP method seemed to offer an adequate accuracy-to-computational-cost ratio.

[vi] Different alternate settings were assessed as well.

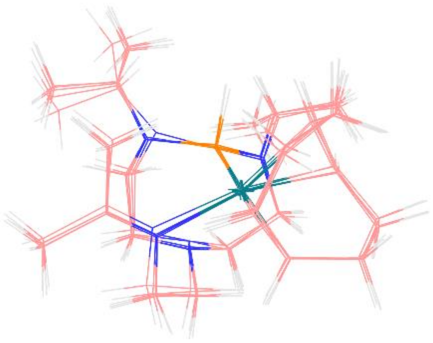

Method	RMSD[a] / Å
CPCM D3-B3LYP / def2-SVP	0.063
CPCM D3-PBE / def2-SVP	0.081
xtb1	0.134
CPCM xtb1	0.134
xtb2	0.192
CPCM xtb2	0.192

[a] compared to the crystal structure

Figure S56 – Superposition of the crystal structure and the calculated geometries.

For instance, crucial distances from the crystal structure (d(P-N_{ax})= 2.608 Å, d(P-Rh) = 2.304 Å) could be reproduced with sufficient precision using the protocol as mentioned earlier with the PBE functional (d(P- N_{ax})= 2.600 Å, d(P-Rh) = 2.338 Å).

To evaluate a method suitable for single-point energy calculations, the energy difference of complex **3** and **3'** (Figure S57) was compared for different GGA, meta-GGA, hybrid GGA, and long-range separated DFT methods with the def2-TZVP(-f) and the def2-TZVPP basis set and compared to the DPLNO-CCSD(T)/def2-TZVPP calculation. The validity of the CCSD(T) calculation was verified by the T_1 diagnostics[vii] that were below the recommended threshold (0.045 ≥ T_1) for fourth-row transition metal complexes.[20] ωB97X-D3BJ/def2-TZVPP appeared to be a reliable and stable method in this case and appeared to give consequent improvement upon increasing the basis set (Figure S58).

[vii] T_1 diagnostic values are 0.012836 (complex **3**) and 0.012594119 (complex **3'**).

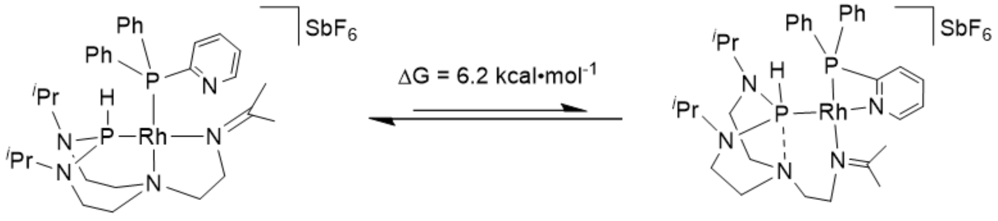

3 **3'**

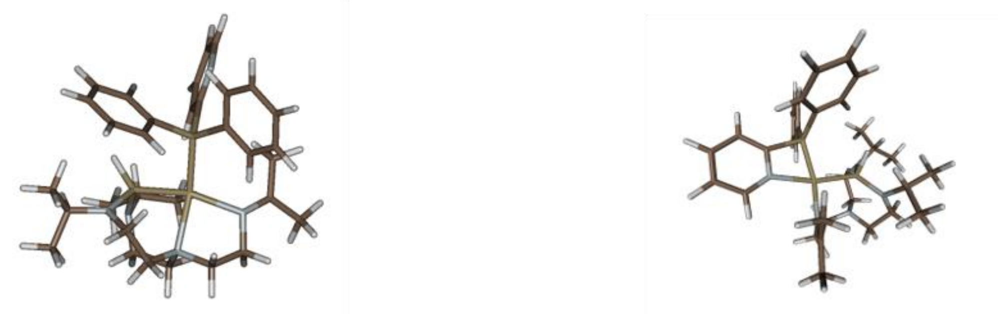

Figure S57 – Structural models of complex **3** and **3'**.

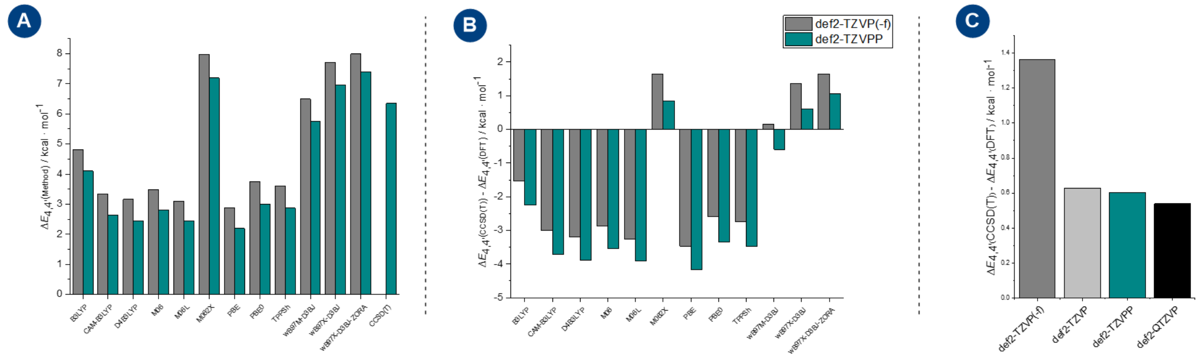

Figure S58 – Panel A: comparison of the energy difference of complex **3** and **3'** with different methods DFT/def2-TZVP(-f), DFT/def2-TZVPP, DFT/def2-TZVPP, CCSD(T)/def2-TZVPP; panel B: comparison of the energy difference of (a) with CCSD(T)/def2-TZVPP; panel C: comparison of the basis set dependence of ωB97X-D3BJ tested with def2-TZVP(-f), def2-TZVP, def2-TZVPP, def2-QVZP (on Rh, P, def2-TZVPP on light atoms). DFT for all calculations with D3 dispersion correction if not specified differently.

Finally, final energy calculations were carried out at the mentioned level of theory, if not specified otherwise, using the CPCM model for implicit solvation and the implemented D3BJ dispersion correction. For free enthalpies, the decomposition scheme using equations 13 and 14 were used.

$$H(\text{corrected}) = E_{\text{electronic,solvation}}(\text{high level}) + E_{\text{ZPE}}(\text{low level}) + \Delta U_{\text{thermal correction:rot.,vib.,trans}}(\text{low level}) + \Delta H_{\text{thermal enthalpy correction}}(\text{low level}) \quad (13)$$

$$G(\text{corrected}) = H(\text{corrected}) - T\Delta S_{\text{el.,vib.,trans,rot}}(\text{low level}) \quad (14)$$

As a high level of theory, ωB97X-D3BJ/def2-TZVPP was used, and PBE/def2-SVP represents the lower level of theory. In this regime, complex **3** is favored by a free enthalpy difference of 6.2 kcal·mol^{-1} over complex **3’**.

Geometries

The following xyz files contain the deometry-optimized structures at the D3-PBE/def2-SVP level of theory or the crystal structure (in case of complex **1**) of the relevant complexes. Note that complexes **1**, **2**, **3**, and **3’** show flexibility around the arylphosphine ligand and the xyz file is thus just a static picture of one exemplary stationary point of the rotameric ensemble at room temperature.

Complex 1 (crystal structure)

Rh	9.089	2.219	2.373
P	7.637	2.451	4.147
H	8.389	2.616	5.241
N	6.8	3.895	4.219
C	6.156	4.394	3.004
H	6.842	4.553	2.308
H	5.708	5.256	3.196
C	5.132	3.392	2.489
H	4.372	3.333	3.119
H	4.785	3.686	1.609
N	5.776	2.081	2.358
C	5.038	0.994	3.007
H	5.353	0.118	2.67
H	4.07	1.075	2.815
C	5.286	1.102	4.496
H	4.858	1.922	4.849
H	4.89	0.322	4.959
N	6.725	1.147	4.746
C	6.253	1.835	0.997
H	6.835	2.587	0.723
H	5.475	1.818	0.385
C	7.029	0.529	0.841
H	6.411	-0.235	0.967
H	7.396	0.475	-0.077
N	8.132	0.425	1.81
C	6.593	4.634	5.488
H	7.106	4.15	6.197

C	5.136	4.672	5.934
H	4.596	5.12	5.249
H	5.065	5.163	6.779
H	4.809	3.757	6.059
C	7.168	6.043	5.4
H	8.101	5.998	5.105
H	7.125	6.469	6.282
H	6.647	6.569	4.758
C	7.216	0.402	5.921
H	8.214	0.459	5.913
C	6.739	1.004	7.241
H	7.054	1.93	7.311
H	7.097	0.48	7.988
H	5.759	0.989	7.272
C	6.844	-1.077	5.824
H	5.888	-1.186	6.01
H	7.364	-1.589	6.478
H	7.04	-1.405	4.921
C	8.507	-0.724	2.243
C	9.613	-0.806	3.249
H	9.878	0.099	3.518
H	10.381	-1.268	2.853
H	9.303	-1.302	4.036
C	7.922	-2.042	1.813
H	7.052	-2.171	2.246
H	8.527	-2.768	2.072
H	7.806	-2.046	0.84
C	9.605	4.293	2.488
H	8.894	4.748	2.992
C	10.535	3.552	3.225
H	10.382	3.539	4.153
C	11.955	3.258	2.777

H	12.547	3.241	3.571
H	12.264	3.989	2.185
C	12.085	1.919	2.028
H	12.875	1.961	1.433
H	12.242	1.198	2.688
C	10.877	1.571	1.208
H	10.739	0.642	1.09
C	10.167	2.435	0.401
H	9.545	2.054	-0.196
C	10.545	3.881	0.155
H	10.337	4.116	-0.784
H	11.521	3.989	0.286
C	9.8	4.844	1.095
H	10.309	5.692	1.154
H	8.914	5.051	0.704

*SbF6 omitted

Complex 1 (optimized structure)

Rh	9.13493073703752	2.2058263584642	2.32356906481145
P	7.63203058885724	2.41283248031383	4.10193595986755
H	8.44042864773904	2.59850190183606	5.28410686406839
N	6.81923471065566	3.91811171367072	4.18962807274992
C	6.12080399957638	4.38659492426785	2.99734447855977
H	6.84590369879844	4.58566410619486	2.17589536215548
H	5.62333605954463	5.35179318266155	3.21375817042667
C	5.08901862154601	3.35751880948318	2.52788280564714
H	4.30420374351699	3.25472467299783	3.30239390022596
H	4.58703791950805	3.69367632026976	1.59144314433451
N	5.74871934577897	2.06157558745413	2.34424280782127
C	5.06043123647917	0.92962946395148	2.95922484779326

H	5.48330579646434	-0.00869229162345	2.55692968320099
H	3.96626821435715	0.92244032200536	2.73729977048335
C	5.30430428910852	0.95949948935147	4.46374844642146
H	4.71655062206973	1.78907273541674	4.92833115500652
H	4.91090274399003	0.02273321512103	4.90458753702892
N	6.73138318608983	1.06647903081679	4.75456388040824
C	6.26387823426332	1.88606993825865	0.98566522750310
H	6.91871033396416	2.75868767079760	0.77088554704563
H	5.44016863812907	1.89876279627973	0.23054156269589
C	7.06897515822825	0.60502854393037	0.76548995434474
H	6.40220053273104	-0.27889379955061	0.75819662049950
H	7.51296613526816	0.66443107798338	-0.25216141594990
N	8.14910882418649	0.44423788431150	1.73760458538592
C	6.61449983096900	4.62976650151424	5.46940111253166
H	7.19425841686691	4.05286811392294	6.22043435605466
C	5.14947381617654	4.64207654303422	5.92252557126248
H	4.50742862330306	5.20024986802662	5.21005362014890
H	5.05498004307908	5.13589573742086	6.91030117868830
H	4.75114520106892	3.61200505391783	6.01436715377132
C	7.21366724805289	6.04080131830305	5.41710933045843
H	8.28721864244778	6.00198543951601	5.14361337890294
H	7.12289929564738	6.53719615042284	6.40405040925509
H	6.68990745435230	6.67769704986707	4.67454015639176
C	7.20246412627797	0.46696366711882	6.02293027908589
H	8.29996389900846	0.64094928910010	6.04473561631179
C	6.59086613257808	1.12552778507607	7.26859051820099

H	6.80733905402563	2.21207823549111	7.29622825021864
H	7.00217671034166	0.67307080293636	8.19329021066832
H	5.48958750100470	0.99321953228777	7.29080098133166
C	6.99167279305283	-1.05266431784847	6.03681711147521
H	5.91901998473276	-1.32043311010933	6.12699763651141
H	7.51447043512065	-1.50115675124698	6.90485443754413
H	7.38317718268236	-1.51842114953117	5.11191124750045
C	8.46883236220857	-0.72932200827931	2.19838219775092
C	9.58156413010876	-0.84735082865674	3.19392810554366
H	9.91928769919196	0.15520392132877	3.53279449285650
H	10.44000329914952	-1.38635762554802	2.73863031007869
H	9.25694929861233	-1.45354487203256	4.06376289259234
C	7.78538112509106	-2.00261620021940	1.77196637856296
H	6.71453267073181	-1.99753915342890	2.06816045822950
H	8.26329262795826	-2.87870987974462	2.24539208591223
H	7.81826622285012	-2.13493376364826	0.67188423878885
C	9.62868566508879	4.28452335924551	2.49020157271045
H	8.83925199198596	4.77941742169256	3.08181524682543
C	10.57703421361518	3.52694555837298	3.22263849655471
H	10.42164101920750	3.47249632587946	4.31550717879580
C	12.00515595456383	3.24809133872846	2.76459604663738
H	12.67060537969227	3.22540734837520	3.65015745475428
H	12.36333751881992	4.08874037973948	2.13703588647668
C	12.13474864987146	1.91195283010811	2.00249092897117
H	13.04199351743702	1.91879744619042	1.35513397732094
H	12.28906416958048	1.08792726629726	2.72902715795203

C	10.90894443769561	1.57267432695732	1.18390257842303
H	10.75747651007494	0.49536100175015	0.99638153304673
C	10.17931134595460	2.46030378121579	0.37665868459031
H	9.48429344383754	2.01201713401430	-0.35508750958238
C	10.54930851365195	3.91484167694909	0.13506176838472
H	10.28433671735499	4.18676259052544	-0.90596006292884
H	11.64788456854222	4.03620659724443	0.21283269723308
C	9.82992448051351	4.87152451067598	1.10887960715851
H	10.37616332016814	5.84095841485710	1.17746726347464
H	8.82671073776383	5.12124293014363	0.70369774403567

Complex 2

C	-2.98701654378531	0.60164017328248	3.72504487240033
C	-1.95005027383286	1.47730615253476	4.38580752192004
C	-2.20041815623234	1.82370874684134	5.81795355540238
N	-0.87525012311625	1.89679296140995	3.78117414618099
C	-0.54066554938985	1.40940639214183	2.43665199157189
C	0.85935482603984	0.80865057960671	2.49625958973607
N	1.87640974402009	1.81594422830348	2.92049470684857
C	3.09780811918289	1.11877472704571	3.47659943531229
C	3.30632740356332	1.25021612242618	4.98409747701159
N	3.72599241545788	2.59336507110254	5.39199840212467
C	4.68980174731863	2.74430195374558	6.49424136244089
C	6.09225328922057	2.26552432932280	6.09359137154823
C	4.20239891222711	2.08335917530152	7.79117924361765
P	2.77588558995037	3.88073207582925	4.77923276165654
N	3.38814539387432	4.38762042414376	3.27204660163739

C	4.75309011964062	4.91221882657082	3.10837152459285
C	4.73843961228628	6.35160558543385	2.57632132656346
C	5.64381022501136	3.98865898592279	2.26701411482610
C	2.52097006030135	4.16578468774528	2.11765617868099
C	2.24739998410417	2.69361345346395	1.75764079922510
H	-3.25926780415392	0.96724539030078	2.71578641941382
H	-3.90239955632908	0.56085834189661	4.34289350935946
H	-2.61354243831824	-0.43886723960194	3.61507507868414
H	-2.36739452393908	0.88495105787167	6.38811004852367
H	-1.34868982291565	2.37323522568078	6.25497035829441
H	-3.12880570736145	2.42323683378293	5.91768832620291
H	-0.57873744056013	2.26078993572966	1.72400728178160
H	-1.24003200513342	0.63549333128916	2.06396321010010
H	0.85744903759465	-0.00439014518925	3.24805280002148
H	1.14001208791532	0.36374413266521	1.51540582309386
H	3.99098722785765	1.52184859977640	2.96414418942645
H	3.03307588316769	0.04294832390471	3.21428914588821
H	4.11141676167035	0.53527453124190	5.25142583623899
H	2.38531232504852	0.91726410961051	5.52149911834179
H	4.75155253300832	3.83922848470333	6.67968127202095
H	6.81696011172290	2.45531733446133	6.91087177793540
H	6.44276513299147	2.79358239704304	5.18475468565119
H	6.10583105257150	1.17513139693071	5.88623427530635
H	4.91433075800733	2.27596095651505	8.61875896678884
H	4.11375228116720	0.98313143376315	7.67724632888793
H	3.21107869778097	2.48177573064289	8.08566020187859

H	5.18078797230876	4.93924817694540	4.13490673888223
H	4.34816452580008	6.39312268653711	1.53802714005599
H	5.76309962012195	6.77493811100957	2.56341252681568
H	4.09331383953132	6.99197727101530	3.20954020338222
H	6.67509762786611	4.39080269874720	2.20454023200351
H	5.25919147152615	3.89484359061938	1.23005716291367
H	5.69529012765823	2.97543230721199	2.71271290698717
H	2.97209468610818	4.64994625447637	1.22716364139484
H	1.54192137641970	4.66393074261825	2.29360842204628
H	3.11834639551468	2.23660716058292	1.24116675328862
H	1.40608012760229	2.68727977935635	1.03748901661154
Rh	0.75136253210344	3.09515636446240	4.38615828939741
C	0.47346894649955	3.75632856601131	7.93190477548866
C	0.64015327448916	3.76229563070191	9.32629041575212
C	0.28211659562138	4.89899270185066	10.06921740703826
C	-0.23661271683046	6.03086005278090	9.41543657977610
C	-0.40297820037162	6.02708456583690	8.02199472404065
C	-0.05553330508093	4.88320629850116	7.26965356073560
P	-0.22654768816685	4.78665947003338	5.43608736181925
C	-2.03664553324822	4.98586351998842	5.09108632501201
C	-2.99051292685804	5.33319125368843	6.06885805709167
C	-4.35442026212320	5.39641040647837	5.73808440449155
C	-4.78107255631864	5.11862481091275	4.42918524624857
C	-3.83633227890667	4.77241846396590	3.44818816703375
C	-2.47533167829727	4.69829822357391	3.77947123379822
C	0.49795662282806	6.39924512362147	4.86237369571999

C	1.50098285032162	7.05572092590254	5.60885083976519
C	2.13765370237581	8.19836082354655	5.09672727776122
C	1.78314863690116	8.70183485164462	3.83537171156755
C	0.78408825908443	8.05549902316237	3.08745385407344
C	0.14806282792225	6.91296026348685	3.59459487929364
H	1.05601442331631	2.87780541771253	9.83263212769659
H	0.41330100092960	4.90768066167875	11.16203383834557
H	-0.50908627570241	6.92602251572151	9.99495505998267
H	-0.78983691167791	6.92613896639511	7.51838727674746
H	-2.67894685975182	5.53605740038627	7.10311911017227
H	-5.08879220411546	5.66065713358853	6.51432272782589
H	-5.85082375641501	5.16574862296873	4.17455772015651
H	-1.74117028287810	4.38256931859227	3.02169250801163
H	1.78860789907466	6.67958916244750	6.60139099958519
H	2.91629052579799	8.69768977096920	5.69348882106864
H	2.28252132347663	9.59734742950834	3.43565276780162
H	0.49456364198318	8.44425239276305	2.09916214981169
H	-0.63106355859274	6.42099603654392	2.99406666065603
H	3.24182527497360	4.89781614500827	5.67354377022930
H	-4.16117187380331	4.54536651065177	2.42128408108824
H	0.76513137334714	2.88139758060004	7.32534119339543

Complex 3

C	-0.95756492599659	-2.30830330971087	1.85815641554276
C	-0.18391328126545	-1.42204973178008	0.91818037014729
C	-0.78398838071085	-0.08829956256882	0.61442905683657
N	0.96568973385206	-1.78748058356708	0.41258279896208

C	1.45947142304834	-3.12060793661269	0.79864866590390
C	2.74098059432234	-3.46779715062396	0.07198034706329
N	3.79288064834128	-2.41844405779976	0.20239220470404
C	4.26509918479812	-2.28307391715756	1.60901273832581
C	5.34144093408971	-1.19965378351934	1.83385683652774
N	5.04431275341085	0.17453832006390	1.43584266578002
C	4.47524848426930	1.10676322297000	2.42948685785096
C	5.52072334872668	1.48381912387867	3.48553353300834
C	3.16468495340400	0.61479312464179	3.06431583692928
P	4.52832762678804	0.37237336946371	-0.21139146120158
N	5.70866496289621	-0.40855993152903	-1.12941288823340
C	7.08984943934112	0.09393408168480	-1.27287912053724
C	7.37390431508838	0.51917115540650	-2.71799726015733
C	8.14146418995615	-0.89379051372717	-0.75134008614313
C	5.28563047283481	-1.66799227900397	-1.74258787433762
C	4.93036532652914	-2.76155446518619	-0.73078573269863
H	-1.86725391856162	-1.80419411765634	2.22847383576014
H	-1.26074566431466	-3.24951874102243	1.35146007497472
H	-0.33705635506586	-2.60982128236841	2.72783955764142
H	-0.63546512024339	0.59038973274221	1.48271042813861
H	-1.87774201796910	-0.18096296002200	0.46280647737435
H	-0.32048617394898	0.38260419674268	-0.26551472278926
H	0.70764108205988	-3.90381284171022	0.56316298320128
H	1.60469990454841	-3.16221189673033	1.90016880109900
H	3.12441334532319	-4.44594994397723	0.44349188748798
H	2.53177697075671	-3.57441543522117	-1.01017801230073

H	6.27385427068993	-1.51919703641316	1.32575322543723
H	5.57707911538127	-1.20789042255413	2.91655799935049
H	4.24500835851650	2.03180978326579	1.85240745651357
H	5.11616815970003	2.25483654982754	4.17140660395072
H	6.43646989830168	1.88057088179307	3.00520684096773
H	5.80409276792919	0.60717181711829	4.10434784452815
H	2.69186385077652	1.41750741474812	3.66539261183606
H	3.34268429055348	-0.24638469457060	3.74087100416509
H	2.45414789090907	0.29437535884484	2.26961740204504
H	7.13635438525629	0.99956008760631	-0.63047035223037
H	8.38232863904200	0.97251104510330	-2.80016809841137
H	6.62273397184958	1.25477409841945	-3.06327578028751
H	7.34130164665884	-0.35202298762583	-3.40522081393388
H	7.98059394723727	-1.13261419505035	0.31818004667841
H	9.15623760071147	-0.46026071236081	-0.85388229374867
H	8.12686286577035	-1.84179629803210	-1.32800431788493
H	6.10762171459165	-2.05120008941047	-2.38015862069417
H	4.40895080494122	-1.49358222041796	-2.40507964609658
H	5.82049932971632	-3.02600390155204	-0.12584268592283
H	4.64818177891720	-3.67194215173186	-1.29492743642833
Rh	2.58909294632108	-0.60453570711546	-0.37243455059808
N	0.22664457026139	3.33608025945461	-1.58793424371552
C	-0.24724033219316	4.44789653036662	-1.00076267205064
C	0.08208577789222	4.82730946542707	0.31223448028745
C	0.94061335720248	3.99971929305084	1.05044121768381
C	1.41700050735106	2.82417754574107	0.45136568670294

C	1.04273294546494	2.54115898561612	-0.87564176918227
P	1.72700890395557	0.98468373018369	-1.66637694474150
C	0.41187539927809	0.43968971712321	-2.84851725875451
C	-0.09881685171013	-0.87043766201058	-2.75319284854181
C	-1.07290896867486	-1.32096448272162	-3.65927045332183
C	-1.54048725127684	-0.46557529494794	-4.66996632654875
C	-1.02919610376387	0.84022922812995	-4.77396783987615
C	-0.05530925731553	1.29391791137751	-3.87165329185792
C	2.96081891607118	1.71382496331360	-2.83936234079072
C	3.41186421750576	0.92000947865986	-3.91690947589191
C	4.37792694756921	1.41158425849468	-4.80437150826954
C	4.92119553137287	2.69597990357124	-4.61720034815739
C	4.49192162011049	3.48249549870470	-3.53747366472267
C	3.51316677882529	2.99708206542343	-2.65191216716358
H	-0.92420735181821	5.07490629949932	-1.60733202929246
H	-0.33051377138657	5.75205574718454	0.74174207263771
H	1.22213311106278	4.25615228127443	2.08322793993213
H	2.05133699968291	2.10807934086218	0.99388122105762
H	0.28048740612204	-1.53663194140100	-1.96284131263535
H	-1.46296995426036	-2.34704823204697	-3.57684823473164
H	-2.30148484987465	-0.81761401898726	-5.38331992816620
H	-1.38916426702564	1.51127771150405	-5.56902395352993
H	0.34161964760301	2.31428810890906	-3.95397582167047
H	2.99068734275470	-0.08659591899961	-4.06734131081391
H	4.71236705482040	0.78778846580067	-5.64727745432882
H	5.68134463983230	3.08092386214610	-5.31371887858582

H	4.91273378526730	4.48791721230206	-3.38360211671327
H	3.17115240705623	3.63409722706849	-1.82268946148713
H	4.80719575134117	1.77280541769229	-0.35974206609882
H	4.70008481368045	-3.25428100531718	1.94359815128515
H	3.38332843713533	-2.07729045844091	2.24259329795760

Complex 3’

C	0.26815262961202	-3.55917372227859	2.56029980081006
C	0.57053921043717	-2.32636508569728	1.74823658798361
C	-0.18812036821064	-1.07668611909089	2.07549268017336
N	1.44014328074177	-2.30785667365507	0.77984073834867
C	2.18167226691318	-3.52352735581303	0.43839633997592
C	3.53399329042676	-3.25037081828712	-0.21454093354169
N	4.44637396702158	-2.40612669891613	0.55292159558288
C	4.61383788064008	-2.67586606837621	1.97542389464043
C	4.89129764890409	-1.36263313960531	2.70363495299986
N	3.88733872338774	-0.36301206316435	2.36939359385603
C	3.52716851980423	0.61729128220600	3.40977002792814
C	4.72112151886850	1.46656906670552	3.87271816754227
C	2.80844259911626	-0.05282664946480	4.58780732117254
P	3.58339551695133	0.06872281197308	0.70186673466036
N	5.01943591329847	0.26503294011136	-0.22059560659291
C	5.90662282999580	1.42939792079472	-0.04905262386455
C	6.14721911781921	2.14521434237701	-1.38666844573910
C	7.22499988912564	1.08889244372397	0.65921145510184
C	5.47101832069650	-0.85539506850751	-1.03433054543701
C	5.67789704325981	-2.11023698600933	-0.17972064606471

H	-0.53922226172643	-3.36315639105521	3.28818677607066
H	-0.03631962327333	-4.40740096375447	1.91374361586635
H	1.16561674160414	-3.89175823668619	3.12499611667352
H	0.22109617737435	-0.21776293813871	1.49902054044058
H	-0.14191970513233	-0.87109530315207	3.16515840782357
H	-1.26276329585422	-1.21189528420231	1.82513056722040
H	1.58116786976495	-4.11100666730486	-0.29113807858371
H	2.30423517166028	-4.17624975995309	1.32620317450899
H	3.99433068255076	-4.24294680840666	-0.44859272725200
H	3.35352701510091	-2.73295106849779	-1.18281848361255
H	5.92926393288548	-1.01400489675104	2.47398646624361
H	4.87092462019715	-1.55487697018654	3.79524655579563
H	2.79104132418889	1.30087896594765	2.93334770708611
H	4.40138837712416	2.23051308886448	4.61008070111849
H	5.18923300877123	1.99116241246878	3.01524343790693
H	5.49799747591711	0.83843674135738	4.35556643713783
H	2.43785616668475	0.71059383228574	5.30119866499010
H	3.48390639067394	-0.72895751354096	5.15188522293543
H	1.94662367159560	-0.64874792918344	4.22880882779963
H	5.34432447482849	2.12960982946654	0.60630249167393
H	6.74897992539226	1.52147151605599	-2.07945820326175
H	6.70037598337963	3.09253670831157	-1.22693607912585
H	5.18789913812835	2.37292895067200	-1.89277409933988
H	7.83389441876181	2.00284391370057	0.81212604954117
H	7.83361826401398	0.38089710577256	0.05888651930610
H	7.03732693091629	0.62946837232297	1.65008379709925

H	6.41444823188340	-0.58772691934537	-1.54909390426057
H	4.72838390448569	-1.07153209195162	-1.83353172320234
H	6.49260895709993	-1.92201888765592	0.54673001354519
H	5.99234853112945	-2.97438112733933	-0.81159558986745
Rh	1.76431697353034	-0.59481597549517	-0.39951418731724
N	-0.01309333010545	-0.73351999203454	-1.59296417640668
C	-1.04965759695202	-1.57641176633318	-1.75637076991931
C	-2.06683327640862	-1.30876812402137	-2.68866514576858
C	-1.99802345762052	-0.14646779016234	-3.47030491841074
C	-0.91178410288607	0.73554105877953	-3.30012968531439
C	0.05052930293986	0.40870918144135	-2.34570158348066
P	1.63470572537655	1.16779629268882	-1.75993288781712
C	1.25645299124271	2.81728902465938	-1.06469120500963
C	-0.06606485608991	3.22489182209679	-0.79213388356429
C	-0.30682819760561	4.44497736650564	-0.14069736965710
C	0.76619115201153	5.26361275661979	0.24993197328181
C	2.08600730234428	4.85952172316404	-0.01425123686413
C	2.33315609569078	3.64195253635802	-0.66545322281802
C	2.65604409836748	1.48505280856136	-3.25283836923161
C	3.35416259197495	0.38685649206293	-3.79700761390890
C	4.15247609743782	0.55250743316392	-4.93754475663581
C	4.26604783765270	1.81930581779371	-5.53627849234507
C	3.56884022590862	2.91503742511166	-4.99999634982011
C	2.76093143812320	2.75186261335446	-3.86306437724747
H	-1.05717203133017	-2.48100441177010	-1.12848553580126
H	-2.90181387129178	-2.01548643288490	-2.79788008654848

H	-2.78214597325816	0.07525391371612	-4.20942556096644
H	-0.81775108969446	1.65593932530804	-3.89451116621121
H	-0.91387484891992	2.58810148549273	-1.08727159832937
H	-1.34256221293617	4.75753655235207	0.06150007371041
H	0.57384246835333	6.21915135475619	0.76078523430715
H	2.93150338027690	5.49574440056793	0.28840741214601
H	3.37066926006772	3.33749997634427	-0.87201842262984
H	3.26646343935673	-0.59705531949430	-3.30814385392297
H	4.69664040327325	-0.30823731568816	-5.35501901668510
H	4.90192572958067	1.95345423632437	-6.42460768719705
H	3.65341692922315	3.90682130730300	-5.46969293853535
H	2.21852159956082	3.61449541839767	-3.44819098139798
H	3.40542371587672	1.47312924995222	0.99127268694983
H	5.42355895374474	-3.41808490534733	2.18289263840820
H	3.67152483424681	-3.09625557879132	2.37273876914515

8. X-ray diffraction studies

Crystals of complex **1** and complex **2** were covered with polyfluorinated polyether and selected under a microscope with an applied nitrogen cryo-stream at about -40 °C in polarized light. They were picked up with nylon loops and rapidly mounted in the nitrogen cold gas stream of the diffractometers at 100 K. A Bruker Kappa Mach3 APEX-II diffractometer with a Bruker IμS x-ray source was used for complex **1**, whereas a Bruker D8 Venture diffractometer with IμS3 Diamond source was used for data collection of complex **2**. Both instruments were equipped with INCOATEC Helios mirror optics (Mo-Kα radiation; λ=0.71073 Å). Final cell constants were obtained from least-squares fits of setting angles of several thousand strong reflections. Intensity data were corrected for absorption using intensities of redundant reflections using SADABS.[21] The structures were readily solved by Direct and Patterson methods and subsequent difference Fourier techniques. The Bruker APEX3[22] software package was used for solving and refining the structures. All non-hydrogen atoms were anisotropically refined, and hydrogen atoms were placed at calculated positions and refined as riding atoms with isotropic displacement parameters. Crystal and data collection details are given in Table S9. Complex **2** crystallizes in chiral space group P2(1) with four crystallographically independent complex molecules, four SbF_6-anions, and two molecules of dichloromethane in the asymmetric unit. The crystal was found to be an inversion twin, and data were refined accordingly. The PN_4-ligand showed disorder problems in three of the four molecules. A split model was refined for two of them, and further restraints were applied on the third to keep displacement parameters in an acceptable range. One molecule containing Rh(3) was found to be only very slightly disordered. The SbF_6-anion containing Sb(50) was also refined by a two-position split model due to disorder. The scattering contributions of the two disordered dichloromethane molecules were removed using Platon/SQUEEZE program.[23]

Table S9 - Crystallographic data of complexes complex **1** and complex **2**.

	complex **1**	complex **2**
Formula	$C_{23}H_{45}F_6N_4PRhSb$	$C_{33}H_{48}F_6N_4P_2RhSb$
M_r in g mol^{-1}	747.26	901.35
Color, habit	orange, prism	yellow, needle
Crystal system	orthorhombic	monoclinic
Space group	*Pbca*	$P2_1$
a in Å	10.6090(6)	9.3423(5)
b in Å	20.4571(12)	36.870(2)
c in Å	26.0864(15)	22.441(12)
β in °	90	91.937(2)
V in Å^3	5661.5(6)	7725.5(7)
Z	8	8
T in K	100(2)	100(2)
Crystal size in mm^3	0.071 × 0.042 × 0.041	0.12 × 0.02 × 0.02
ρ_c in g cm^{-3}	1.753	1.550
F(000)	3008	3632
Diffractometer	Bruker-AXS Kappa Mach3 APEX-II	Bruker-D8 Venture
$\lambda_{XK\alpha}$ in Å	X = Mo 0.71073	X = Mo 0.71073
θ_{min} in °<br>θ_{max} in °	1.561<br>35.100	1.889<br>30.000
Index range	$-17 \leq h \leq 17$<br>$-32 \leq k \leq 32$<br>$-42 \leq l \leq 42$	$-13 \leq h \leq 11$<br>$-51 \leq k \leq 50$<br>$-31 \leq l \leq 31$
μ in mm^{-1}	1.653	1.266
Abs. correction	multi-Scan	multi-Scan
Reflections collected	216260	192479
Reflections unique	12490	45033
R_{int}	0.0393	0.0583
Reflections obs. [F>2σ(F)]	10883	37789
Residual density in e Å^{-3}	1.335/-0.962	1.870/–1.405
Params/restraints	346/0	1764/1106
GOOF	1.135	1.031
R_1 [I>2σ(I)]	0.0248	0.0479
wR_2 (all data)	0.0557	0.1019
CCDC	2100809	2100808

9. References

[1] a) J. Tang, J. G. Verkade, *Tetrahedron Lett.* **1993**, *34*, 2903-2904; b) A. E. Wróblewski, J. Pinkas, J. G. Verkade, *Main Group Chem.* **1995**, *1*, 69-79; c) P. B. Kisanga, J. G. Verkade, *Tetrahedron* **2001**, *57*, 467-475.

[2] A. Jerschow, N. Müller, *J. Magn. Reson.* **1997**, *125*, 372-375.

[3] *Topspin*; Bruker BioSpin GmbH.

[4] *MestReNova*; Mestrelab Research S.L.: Santiago de Compostella, Spain, 2020.

[5] D. W. Marquardt, *J. Soc. Ind. Appl. Math.* **1963**, *11*, 431-441.

[6] *OriginPro*; OriginLab Corporation: Northampto, MA, USA, 2019.

[7] M. J. Thrippleton, J. Keeler, *Angew. Chem., Int. Ed.* **2003**, *42*, 3938-3941.

[8] a) M. H. Haindl, J. Hioe, R. M. Gschwind, *J. Am. Chem. Soc.* **2015**, *137*, 12835-12842; b) H. Jangra, M. H. Haindl, F. Achrainer, J. Hioe, R. M. Gschwind, H. Zipse, *Chem. - Eur. J.* **2016**, *22*, 13328-13335.

[9] H. Hu, K. Krishnamurthy, *J. Magn. Reson.* **2006**, *182*, 173-177.

[10] N. Juranic, Z. Zolnai, S. Macura, *J. Serb. Chem. Soc.* **2000**, *65*, 285.

[11] A. D. Bain, G. J. Duns, Chapter 12 Chemical exchange measurements in NMR, In *Analytical Spectroscopy Library*, Elsevier, 1997; Vol. 8, pp 227-263.

[12] P. M. Morse, M. D. Spencer, S. R. Wilson, G. S. Girolami, *Organometallics* **1994**, *13*, 1646-1655.

[13] N. Nevins, D. Cicero, J. P. Snyder, *J. Org. Chem.* **1999**, *64*, 3979-3986.

[14] a) D. A. Evans, M. J. Bodkin, S. R. Baker, G. J. Sharman, *Magn. Reson. Chem.* **2007**, *45*, 595-600; b) A. Kolmer, L. J. Edwards, I. Kuprov, C. M. Thiele, *J. Magn. Reson.* **2015**, *261*, 101-109.

[15] P. Pracht, F. Bohle, S. Grimme, *Phys. Chem. Chem. Phys.* **2020**, *22*, 7169-7192.

[16] O. Atasoylu, G. Furst, C. Risatti, A. B. Smith, 3rd, *Org. Lett.* **2010**, *12*, 1788-1791.

[17] C. Bannwarth, E. Caldeweyher, S. Ehlert, A. Hansen, P. Pracht, J. Seibert, S. Spicher, S. Grimme, *WIREs Comput. Mol. Sci.* **2021**, *11*, e1493.

[18] F. Neese, *WIREs Comput. Mol. Sci.* **2018**, *8*, e1327.

[19] W. Humphrey, A. Dalke, K. Schulten, *J. Mol. Graph.* **1996**, *14*, 33-38.

[20] J. Wang, S. Manivasagam, A. K. Wilson, *J. Chem. Theory Comput.* **2015**, *11*, 5865-5872.

[21] L. Krause, R. Herbst-Irmer, G. M. Sheldrick, D. Stalke, *J. Appl. Crystallogr.* **2015**, *48*, 3-10.

[22] *APEX3 v2019.11-0 program package*, v2019.11-0; Bruker AXS: Karlsruhe, Germany, 2019.

[23] a) A. Spek, *Acta Crystallogr. D* **2009**, *65*, 148-155; b) *Platon – A Multipurpose Crystallographic Tool*; Utrecht University: Utrecht, The Netherlands, 2011.
